# Mild and Efficient Heterogeneous Hydrogenation of Nitroarenes Facilitated by a Pyrolytically Activated Dinuclear Ni(II)-Ce(III) Diimine Complex

**DOI:** 10.3390/ijms23158742

**Published:** 2022-08-05

**Authors:** Jessica Michalke, Kirill Faust, Thomas Bögl, Stephan Bartling, Nils Rockstroh, Christoph Topf

**Affiliations:** 1 Institute of Catalysis (INCA), Johannes Kepler University (JKU), Altenbergerstraße 69, 4040 Linz, Austria; 2 Institute of Inorganic Chemistry, Johannes Kepler University (JKU), Altenbergerstraße 69, 4040 Linz, Austria; 3 Department of Analytical Chemistry, Johannes Kepler University (JKU), Altenbergerstraße 69, 4040 Linz, Austria; 4 Leibniz Institute for Catalysis, University of Rostock (LIKAT Rostock), Albert-Einstein-Straße 29a, 18059 Rostock, Germany

**Keywords:** heterogeneous hydrogenation, nickel, cerium, diimine complexes, pyrolysis

## Abstract

We communicate the assembly of a solid, Ce-promoted Ni-based composite that was applied as catalyst for the hydrogenation of nitroarenes to afford the corresponding organic amines. The catalytically active material described herein was obtained through pyrolysis of a SiO_2_-pellet-supported bimetallic Ni-Ce complex that was readily synthesized prior to use from a MeO-functionalized salen congener, Ni(OAc)_2_·4 H_2_O, and Ce(NO_3_)_3_·6 H_2_O. Rewardingly, the requisite ligand for the pertinent solution phase precursor was accessible upon straightforward and time-saving imine condensation of *ortho*-vanillin with 1,3-diamino-2,2′-dimethylpropane. The introduced catalytic protocol is operationally simple in that the whole reaction set-up is quickly put together on the bench without the need of cumbersome handling in a glovebox or related containment systems. Moreover, the advantageous geometry and compact-sized nature of the used pellets renders the catalyst separation and recycling exceptionally easy.

## 1. Introduction

The production of aniline derivatives from the respective NO_2_-tagged arenes is a large-scale process of paramount importance given the steadily rising request for organic amines that are used in the manufacture of medicines, azo dyes, agricultural chemicals, and precursors to polycondensates such as polyamides. In a classic method, the reduction of the pertinent nitro motif is brought about by hazardous, aqueous mineral acid in combination with profligate amounts of powdered iron [1]. Yet, the green chemistry aspiration of avoiding excessive waste calls for the implementation of less polluting, catalytic strategies that employ convenient and cost-effective reagents [2]. In this sense, molecular hydrogen fits well as a reducing agent by virtue of its good abundance and excellent operability. Moreover, H_2_ gas allows for atom-economic syntheses of anilines that only produce benign and low-molecular-weight side-products so as to obtain decent E Factors [3,4,5,6,7].

With respect to homogeneous catalysis, the scientific literature gives accounts of the utilization of noble metals including Au [8], Ir [9], Pd [10,11], Pt [12], Rh [13], and Ru [14,15,16], while efficacious non-precious-metal-based approaches rely on Fe [14,17,18], Mn [19], or Co complexes [20] that drive the hydrogenation of organic nitro compounds to yield the wanted amines.

Concerning heterogeneous catalysis, precious-metal-containing Pd/C [5], Pt/C [21], or supported Au [22] are reliable catalysts for the title reaction whereas prominent noble-metal-free routes to anilines from nitrobenzenes encompass catalyst formulations based on Co [23,24,25,26,27,28] or particulate Ni catalysts [29,30]. Be that as it may, the most frequently used (industrial) solid catalyst for the hydrogenation of substituted nitroarenes is, by virtue of its low cost and excellent activity, fine-grained Raney nickel [31,32,33,34]. In spite of these beneficial traits, the utilization of this spongy metal is, owing to its pyrophoricity, connected with inherent safety risks and improper use of this material has already led to serious accidents [35,36]. For safety reasons, this catalyst must thus be stored (and bought) as a suspension in water which severely hampers the exact dosing of small, catalytic amounts. A further crucial disadvantage of Raney Ni is hydrodehalogenation which occurs as a notable side reaction in the hydrogenation of halonitroarenes such that dedicated inhibitors have to be added in order to ameliorate the chemoselectivity [30]. It is for these reasons that the development of more selective, safer, and robust Raney nickel surrogates is still highly sought-after and rewarding.

In a somewhat different context, Beller and coworkers popularized the pyrolytic syntheses of solid, multicomponent redox catalysts that turned out to be very potent in the nitroarene-to-aniline hydrogenation. This pyrolysis approach relies on the thermal disintegration of a molecularly well-defined transition metal complex that was previously adsorbed onto a proper supporting material through wet impregnation. Controlled heat treatment of the loaded support under an inert gas atmosphere and subsequent cooling to room temperature leaves behind a composite material that is directly usable without further activation steps. Initial pertinent reports introduced cobalt(II) and iron(II) acetate-phenanthroline combinations for the creation of heterogeneous catalysts that enabled a convenient method for the syntheses of various organic amines [37,38,39]. Following these findings, a great deal of reports on the preparation of cobalt-based nanocomposites and their use in the reduction of nitroarenes either by H_2_ gas or proper hydrogen transfer reagents (hydrazine, formic acid) have emerged [40,41,42,43,44,45,46,47,48,49,50,51,52,53,54,55,56,57,58,59,60,61,62,63]. It was established that the activity of these materials is heavily dependent on the nitrogen concentration [64] and, hence, the deployment of N-rich polydentate chelators is a promising means for the manufacture of empowered heterogeneous catalysts that are activated through annealing.

**Figure 1:**
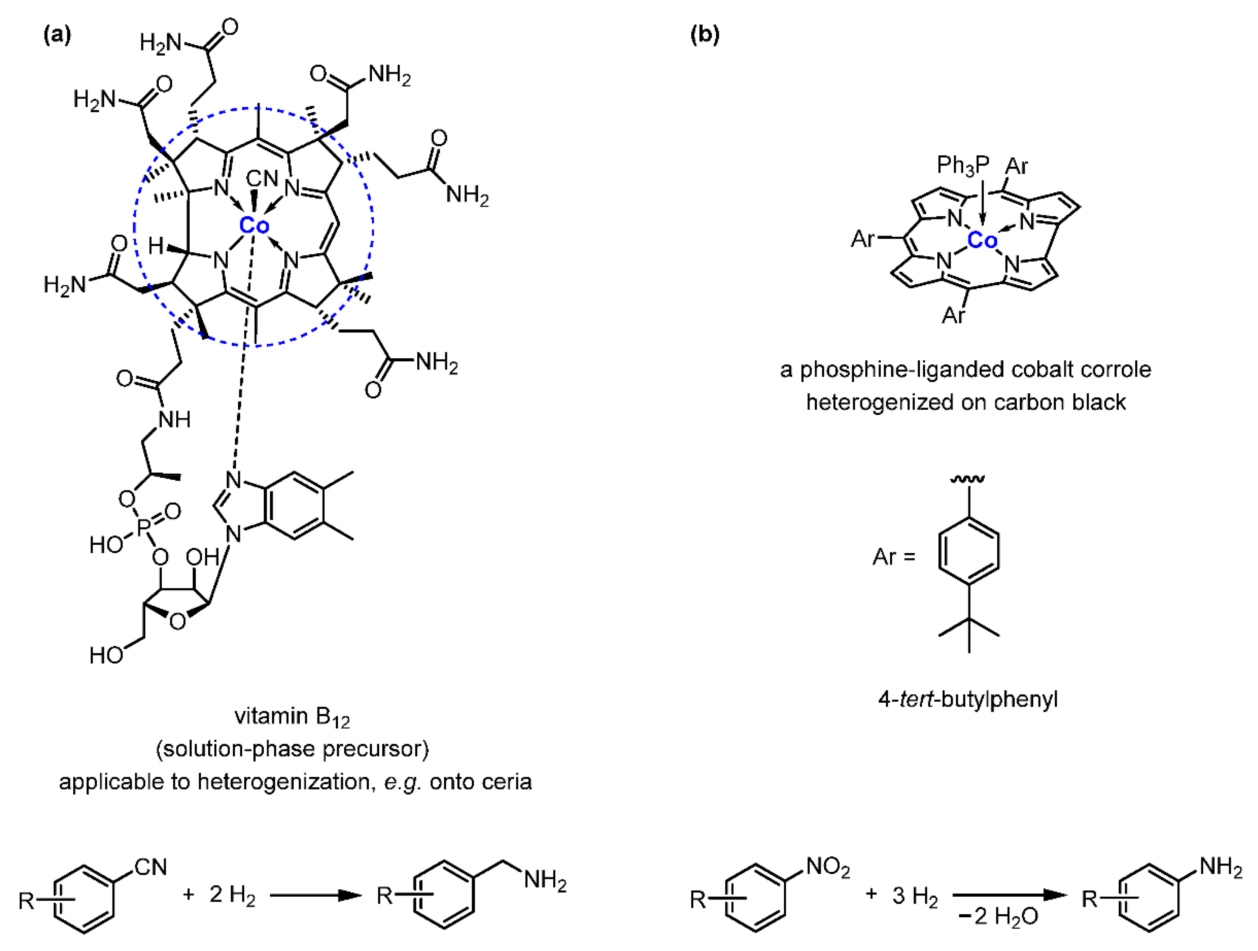
(**a**) Natural cyanocobalamin (vitamin B_12_) was shown earlier to function as a raw material for making solid hydrogenation catalysts that enable the syntheses of benzylamines from the respective benzonitriles; the encircled group marks the corrin core. (**b**) The molecularly well-defined vitamin B_12_ substitute [Co(corrole)(PPh_3_)] serves as a base substance for composites that catalyze reduction of nitroarenes with gaseous H_2_ to afford the more truncated anilines.

For the purpose of dispensing with the need for a time-wasting in situ complex generation that precedes the impregnation of the carrier material, commercial vitamin B_12_ (cyanocobalamin, Figure 1a) can be used as solution phase precursor without prior chemical modification. Related materials that are based on this natural coenzyme are applicable in the reduction of O_2_ [65], in the evolution of H_2_ [66], in fuel cells [67], or in organic syntheses for the production of imines [68] and benzylamines [69] (Figure 1a).

Despite the usefulness of vitamin B_12_ for the design of novel catalytically active composites, the pyrolysis of this biomolecule is a rather extravagant process since it is associated with the complete eradication of valuable and unique stereocenters. For a more rational catalyst synthesis, the structural similarity of the corrin core structure of cyanocobalamin (marked region in Figure 1a) and the corrole skeleton then served as a leitmotif for the preparation of vitamin B_12_ surrogates that are dominated by the [Co(corrole)] subunit. The synthesis and characterization of corroles is a well-studied field [70,71,72,73,74,75,76] and appropriate complexes figure prominently as discrete catalytically active entities in such diverse processes ranging from the splitting of H_2_O [77], O_2_-related redox-transformations [78,79,80], and electroreduction of CO_2_ [81,82] to the homogeneous hydrogenation of nitrobenzenes [20]. Additionally, their use in CO gas sensors [83] and in the detection of nitrite/nitrate ions [84,85] has been described. A recent publication on a thermally activated, PPh_3_-functionalized cobalt corrole that proved very effective in the heterogeneous nitroarene-to-aniline hydrogenation expanded the application range of this versatile macrocyclic compound class (Figure 1b) [86]. As was to be expected, the extra axial phosphine ligand permitted a brisk access to *N*, *P*-co-doped solid catalysts in which the incorporated P atoms significantly improved the redox properties of the material [87,88,89,90,91,92,93,94,95,96,97,98,99,100,101,102,103,104,105,106].

However, the supply and replenishment of sufficient amounts of the ligand-functionalized [Co(corrole)] complexes that are needed for the production of the heterogeneous catalysts are still tedious processes [70,71].

**Figure 2:**
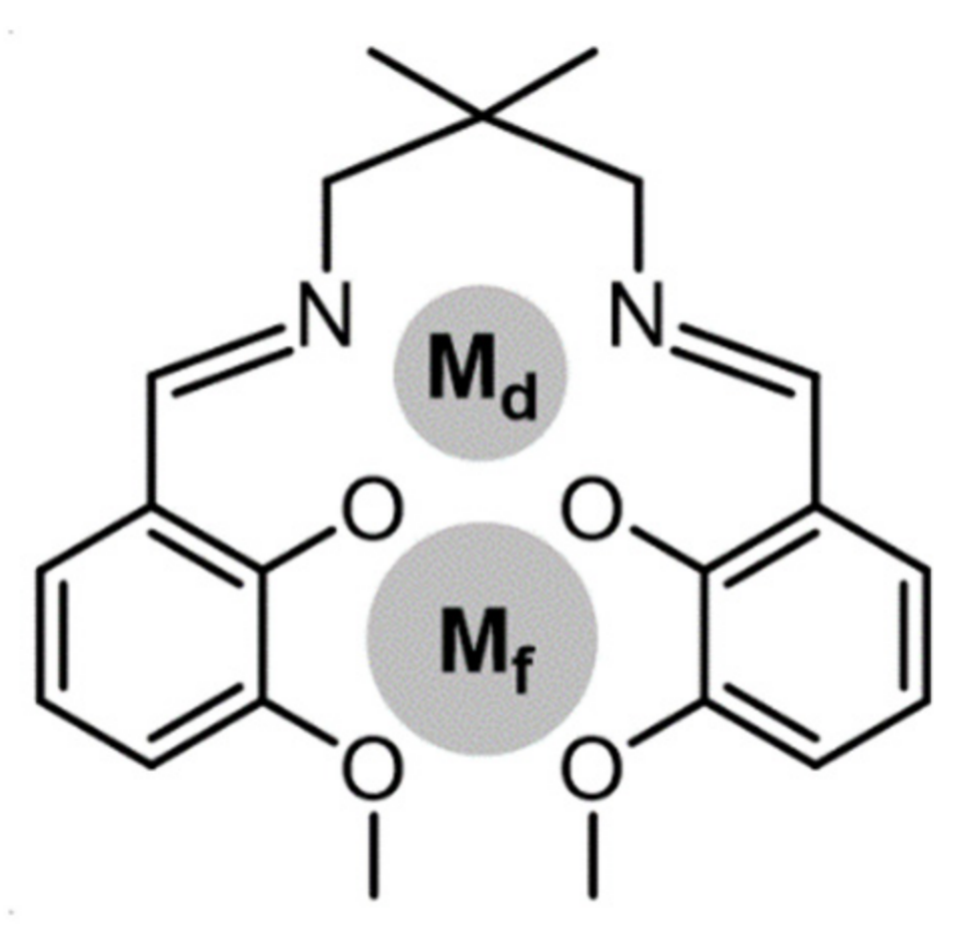
Stylized representation of the molecular architecture of a metalliferous coordination compound with the general formula [(L)M_d_M_f_]. The respective complex served as a solution phase precursor for the manufacture of a heterogeneous hydrogenation catalyst (see main text). L denotes a methoxy-functionalized salen-type ligand that provides two different binding compartments; M_d_ = 4th row transition metal cation; M_f_ = 6th row transition metal (lanthanide) cation. For the sake of clarity, the associated counter anion is not drawn.

Herein, we describe the establishment of the common and easily accessible salen motif [107,108] as a general scaffold for crafting catalytically active hybrid materials. The organic backbone of this ligand class is amenable to peripheral editing such that additional donors, e.g., alkoxy groups, can be introduced. The thus resulting augmented chelator is capable of hosting not just one, but two active metal centers in close vicinity such that mutual interaction between the two metals becomes possible. In this context, it was found that a methoxy-functionalized salen-type ligand enables the quick assembly of heterodinuclear d-f metal complexes [109]. Therein, the extended salen platform possesses an inner chelating site which comprises two N- and two O-donor atoms that tightly bind to 3d ions and, in addition, an outer coordination sphere exists, which readily accommodates 4f cations (lanthanides) by virtue of the four O-donors (Figure 2) [110,111,112]. While the physicochemical properties of these mixed metal complexes have been exploited in a variety of luminescent, magnetic, and electronic applications [113,114,115], their implementation in a method for the fabrication of solid mixed-metal hydrogenation catalysts has escaped attention so far and, accordingly, their huge potential for the utilization in the context of organic syntheses is far from being exhausted. In order to remedy the severe underutilization of this auspicious substance class, the original intention of our approach was to include both a predefined redox center (Ni) and an immediately adjacent Lewis-acidic metal cation (Ce^3+^) into one functional composite such that the various catalytically active entities will be able to act in a cooperative manner. The choice of this particular metal combination was guided by the fact that nickel plays a prominent role in hydrogenation reactions (*vide supra*) [116,117,118] and in the manufacture of (structurally demanding) amines [119], while cerium cations are famous for their oxophilicity [120]. Accordingly, we anticipated an approaching NO_2_-containing educt to coordinate to the Ce^3+^ sites, where invoked, surface Ni-H species would transfer their nucleophilic hydride to the (tethered and activated) proximate nitro substrates so as to furnish the respective anilines in a facile manner.

## 2. Results and Discussion

### 2.1. Ligand Synthesis

**Scheme 1:**
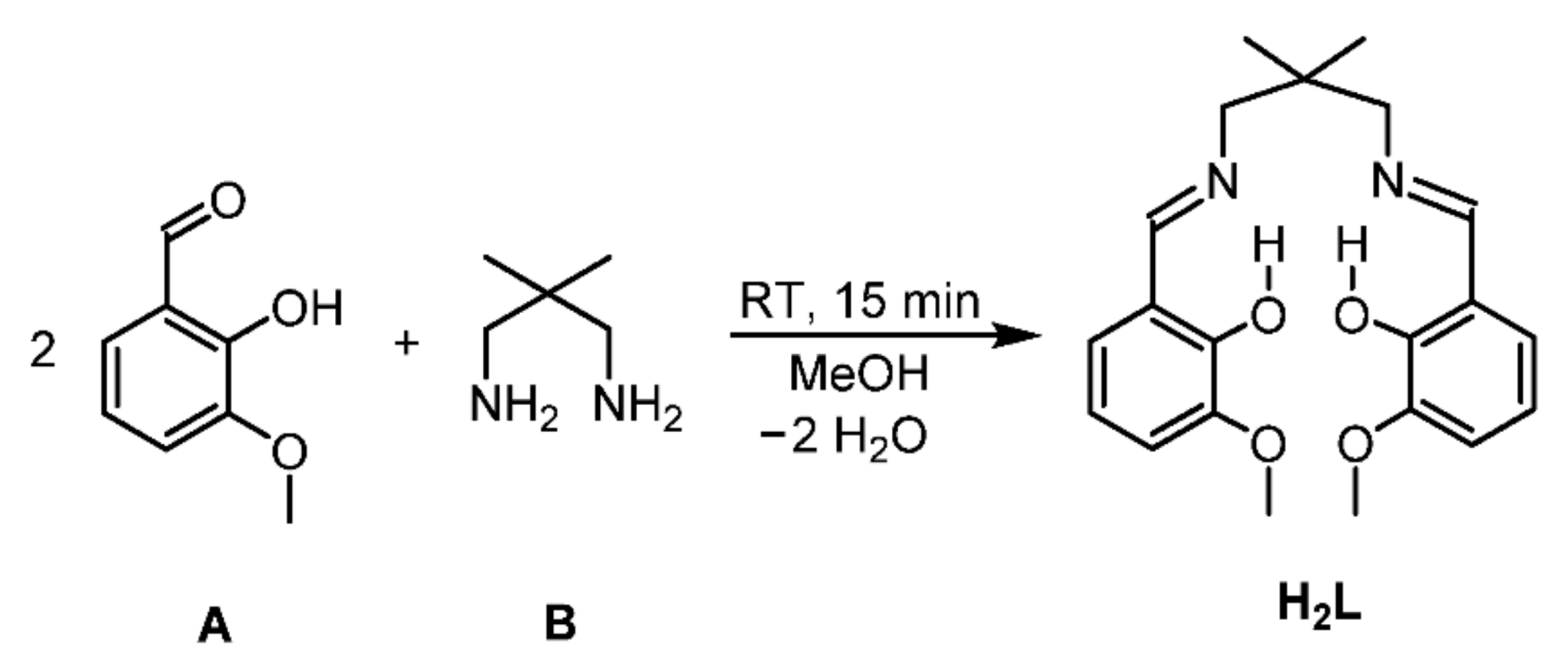
One-step synthesis of the salen-type ligand **H_2_L** through reaction of MeO-functionalized salicylaldehyde **A** (*o*-vanillin) with a simple aliphatic diamine **B**. This straightforward imine condensation is carried out without any external heat sources. RT: room temperature.

The requisite binucleating ligand **H_2_L** is readily assembled prior to use upon reaction of *ortho*-vanillin **A** with 1,3-diamino-2,2′-dimethylpropane **B** in methanol (Scheme 1). Of note, this imine condensation is accomplished within less than one hour at room temperature (RT) and without the necessity of an exogeneous heating source. Furthermore, the experimental procedure is free from any laborious isolation and purification steps. These very important practical features are in stark contrast to those pertaining to the syntheses of the abovementioned corrole derivatives that depend on a time-consuming protocol including a prolonged refluxing period (at significantly higher temperature) and laborious column chromatography to obtain pure fractions of the respective (metalated) macrocycles.

### 2.2. Formation of the Heterobimetallic Complex

**Scheme 2:**
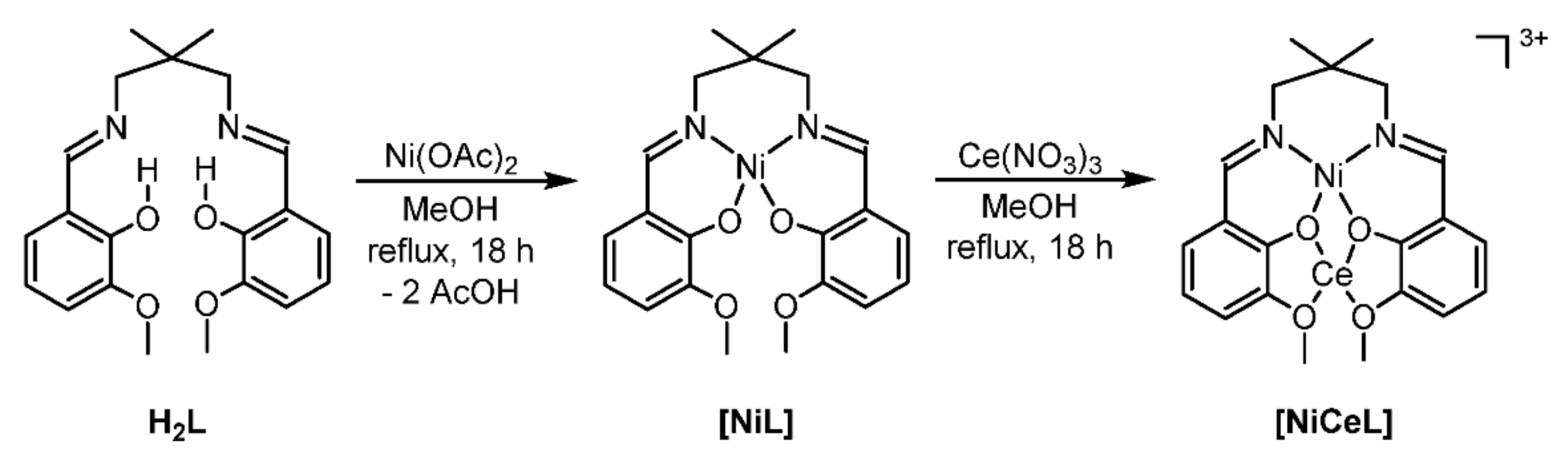
Syntheses of monometallic complexes **[NiL]** and the heterobimetallic congener **[NiCeL]** from the extended salen-type ligand **H_2_L** using commercial and readily available Ni(OAc)_2_·4 H_2_O and Ce(NO_3_)_3_·6 H_2_O, respectively. The NO_3_^−^ counter anion of compound **[NiCeL]** as well as the crystal water molecules of the applied Ni and Ce salt are omitted in the drawings for the sake of simplicity.

**Figure 3:**
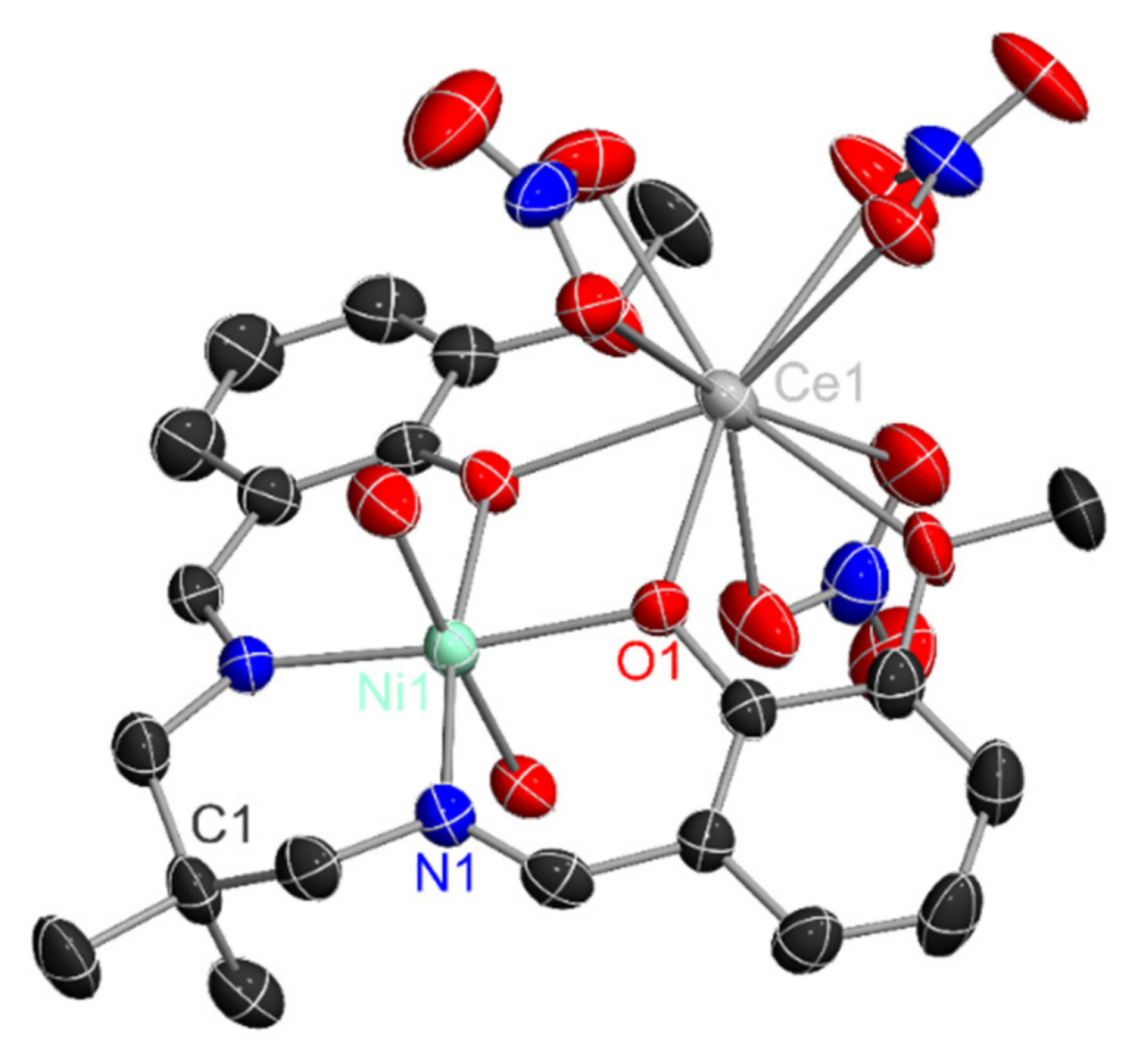
Molecular structure of dinuclear **[NiCeL]** as determined by single crystal X-ray diffraction analyses. The axial ligands that are linked to the Ni center stem from the crystal water in the applied nickel precursor. The thermal ellipsoids were drawn at the 50% probability level and the H atoms are omitted for the sake of simplicity.

The prepared multidentate ligand **H_2_L** was used without further modifications to obtain the starting material for the solid composite catalysts (vide infra) in two steps. Metalation of the given chelating agent with Ni(OAc)_2_·4 H_2_O produced the neutral monometallic complex **[NiL]** while consecutive reaction of the latter with Ce(NO_3_)_3_·6 H_2_O neatly furnished the lanthanide-modified ionic compound **[NiCeL]** (Scheme 2). Crystals suitable for X-ray diffraction analysis were obtained upon slow diffusion of diethylether into a solution of **[NiCeL]** in acetone. Notably, it was found that the three NO_3_^−^ counter anions were bound to the Ce^3+^ center (Figure 3). Tabular X-ray crystallographic data are provided in Table S6 in the Supplementary Materials and Table S5 summarizes the results of the bulk elemental analysis of precursor **[NiCeL]**.

It is worth mentioning here that the N_2_O_2_ donor arrangement of this ligand platform is also known to ligate Cu^2+^ or VO^2+^ whereas the O_4_-based binding cavity was shown to function as a host for the Gd^3+^ cation [109].

### 2.3. Catalyst Preparation and Characterization

In the first instance, a pelletized SiO_2_ support was imbued with a solution of **[NiCeL]** in methanol upon which the volatiles were removed in vacuo. Hereafter, controlled pyrolysis under a gentle stream of argon at 800 °C [86] and subsequent cooling to ambient temperature afforded the ready-to-use catalyst **NiCeL@SiO_2_-pellet-800**. The procedural details for the manufacture of this active composite are outlined in Section 3.1.

**Figure 4:**
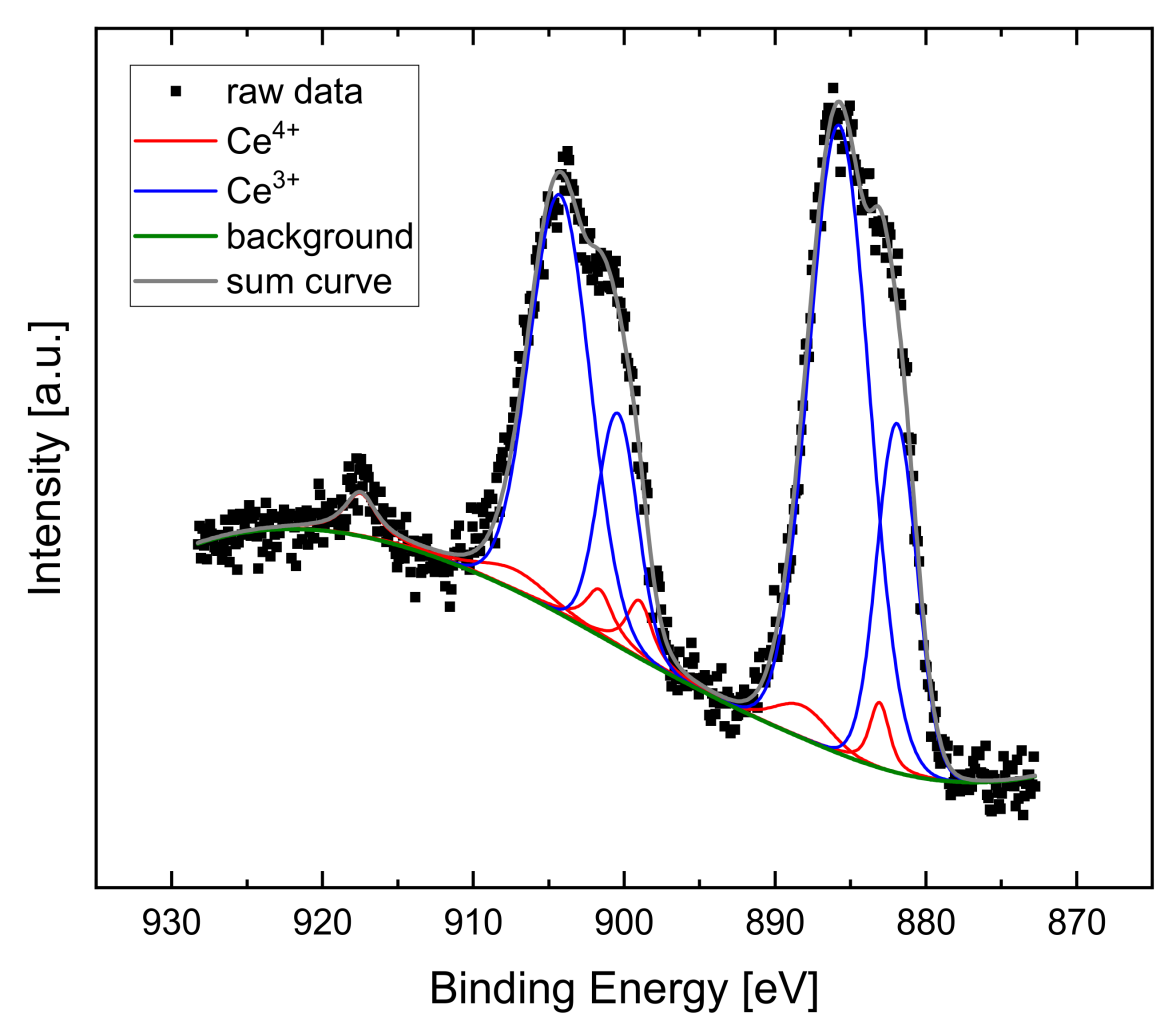
Ce 3d X-ray photoelectron spectrum of **NiCeL@SiO_2_-pellet-800** showing Ce^3+^ as main component.

The bulk elemental analysis (EA) of the obtained material revealed 3.00% Ce, 1.05% Ni, 1.03% C, 0.32% H, and 0.04% N. Presumably, the exceptionally low nitrogen concentration was caused by the conversion of the N atoms into gaseous, volatile products upon intrinsic oxidation through the abundant nitrate ions that are firmly incorporated in the solution phase precursor (Figure 3). It has to be noted here that the minor N content is well in accord with the results from X-ray photoelectron spectroscopy (XPS) since no spectroscopic feature was assignable to this element in the survey spectrum around 400 eV binding energy (see Figure S1). The XPS measurements further disclosed that cerium was present mainly as Ce^3+^ and only to a minor extent as Ce^4+^. The Ce 3d region in Figure 4 was deconvoluted with 10 peaks [121]. The main components at 881.9 eV and 885.8 eV belong to the v^0^ and v’ Ce 3d_5/2_ components of Ce^3+^, respectively. The corresponding Ce 3d_3/2_ components u^0^ and u’ can be found at 900.4 eV and 904.3 eV, respectively. A good indication for the presence of Ce^4+^ is the u’’’ peak at 917.5 eV which is only very weak in this case, thus confirming Ce^3+^ as the main oxidation state. The Ni 2p region partially overlaps with the Ce 3d which complicates the analysis. However, for the sample **NiCeL@SiO_2_-pellet-800**, the detected Ni concentration was too little for further analysis (see Figure S4).

For the sake of completeness, the C 1s and O 1s XPS spectra of **NiCeL@SiO_2_-pellet-800** are shown in Figure S2 and Figure S3, respectively.

**Figure 5:**
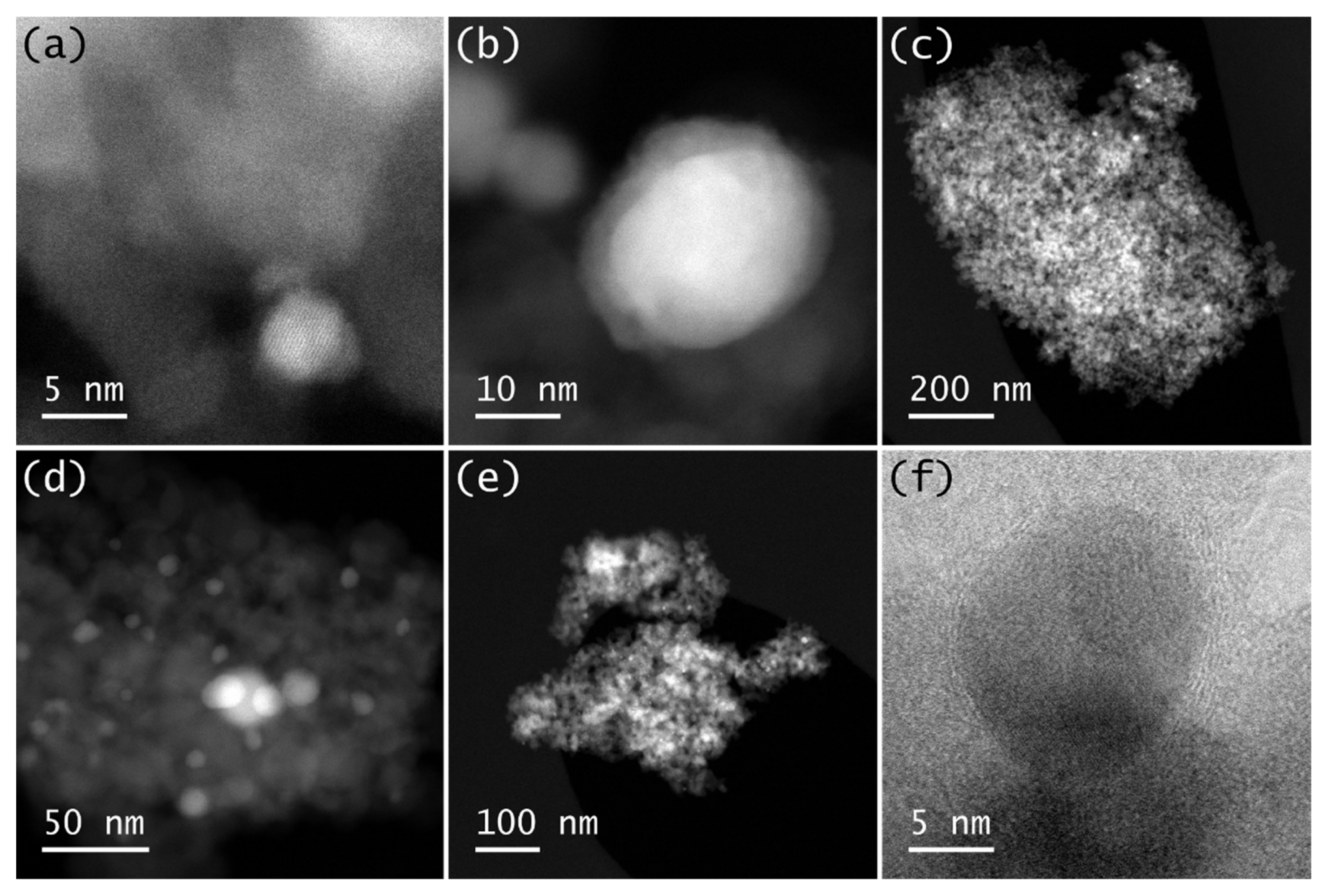
Representative HAADF-STEM micrographs (**a**–**e**) and a selected BF STEM micrograph (**f**) of **NiCeL@SiO_2_-pellet-800**.

Then, high-angle annular dark-field (HAADF) and bright field (BF) scanning transmission electron microscopy (STEM) with energy-dispersive X-ray spectroscopy (EDX) was deployed to obtain detailed information on the texture and composition of **NiCeL@SiO_2_-pellet-800** (Figure 5). The Ni-based nanoparticles were found to be present in three different size domains, i.e., small (2–7 nm, Figure 5a), medium (15–25 nm, Figure 5b), and large (centered around 30 nm, Figure 5d) with the small-particle fraction dominating the sample. Ni is predominantly present in its metallic state, and sometimes, this Ni is either surrounded by an oxide layer (Figure 5b) or carbon (Figure 5f).

Interestingly and in stark contrast to the results of the EA/XPS analyses (Table S4), cerium was observed only once and in trace amounts via HAADF-STEM and EDX (see Figure S5). Potential explanations for this behavior can be explained either by the fine-dispersed nature of the related particles or by the formation of rather large separate CeO_x_ particles. The latter would constitute most of the containing Ce and, due to their size, only a few particles would be present. In return, this number could be too small and thus could be overseen in STEM. Given the fact that we were previously able to detect microdispersed cerium in a related (hitherto unpublished) CuCe merged catalyst, we have to conclude that huge Ce-based particles are formed during pyrolysis.

### 2.4. Catalytic Tests

We commenced our study with the testing of different common supports, viz. SiO_2_, Al_2_O_3_, CeO_2_, Vulcan^®^ XC 72 R (all in powdered form), and pelletized SiO_2_ (Table 1). As clearly indicated, the latter largely surpassed the former (entry 1 versus entries 2–5) whereas Vulcan- and ceria-supported composites did not even show any catalytic activity at all. Importantly, the pellet catalyst was approximately six times more effective than the powdered congener (entries 1–2) and hence the implementation of an easy-to-separate, fairly active, and reusable material could be realized within this research project (see Section 2.6). In addition, we found that neither the unsupported nor the non-heat-treated **[NiCeL]** solution phase precursor (entries 6–7) catalyzed the title transformation. The influences of the physical reaction parameters (H_2_ pressure, reaction temperature) on the catalyst performance of **NiCeL@SiO_2_-pellet-800** are summarized in Table S1.

Having identified the optimal carrier material, we strove towards complete substrate conversion under a mild reaction temperature regime (60 °C) by raising the catalyst loading. In this context, we established that the application of 2.0 mol% of the solid catalyst **NiCeL@SiO_2_-pellet-800** already gave rise to complete substrate conversion in the nitrobenzene-to-aniline model hydrogenation. Indeed, owing to the varying weight and coarse-grained texture of the applied pellets, precision dosing of the active material in the mg scale was unfeasible. Hence, the catalyst amount was not fixed to a certain, constant value but ranged from 2.0–3.5 mol% throughout this study.

With reference to our previous work on a cobalt-corrole-based hydrogenation catalyst [86], we started to anneal the **[NiCeL]**-loaded SiO_2_ pellets at 800 °C (Table 2). This approach translated into 60% isolated yield of aniline whereas applying less temperature or no pyrolysis at all prohibited the isolation of any product (entries 1–3 and entry 5, respectively). Moreover, heat treatment of the impregnated support at a higher temperature also drastically diminished the extent of aniline formation (entry 4) and thus we decided to abide by the canonical 800 °C value. The complete thermogravimetric analysis of **[NiCeL]** is outlined in Figure S6 in the Supplementary Materials.

Next, we worked out the ideal reaction medium for the given nitroarene hydrogenation; as illustrated by Table 3, protic methanol (MeOH) is superior to strongly Lewis basic (entries 5 and 6), etheric (entries 7–9), and chlorinated solvents (entries 10 and 11). Only nonpolar *n*-heptane was able to keep up with MeOH, but using the former caused the precipitation of the product that seemed to block the active centers of the catalyst and, accordingly, we never observed full conversion in this case. As a consequence, we chose methanol as the working solvent for the elaboration of the substrate scope (vide infra).

Then, for highlighting the promoting effect of the incorporated cerium ions in **NiCeL@SiO_2_-pellet-800**, we conducted comparison experiments with the monometallic, heterogenized **[NiL]**-based congener. Importantly, it turned out that the latter was clearly outperformed by the standard NiCe catalyst (Table S2, entries 1 and 2). After that, the **NiL@SiO_2_-pellet**-mediated hydrogenation reaction was repeated, but this time with the separate addition of catalytic amounts of commercial Ce(NO_3_)_3_·6 H_2_O. As a result, we did not observe any activity-enhancing effect of the added lanthanide salt. The application of stoichiometric amounts of the pertinent nitrate even impaired the performance of the only-nickel catalyst (Table S3, entries 7 and 8). This experimental series unequivocally demonstrated that the proper functioning of the NiCe catalyst hinges upon an intricate embedding of the cerium ions into catalyst fabric that is, obviously, only achieved upon careful, thermal decomposition of the SiO_2_-supported **[NiCeL]** solution phase precursor. Generally, the established positive effect of the Ce-doped transition metal catalyst parallels the findings of Liu and Chen [122] who deployed a lanthanum-augmented NiB alloy for the hydrogenation of *p*-chloronitrobenzene.

The preliminary investigations were finalized by the hot filtration test as introduced by Maitlis [123]; catalyst removal from the reaction mixture pertaining to the nitrobenzene-to-aniline reduction and resumption of the hydrogenation procedure with the remaining solution did not produce any further portion of organic amine. Thus, we can reliably infer that the catalytic transformation is not supported by solubilized Ni- and/or Ce-based complexes but rather proceeds via an authentic, heterogeneous scenario.

### 2.5. Scope and Limitations

**Scheme 3:**
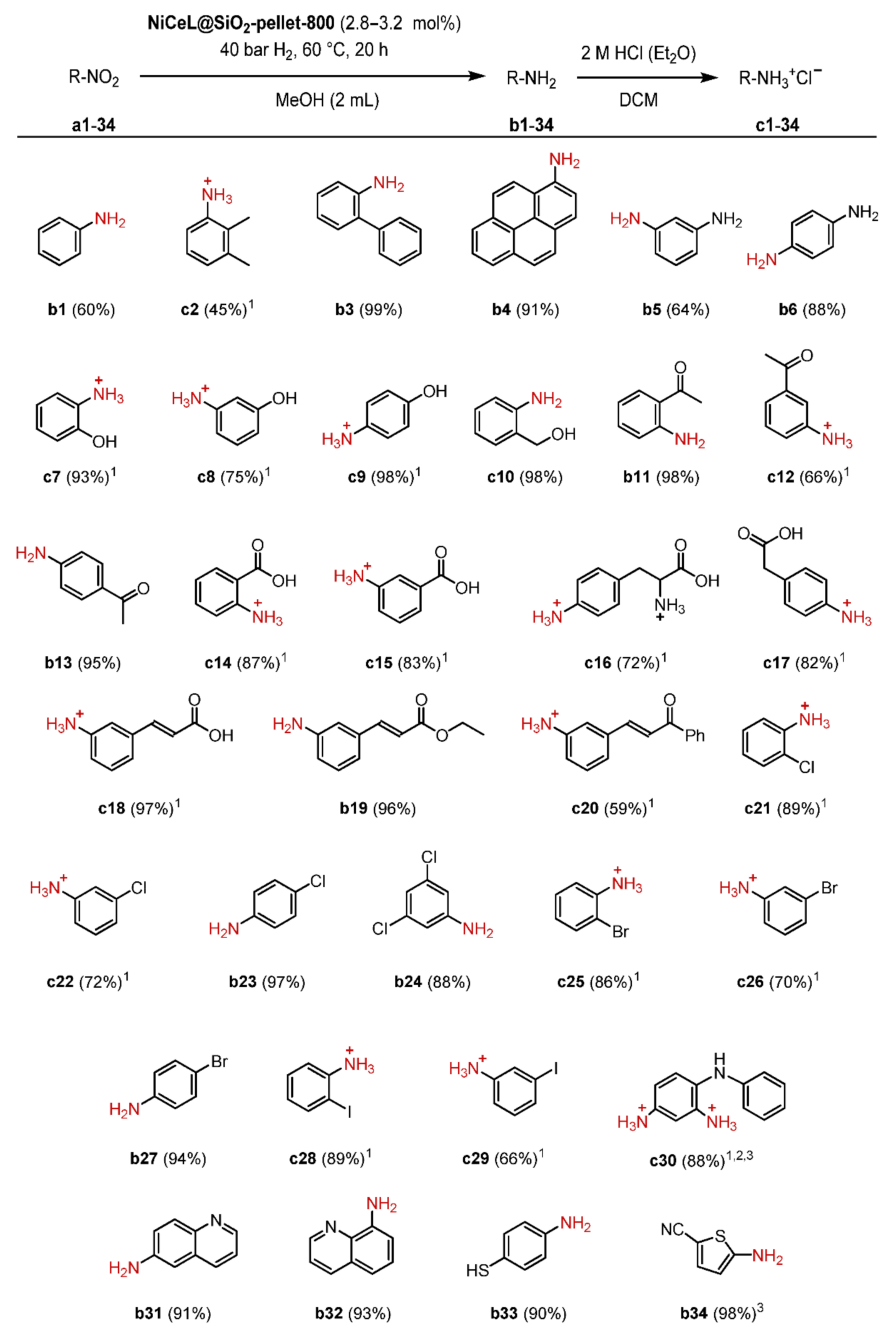
Product range that was established in the course of the catalytic hydrogenation of various aromatic nitro compounds by supported and pyrolyzed **[NiCeL]**. The values in parentheses indicate the yield of the obtained amine or ammonium salt, respectively. For reasons of better readability, the respective chloride counterions (Cl^−^) are not shown. ^1^ The immediate product was precipitated as the corresponding hydrochloride salt. ^2^ The catalyst loading was increased (5 mol%). ^3^ The reaction temperature was raised to 80 °C.

At the outset, we subjected parent nitrobenzene **a1** and the bis-methylated kindred **a2** to the standard reaction conditions (Scheme 3) whereupon the corresponding anilines were obtained in mediocre yields (60% **b1** and 45% **c2**, respectively). However, usage of the biphenyl derivative **a3** and the bulky, benzannulated nitro compound **a4** promptly enabled yields exceeding 90%.

Nitroarenes equipped with a pendant alcohol group (**a7**–**10**) readily formed the tagged phenols (**c7–9**) or the corresponding benzyl congener (**c10**). Only if OH was in the arene *meta* position, the product yield was considerably lower (75% **c8**). This trend was faithfully reproduced with substrates bearing N donors that are directly linked to the benzene core (**a5–6**, **a30**). Delightfully, nitroquinolines **a31–32**, which both naturally contain a well-coordinating sp^2^ nitrogen, also succumbed to the standard hydrogenation condition to smoothly yield the corresponding heterocyclic amines.

The chemoselectivity pertaining to the given hydrogenation method was initially demonstrated with the tagged acetophenones **a11–13** of which the *ortho* and *para* derivatives again gave rise to excellent results whereas the associated *meta* compound produced the organic amine **c12** only in a medium yield (66%). On application of *α,β*-unsaturated compounds **a18–20** as starting materials, the C=C bond remained intact, although the product formation was hampered in the case of chalcone **a20** (59% yield). Of note, the catalyst activity was not affected by the acidic COOH moiety of cinnamic acid **a18** and this tolerability of protons was further reassured by the successful conversion of the carboxylic acids **a16–17**.

In addition, the solid NiCe catalyst facilitated the hydrogenation of halonitroarenes **a21–29** without being unduly compromised by detrimental hydrodehalogenation; even substrates that contain the iodo substituent (**a28–29**), which notoriously undergoes this adverse side-reaction, proved amenable to the catalytic procedure described herein. However, as expected (vide supra), the reduction of the respective *m*-nitro-halobenzene with gaseous H_2_ resulted in rather low yields of the desired 3-haloanilines.

To our surprise, the given catalytic protocol was reconciled with the presence of a thiol functionality (**a33**), which is a quite remarkable result, especially because S atoms have the ability to poison catalytically active metal centers. Strikingly, our bimetallic pellet catalyst also coped with the simultaneous presence of an S-containing thienyl group and a reducible CN motif (**a34**) where the target amine was isolated in almost quantitative yield. Admittedly, to reach this beneficial result, the reaction temperature had to be increased to 80 °C.

**Scheme 4:**
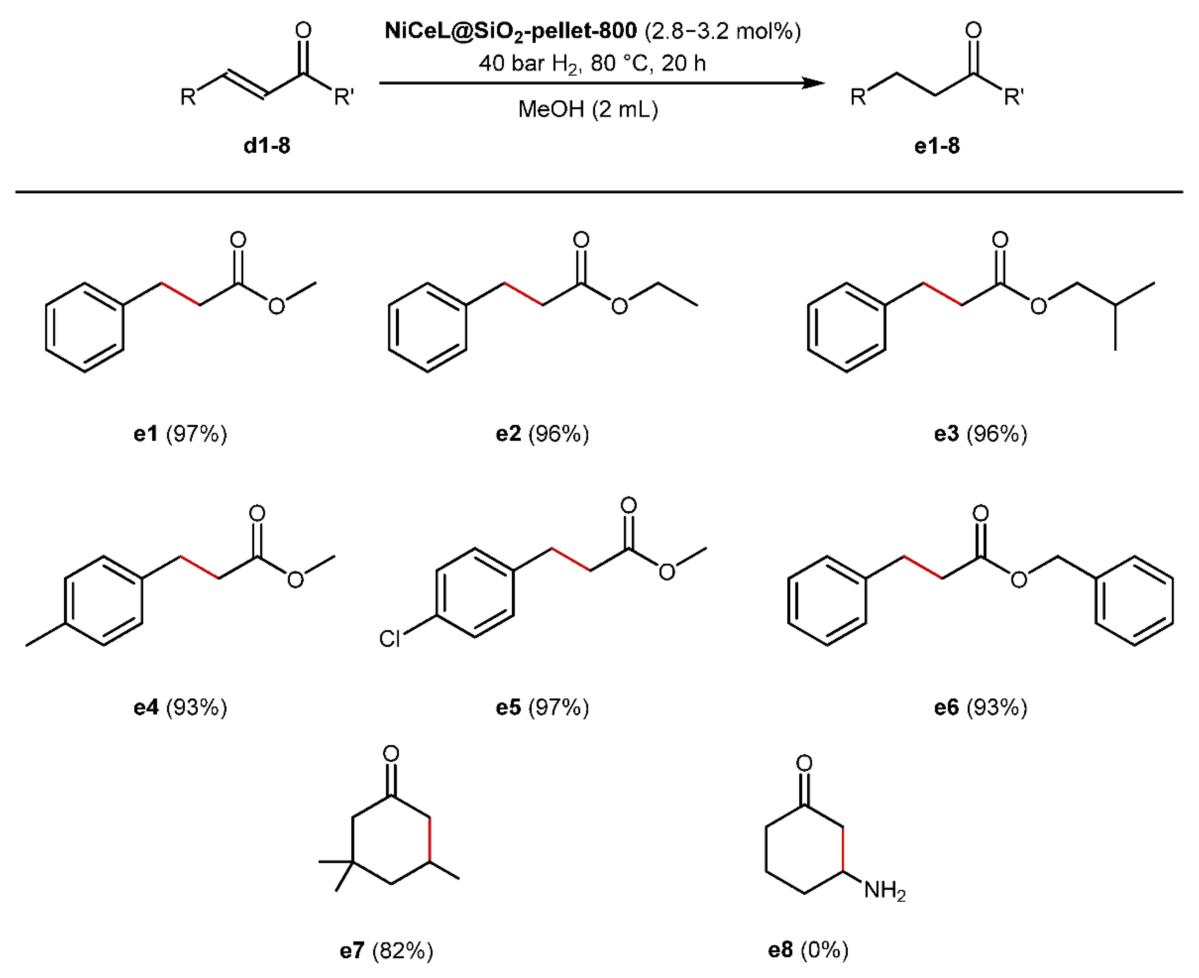
Conjugate hydrogenation (Michael reduction) of various cinnamic esters **d1**-**6** as well as cyclohexenones **d7** and **d8**. The pertinent catalytic transformations proceeded smoothly whereas the presence of the amino group in **d8** totally prevented the formation of the desired saturated ketone **e8**.

To further look into the reactivity towards C=C bonds, various cinnamic acid esters without a nitro functionality (**d1**-**6**) were hydrogenated in the presence of **NiCeL@SiO_2_-pellet-800** (Scheme 4). To our delight, all substrates were cleanly converted into the corresponding saturated derivatives, leaving the ester group untouched. Additionally, the described heterogeneous hydrogenation also worked out well with isophorone **d7** whereas the attachment of an amino group directly to the C-C double bond proved to be deleterious for the conjugate hydrogenation since we did not detect any tagged cyclohexanone **e8**. Of note, the chemoselective reduction of *α,β*-unsaturated carbonyl compounds using gaseous H_2_ is usually the realm of copper catalysis [124,125], whereas only recently a manganese-based system was described [126].

The NMR (^1^H and ^13^C) as well as the HR-MS spectra can be found in the Supplementary Materials section (Figures S9–S127).

### 2.6. Reusability Tests

For the assessment of the recyclability, 4-nitroacetophenone was subjected to the optimized reaction conditions (vide supra) whereby six consecutive runs were performed with two and the same pellets of the standard **NiCeL@SiO_2_-pellet-800** catalyst. After each iteration, the recovered catalyst chunk was sonicated in pure methanol for a period of 1 min. upon which the pellet was reused in the next run. Following this procedure, the catalyst performance remained, strikingly, unaffected throughout this series and in neither case did we observe any (unwanted) reduction of the ketone motif (Table 4). However, to guarantee an attrition-free course of the catalytic transformation, the stirrer speed had to be adjusted to a rather modest value of 500 rpm.

An additional benefit of the used composite NiCe catalyst comes from its compact form and dimension stability that allow for simple removal of the pellet from the reaction mixture with the aid of a conventional pair of tweezers (Figure S128).

## 3. Experimental Procedures

All chemicals were obtained through commercial suppliers (Merck, Fluorochem, Acros Organics, Alfa Aesar, BLDPharm, VWR, Roth, TCI, and Chem Lab) and used without further manipulations. The hydrogenation reactions were conducted in Parr^®^ autoclaves (300 mL) that were pressurized with H_2_ (5.0 purity, Linde Gas GmbH). Routine GC-MS analyses were carried out on a Shimadzu GC-MS QP-2020 (helium, 5.0 purity, Linde Gas GmbH) whereas HR-MS measurements were performed on an Agilent QTOF 6520. The collection of the NMR data was executed on Bruker Avance III spectrometers (300 MHz, 500 MHz) while the applied spectrometer frequencies of the various nuclei amounted to 300 MHz (^1^H NMR) and 75.5 MHz (^13^C{^1^H} NMR) on the 300 MHz machine and 470.5 MHz (^19^F NMR) was used on the 500 MHz spectrometer; the chemical shifts δ are listed in ppm and axis calibration based on the signal of residual nondeuterated solvent. The XPS data were acquired on a VG ESCALAB220iXL instrument (Thermo Scientific Inc., Waltham, MA, USA, 1486.68 eV Al K_α_ radiation) and scanning transmission electron microscopy (STEM) micrographs were taken on a probe aberration-corrected JEM-ARM200F electron microscope (JEOL, Tokio, Japan, CEOS corrector) equipped with a JED-2300 (JEOL) energy-dispersive X-ray spectrometer having a silicon drift detector (dry SD60GV). A high-angle annular dark field (HAADF) and an annular bright field (ABF) detector were used for general imaging. The solid samples were deposited without any pretreatment on a porous carbon-supported copper grid (mesh 300) and then passed to the microscope. Routine CHN analyses were conducted on a Leco Microanalysator TruSpec machine while the metal concentrations were determined via Atomic Absorption Spectroscopy using a PerkinElmer AAS Analyst 300 device. Finally, the TGA curves were recorded on a Pyris Series TGA4000 thermogravimetric analyzer.

### 3.1. Procedure for the Pyrolytic Synthesis of the Supported [NiCeL]-Based Heterogeneous Catalyst

At first, the precursor **[NiCeL]** (384 mg, 0.51 mmol) was solubilized in EtOH (30 mL) upon which the solid support (3.05 g of powdered CeO_2_, SiO_2_, Al_2_O_3_, Vulcan^®^ XC 72 R, or pelletized SiO_2_, respectively) was added portionwise within 30 min. The formed suspension was then heated under a reflux condenser (6 h) whereupon the solvent was removed under reduced pressure. The dried **[NiCeL]**-support composite was hereafter carefully pyrolyzed in an Austromat^®^ 624 furnace at the required temperature (Ar atmosphere, 2 h). Eventual cooling to ambient temperature afforded the ready-to-use, solid catalyst that is referred to as **NiCeL@support-X** where X marks the applied pyrolysis temperature (°C).

### 3.2. General Procedure for the Catalytic Hydrogenation Reactions

The hydrogenations were carried out in glass vials (4 mL), each of which was charged with solid **NiCeL@SiO_2_-pellet-800** (approx. 2–3 mol%), NO_2_-tagged substrate (0.5 mmol), solvent (2 mL) as well as a magnetic stirring bar in that order and without any protection from air. Each reaction vessel was sealed with a septum cap which was then pierced and equipped with a steel cannula. Hereafter, the vials were placed in a drilled aluminum plate that was transferred into the autoclave whereupon the latter was flushed with H_2_ (3 × 40 bar) before being pressurized to the desired value. Afterwards, the autoclave was placed on a heating plate upon which the stirring rate (500 rpm) and the required temperature were adjusted. On completion of the catalytic transformation, the autoclave was put in an ice bath in order to quickly reach ambient temperature and then the vessel was slowly depressurized. The catalyst was removed with a tweezer and the reaction solution was filtered through a short plug of silica; evaporation of the solvent under reduced pressure finally afforded the product amine.

#### 3.2.1. Safety Statement Concerning the Use of Pressurized Hydrogenation Gas

The H_2_-filled steel cylinder (200 bar, 50 L) was placed and lashed in a safety storage cabinet equipped with a tapping unit whereby the bottle was wired to a control panel that allowed for fine-adjustment of the H_2_ pressure. The autoclave charging procedure was performed in a fume hood with an integrated sensor which was connected to a magnetic valve that interrupts the gas feed in case of any hydrogen leakage that might occur during the filling procedure. Moreover, optical and acoustic alerts are triggered whenever flammable (or toxic) gas is detected inside the hood.

#### 3.2.2. General Procedure for the Precipitation of the Organic Ammonium Salts

The respective ammonium hydrochlorides of the synthesized amines (Section 3.2) were, if desired, obtained by initial treatment of the crude product with commercial, dry HCl solution (2 mL, 2 M in Et_2_O). The resultant precipitate was filtered off, washed with dichloromethane (DCM) (3 × 0.5 mL), and eventually dried in vacuo.

## 4. Conclusions

A user-friendly, robust, and low-cost route for the synthesis of a wide array of organic amines from the corresponding NO_2_-tagged compounds was presented. The pertinent heterogeneous hydrogenation reactions were effected by a solid, bimetallic pellet catalyst that was readily accessible through annealing of an immobilized dinuclear NiCe complex that incorporated a salen-type chelator. The thus-obtained composite material facilitated the mild, chemoselective reduction of functionalized nitroarenes equipped with alcohol, amine, ketone, carboxylic acids, or heterocyclic motifs. Moreover, the introduced catalytic protocol turned out to be free from unpleasant dehydrohalogenation events when employing halonitroarene substrates. A further notable feature of the introduced hydrogenation protocol is the fact that it was reconciled with the presence of classic, S-containing catalyst poisons such as thiols and thienyl compounds.

In addition, cinnamic esters minus nitro groups proved to be amenable to conjugate hydrogenation to afford the saturated products with the COOR functionality still intact.

The pertinent pelletized, bimetallic catalyst was recyclable several times whereupon both substrate conversion and selectivity were unaltered upon multiple use.

Given the vast possible combinations of 3d metals and lanthanides, the six-donor-atom-containing salen-type ligand discussed herein is likely to open new prospects for the conceptualization of innovative functional materials that are likely to discover new reaction space.

## Figures and Tables

**Table 1:** Hydrogenation of nitrobenzene: variation of the supporting material. Reaction conditions: nitrobenzene (0.5 mmol), catalyst (1.5 mol%), H_2_ (40 bar), MeOH as solvent (2 mL), 60 °C, 20 h, *n*-hexadecane as an internal standard (18 mg).

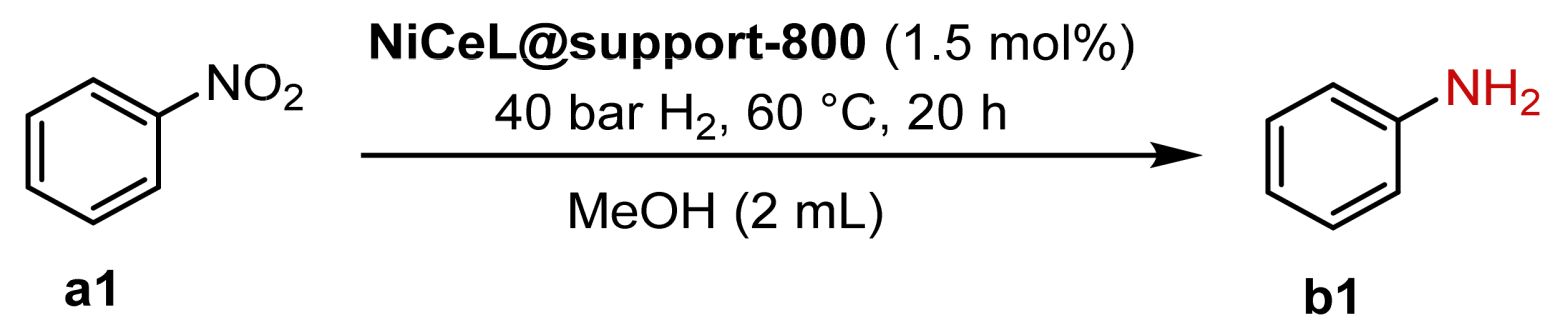			
Entry	Catalyst	Pyrolysis T (°C)	Conversion (%)
1	NiCeL@SiO_2_-pellet	800	33
2	NiCeL@SiO_2_	800	5
3	NiCeL@Al_2_O_3_	800	9
4	NiCeL@Vulcan	800	0
5	NiCeL@CeO_2_	800	0
6	[NiCeL]	800	0
7	[NiCeL]	no pyrolysis	0

**Table 2:** Hydrogenation of nitrobenzene: influence of the pyrolysis temperature on the catalyst performance. Reaction conditions: nitrobenzene (0.5 mmol), catalyst (2.5 mol%), H_2_ (40 bar), MeOH (2 mL), 60 °C, and 20 h.

Entry	Catalyst	Pyrolysis T (°C)	Isolated Yield (%)
1	NiCeL@SiO_2_-pellet	400	0
2	NiCeL@SiO_2_-pellet	600	0
3	NiCeL@SiO_2_-pellet	800	60
4	NiCeL@SiO_2_-pellet	900	7
5	NiCeL@SiO_2_-pellet	no pyrolysis	0

**Table 3:** Hydrogenation of nitrobenzene: effect of the reaction medium on the catalyst activity. Reaction conditions: nitrobenzene (0.5 mmol), pellet catalyst (2.5 mol%), H_2_ (40 bar), solvent (2 mL), 60 °C, and 20 h.

Entry	Solvent	Isolated Yield (%)
1	H_2_O	10
2	MeOH	60
3	EtOH	30
4	*i*-PrOH	0
5	pyridine	0
6	ACN	0
7	THF	0
8	diethylether	0
9	1,4-dioxane	0
10	CHCl_3_	0
11	DCM	9
12	EtOAc	0
13	toluene	10
14	*n*-heptane	45

**Table 4:** Recycling experiments: hydrogenation of 4-nitroacetophenone. Reaction conditions: 4-nitroacetophenone (0.5 mmol), catalyst (2.8 mol%), H_2_ (40 bar), MeOH (2 mL), 60 °C, and 20 h.

Entry	Run	Conversion (%)
1	1	>99
2	2	>99
3	3	>99
4	4	>99
5	5	>99
6	6	>99

## Data Availability

Not applicable.

## Supplementary Materials

The following supporting information can be downloaded at: https://www.mdpi.com/article/10.3390/ijms23158742/s1. References are cited in [109,127,128,129,130,131,132,133].
