# Supplementary material for: Mild and Efficient Heterogeneous Hydrogenation of Nitroarenes Facilitated by a Pyrolytically Activated Dinuclear Ni(II)-Ce(III) Diimine Complex
Source: Int J Mol Sci. 2022 Aug 5;23(15):8742. doi: 10.3390/ijms23158742 (PMC9369285; doi:10.3390/ijms23158742)
Supplement: Supplementary file 1 [file ijms-23-08742-s001.zip › ijms-1838514-supplementary.pdf]

Supporting Information

Mild and Efficient Heterogeneous Hydrogenation of Nitroarenes Facilitated by a Pyrolytically Activated Dinuclear Ni(II)-Ce(III) Diimine Complex

Jessica Michalke [1,2], Kirill Faust [1], Thomas Bögl [3], Stephan Bartling [4], Nils Rockstroh [4] and Christoph Topf [1,*]

1 Institute of Catalysis (INCA), Johannes Kepler University (JKU), Altenbergerstraße 69, 4040 Linz, Austria
2 Institute of Inorganic Chemistry, Johannes Kepler University (JKU), Altenbergerstraße 69, 4040 Linz, Austria
3 Department of Analytical Chemistry, Johannes Kepler University (JKU), Altenbergerstraße 69, 4040 Linz, Austria
4 Leibniz Institute for Catalysis at the University of Rostock (LIKAT Rostock), Albert-Einstein-Straße 29a, 18059 Rostock, Germany
* Correspondence: christoph.topf@jku.at

1. Effect of H_2 Pressure and Temperature on the Nitrobenzene Conversion Catalyzed by NiCeL@SiO_2-pellet-800......2

2. Conversion of Nitrobenzene Catalyzed by NiCeL@SiO_2-pellet-800 *vs.* NiL@SiO_2-pellet-800......2

3. Hydrogenation of Nitrobenzene; Control Experiments......3

4. Catalyst Characterization 3

4.1. Elemental Analyses (EA) for NiCeL@SiO_2-pellet-800......3

4.2. X-Ray Photoelectron Spectroscopy (XPS) and Energy Dispersive X-Ray Spectroscopy (EDX) Data of NiCeL@SiO_2-pellet-800......4

4.3. Thermogravimetric Analysis (TGA) of the [NiCeL] precursor......6

5. Synthesis and Characterization of [NiCeL]......7

5.1. Synthesis of [NiCeL]......7

5.2. Elemental Analysis (EA) of [NiCeL]......8

5.3. Crystallographic Data..8
6. Characterization Data of the Isolated Products..11
7. ^{1}H NMR, $^{13}C\{^{1}H\}$ NMR, and HR-MS Spectra of the Isolated Products..21
8. Pictorial Demonstration of Simple Catalyst Separation..80
9. References..81

1. Effect of H_2 Pressure and Temperature on the Nitrobenzene Conversion Catalyzed by NiCeL@SiO_2-pellet-800

Table S1. Hydrogenation of nitrobenzene: variation of temperature and pressure. Reaction conditions: nitrobenzene (0.5 mmol), catalyst (2.5 mol%), MeOH (2 mL), reaction time as indicated, *n*-hexadecane applied as internal standard (18 mg).

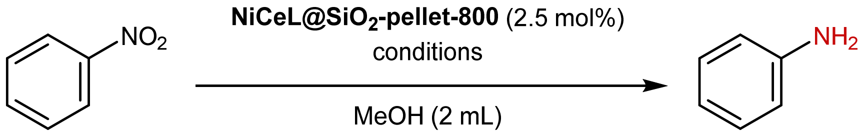

a1 b1

Entry	Catalyst	H_2 (bar)	T (°C)	Reaction time (h)	Conversion (%)
1	NiCeL@SiO_2-pellet-800	20	80	16	91
2	NiCeL@SiO_2-pellet-800	40	80	16	>99
3	NiCeL@SiO_2-pellet-800	60	60	16	76
4	NiCeL@SiO_2-pellet-800	20	60	16	64
5	NiCeL@SiO_2-pellet-800	40	60	20	>99
6	NiCeL@SiO_2-pellet-800	40	40	16	17

2. Conversion of Nitrobenzene Catalyzed by NiCeL@SiO2-pellet-800 *vs.* NiL@SiO2-pellet-800

Table S2. Hydrogenation of nitrobenzene: variation of the catalyst. Reaction conditions: nitrobenzene (0.5 mmol), catalyst (2.5 mol%), MeOH (2 mL), 40 bar H_2, 60 °C, 20 h, *n*-hexadecane applied as internal standard (18 mg).

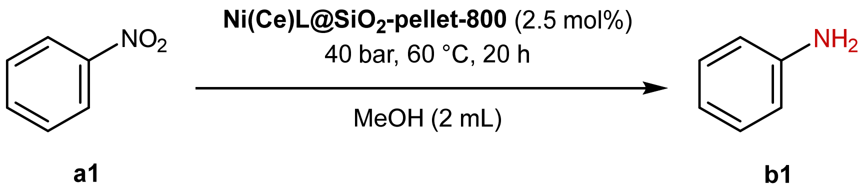

Entry	Catalyst	H_2 (bar)	T (°C)	Reaction time (h)	Conversion (%)
1	NiCeL@SiO2-pellet-800	20	80	16	91
2	NiL@SiO2-pellet-800	20	80	16	27
3	NiCeL@SiO2-pellet-800	40	80	16	>99
4	NiL@SiO2-pellet-800	40	80	16	>99
5	NiCeL@SiO2-pellet-800	40	40	16	17
6	NiL@SiO2-pellet-800	40	40	16	0

3. Hydrogenation of Nitrobenzene; Control Experiments

Table S3. Hydrogenation of nitrobenzene; variation of the catalyst, additive respectively. Reaction conditions: nitrobenzene (0.5 mmol), catalyst (1 mol%), MeOH (2 mL), 40 bar H_2, 60 °C, 20 h, *n*-hexadecane applied as internal standards (18 mg).

Entry	Catalyst	Pyrolysis T (°C)	Additive	Conversion (%)
1	SiO2-pellet	non-pyrolyzed	-	0
2	SiO2-pellet	800	-	0
3	-	-	-	0
4	NiCeL@SiO2-pellet	non-pyrolyzed	-	0

5	[NiCeL]	non-pyrolyzed	-	0
6	[NiCeL]	800	-	0
7	NiL@SiO_2-pellet	800	2.5 mol% $Ce(NO_3)_3 \cdot 6\ H_2O$	48
8	NiL@SiO_2-pellet	800	100 mol% $Ce(NO_3)_3 \cdot 6\ H_2O$	19

4. Catalyst Characterization

The used SiO_2 pellets were obtained from Alfa Aesar (silicon dioxide, catalyst support, high surface area, 3.18 mm average height), the mass of one pellet ranged from 31-46 mg.

4.1. Elemental Analyses (EA) for NiCeL@SiO_2-pellet-800

Table S4. EA (% by mass) obtained for **NiCeL@SiO_2-Pellet-800**.

**Entry**	**Ni (%)**	**Ce (%)**	**C (%)**	**H (%)**	**N (%)**
1	1.05	3.00	1.501	0.2313	0.0204
2	0.993	3.00	1.027	0.3187	0.0379

4.2. X-Ray Photoelectron Spectroscopy (XPS) and Energy Dispersive X-Ray Spectroscopy (EDX) Data of NiCeL@SiO_2-pellet-800

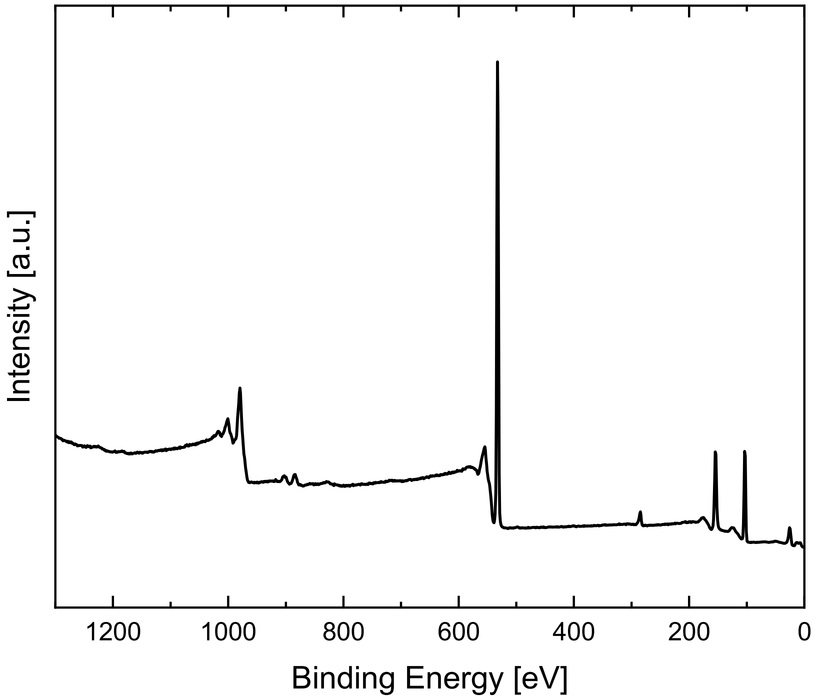

Figure S1. Total X-ray photoelectron spectra of **NiCeL@SiO_2-pellet-800**.

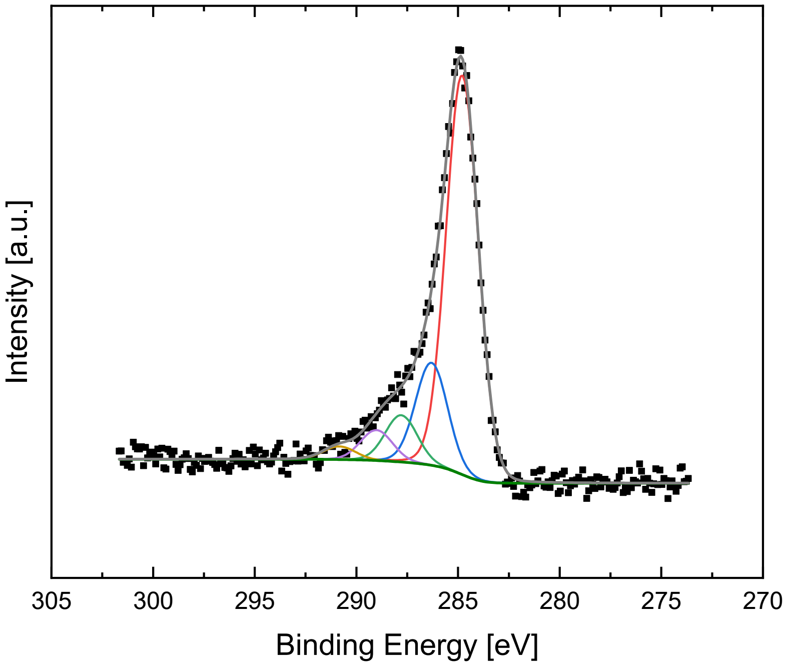

Figure S2. XPS C 1s spectrum of **NiCeL@SiO_2-pellet-800**.

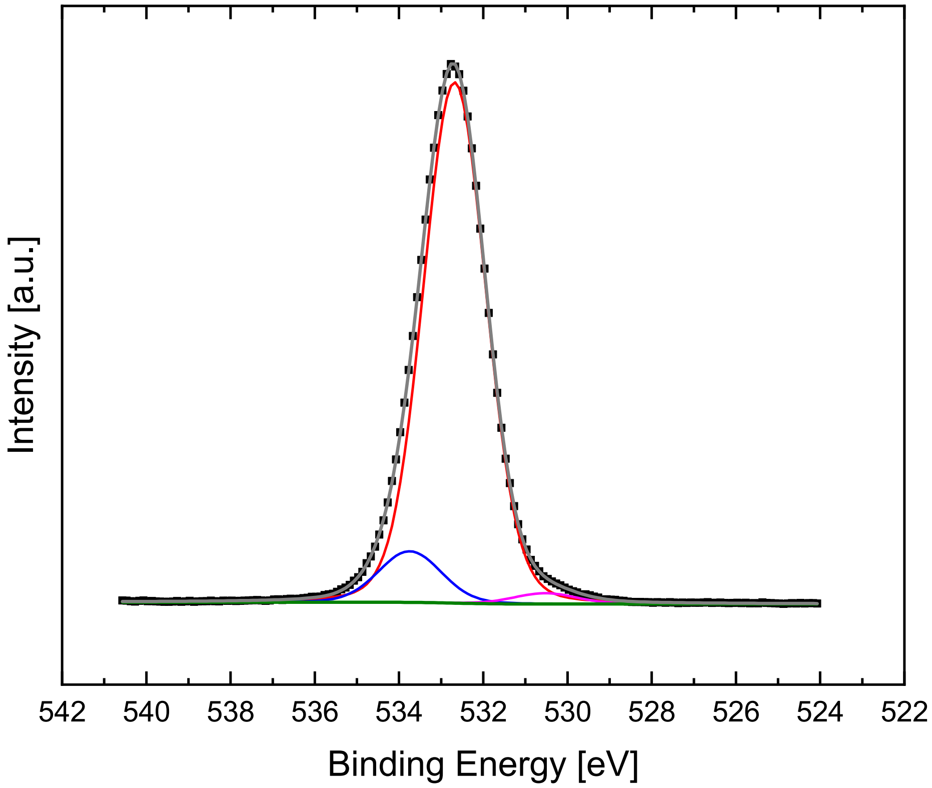

Figure S3. XPS O 1s spectrum of **NiCeL@SiO₂-pellet-800**.

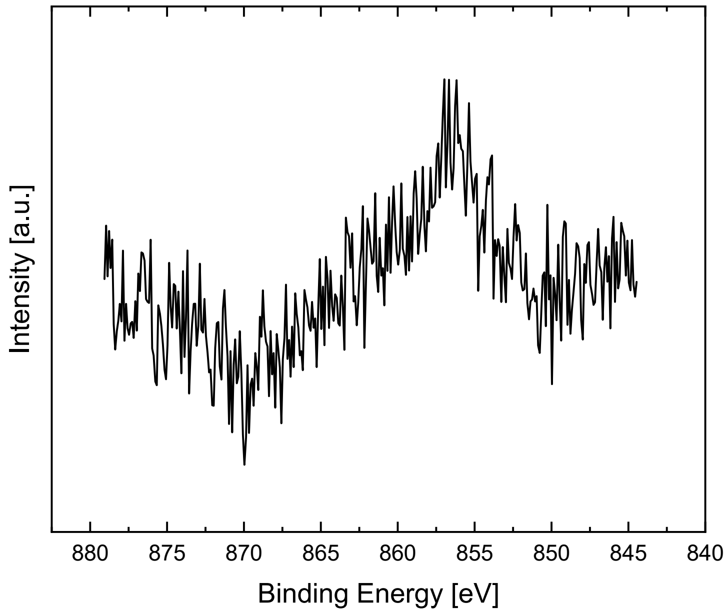

Figure S4. XPS Ni 2p region of **NiCeL@SiO₂-pellet-800**.

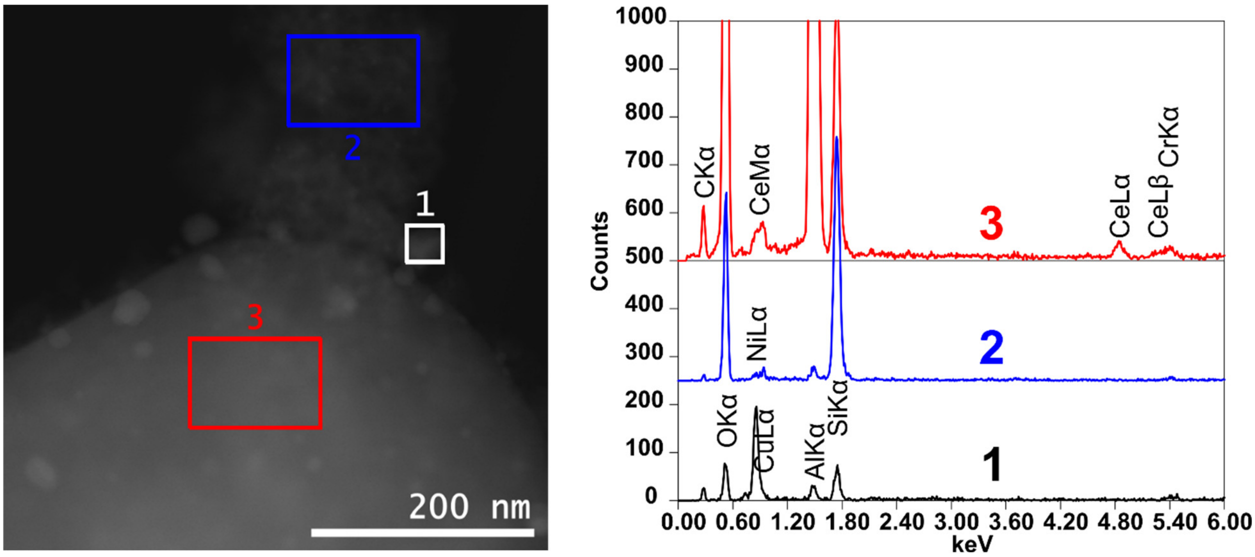

Figure S5. Selected EDX spectra (right) of the marked areas in the STEM-HAADF image (left) of **NiCeL@SiO$_2$-pellet-800**. While area 1 comprises a considerable amount of Ni, only little Ni is found in areas 2 and 3. The material contains a lot of Al in the region of area 3, which can be most likely traced back to the sample handling with alumina-coated tools. Moreover, area 3 was the only part of the sample, where Ce was found with STEM-EDX.

4.3. Thermogravimetric Analysis (TGA) of the [NiCeL] precursor

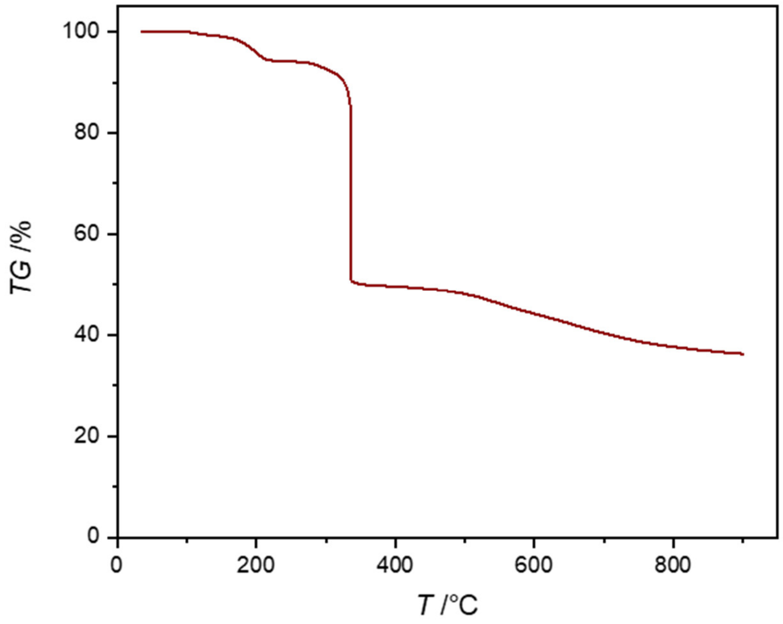

Figure S6. TGA trace of free **[NiCeL]** with thermal, molecular disintegration completed just before 800 °C which is indicated by the slight bend in the curve.

5. Synthesis and Characterization of [NiCeL]

5.1. Synthesis of [NiCeL]

The complex was prepared in analogy to a published procedure [1], with the exception that, instead of a Cu source, a Ni salt was employed. All steps were carried out at ambient temperature with any protection from air. A mixture of 1,3-diamino-2,2′-dimethylpropane (52 mg, 0.51 mmol) and *o*-vanillin (168 mg, 1.10 mmol) was dissolved in MeOH (30 mL), to produce a yellow solution that was stirred for 30 min. Then, solid $Ni(OAc)_2 \cdot 4\ H_2O$ (177 mg, 0.71 mmol) was added upon which stirring was continued for 1 h. Hereafter, the reaction solution adopted a brown color that turned into green after the addition of $Ce(NO_3)_3 \cdot 6\ H_2O$ (308 mg, 0.71 mmol). After the solution had been agitated for a further 15 h, the liquid reaction mixture was reduced to a small volume whereupon Et_2O was added to precipitate the crude which was collected on a frit and washed with Et_2O (3×5 mL) to afford a green-blue solid. As the yield exceeded 100% on drying the compound in a desiccator, the complex is likely to incorporate two additional, axial H_2O ligands as can be seen in the crystal structure, that were incorporated from residues of crystal water from the precursors. Appearance: blue-green solid, 380 g (0.481 mmol, 95 %). Analytical data: HR-MS (ESI-Orbitrap) *m/z*: $[M\text{-}NO_3]^+$ calcd. for $C_{21}H_{24}CeN_5NiO_{13}$: 689.9900; 689.9906. Crystals of X-ray quality were obtained in the fridge through slow diffusion of Et_2O into a solution of **NiCeL** in acetone. This caused the title compound to deposit as light-blue crystals on the glass wall within one week.

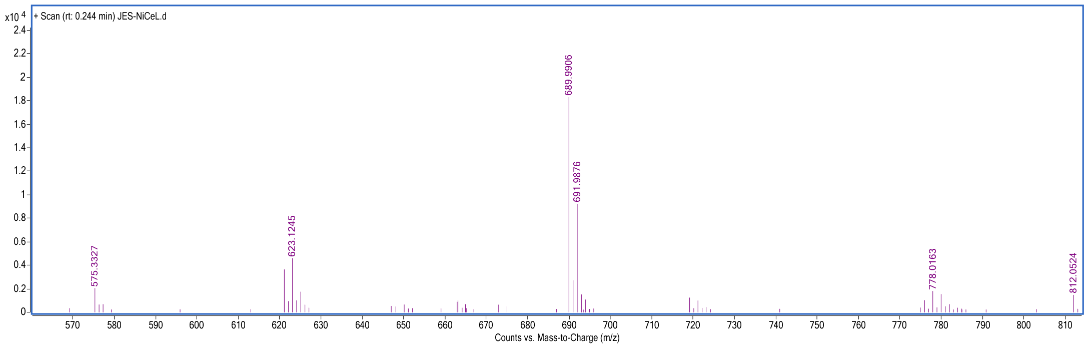

Figure S7. HR-MS of **[NiCeL]**.

5.2. Elemental Analysis (EA) of [NiCeL]

Table S5. EA (% by mass for the solution phase precursor complex **[NiCeL]**.

Entry	Ni (%)	Ce (%)	C (%)	H (%)	N (%)
1	7.45	18.95	25.53	3.298	7.835
2	7.44	19.08	25.50	3.306	8.027

5.3. Crystallographic Data

X-ray quality crystals were selected in Fomblin® Y H-VAC 140/13 perfluoropolyether at ambient temperature. The data was collected at 296(2) K on a *Bruker D8 Quest Eco* diffractometer using graphite monochromated Mo K_α radiation (λ = 0.71073 Å). The data was processed using APEX3 [1], the structures were solved by intrinsic phasing (XT, Version 2018/2) [2], and refined by full matrix least squares procedures on F^2 (SHELXL, Version 2018/3) [3] using the graphical interface Shelxle [4] within the SHELXTL suite of programs by Bruker. All non-hydrogen atoms were refined anisotropically. All hydrogen atoms were calculated geometrically, and a riding model was applied in the refinement process.

The hydrogen atoms at water ligand oxygens O5 and O6 could not be found and were not added in the refinement process.

CCDC 2181155 contains the supplementary crystallographic data for compound **NiCeL**. These data can be obtained free of charge from The Cambridge Crystallographic Data Center at www.ccdc.cam.ac.uk.

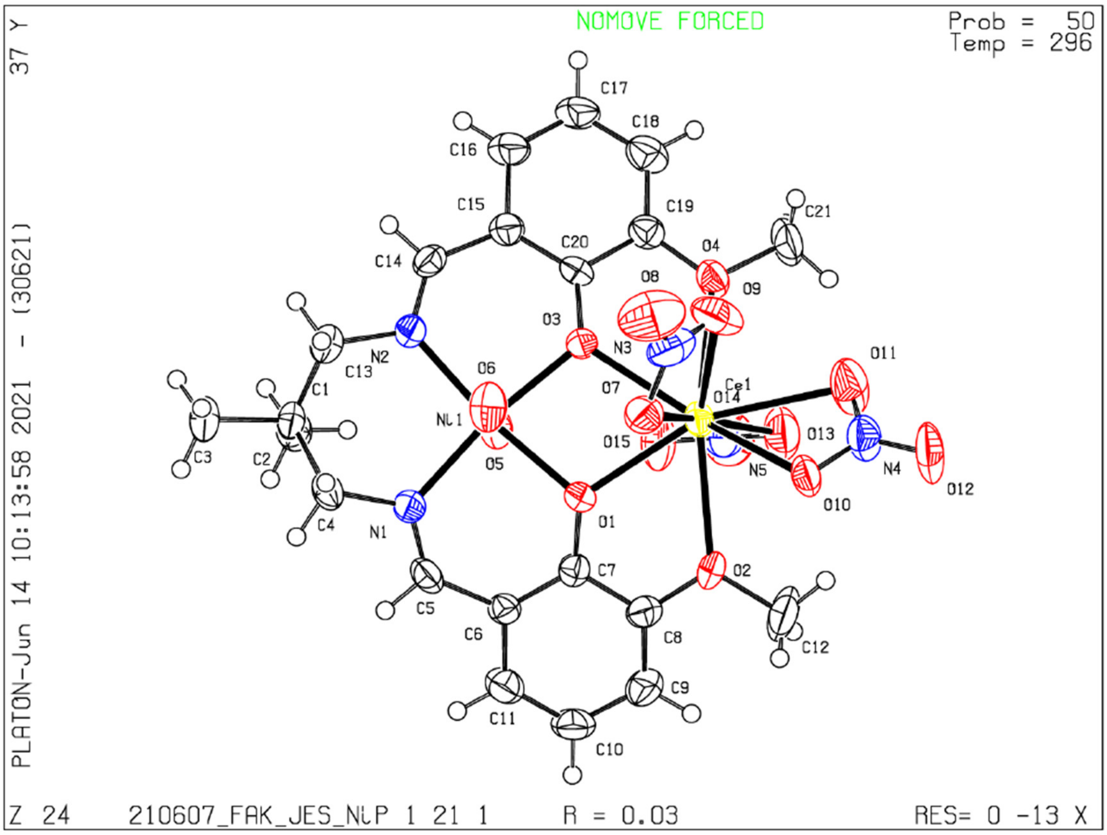

Figure S8. Representation of the molecular structure of **[NiCeL]** as obtained by single crystal X-ray diffraction analysis; the atomic numbering is included.

Table S6. Crystal data, data collection and structure refinement details for compound **[NiCeL]**.

Compound	**[NiCeL]**
Empirical formula	$C_{21}H_{24}Ce_1Ni_1N_5O_{15}$
Formula weight [g/mol]	785.28
Color	light blue
Crystal size [mm]	0.23 × 0.21 × 0.19
Crystal system	monoclinic
Space group	$P2_1$
a [Å]	8.9758(3)
b [Å]	16.2125(6)
c [Å]	10.1002(4)
α [°]	90
β [°]	91.244(1)
γ [°]	90
V [Å^3]	1469.44(9)
Z	2
D_{calc} [g/cm^3]	1.775
μ [mm^{-1}]	2.25
T [K]	296
θ range [°]	2.4-16.5
No. of reflections measured	30317
No. of independent reflections	5359
Obs. Reflections with $I > 2\sigma(I)$	4495
No. of Parameters refined/restraints	392/1
Absorption correction	multi-scan
T_{min}, T_{max}	0.62, 0.68
$\Delta\varrho_{min}/\Delta\varrho_{max}$ [e Å^{-3}]	−0.58/0.39
F(000)	782
R_{int}	0.053
R_1 ($R[F^2 \geq 2\sigma(F^{2)})]$)	0.031

wR_2 ($wR(F^2)$)	0.059
GooF	1.03
CCDC no.	2181155

6. Characterization Data of the Isolated Products

General note: The catalyst loading was calculated using the bulk Ni content of 1.05 %.

*Aniline (**b1**).* The title compound was synthesized according to the standard procedure. 64.0 mg (0.520 mmol) of **a1** was used, 2.8 mol% catalyst. Appearance: colorless oil, 29.0 mg (0.311 mmol, 60 % yield). Analytical data: ^{1}H NMR (300 MHz, $CDCl_3$, 25 °C): δ = 7.18 – 7.13 (m, 2H), 6.76 – 6.68 (m, 3H), 3.64 (br, 2H) ppm; ^{13}C{^{1}H} NMR (75.5 MHz, $CDCl_3$, 25 °C): δ = 146.3, 129.3, 118.5, 115.1 ppm.

*2,3-Dimethylbenzenaminium chloride (**c2**).* The title compound was synthesized according to the standard procedure including the precipitation step. 58.6 mg (0.509 mmol) of **a2** was used, 3.0 mol% catalyst. Appearance: red-brown powder, 36.4 mg (0.231 mmol, 45 % yield). Analytical data: ^{1}H NMR (300 MHz, D_2O, 25 °C): δ = 7.33 – 7.26 (m, 1H), 7.23 – 7.18 (m, 2H), 2.31 (s, 3H), 2.24 (s, 3H) ppm; ^{13}C{^{1}H} NMR (75.5 MHz, D_2O, 25 °C): δ = 139.7, 130.4, 130.4, 128.5, 126.9, 120.6, 19.2, 12.7 ppm; HR-MS (ESI-Orbitrap) *m/z*: $[M-Cl]^+$ calcd. for $C_8H_{12}N$: 122.0964; found: 122.0962.

*2-Aminobiphenyl (**b3**).* The title compound was synthesized according to the standard procedure. 98.7 mg (0.500 mmol) of **a3** was used, 3.0 mol% catalyst. Appearance: silver-grey powder, 83.0 mg (0.491 mmol, 99 % yield). Analytical data: ^{1}H NMR (300 MHz, $CDCl_3$, 25 °C): δ = 7.63 – 7.60 (m, 2H), 7.50 – 7.43 (m, 2H), 7.40 – 7.35 (m, 1H), 7.30 – 7.24 (m, 1H), 7.06 – 7.03 (m, 1H), 6.96 – 6.93 (m, 1H), 6.72 – 6.70 (m, 1H), 3.76 (br, 2H) ppm; ^{13}C{^{1}H} NMR

(75.5 MHz, $CDCl_3$, 25 °C): δ = 146.7, 142.4, 141.3, 129.6, 128.6, 127.1, 127.0, 117.6, 114.0, 113.8 ppm; HR-MS (ESI-Orbitrap) *m/z*: $[M+H]^+$ calcd. for $C_{12}H_{12}N$: 170.0964; found: 170.0965.

*1-Aminopyrene (**b4**).* The title compound was synthesized according to the standard procedure. 123.4 mg (0.499 mmol) of **a4** was used, 3.5 mol% catalyst. Appearance: gold solid, 100.3 mg (0.457 mmol, 98 % yield). Analytical data: ^1H NMR (300 MHz, $CDCl_3$, 25 °C): δ = 8.09 – 7.93 (m, 7H), 7.84 (d, J = 8.7 Hz, 1H), 7.39 (d, J = 8.7 Hz, 1H), 4.50 (br, 2H) ppm; $^{13}C\{^1H\}$ NMR (75.5 MHz, $CDCl_3$, 25 °C): δ = 140.9, 132.2, 131.7, 127.6, 126.1, 126.0, 125.5, 124.3, 124.1, 123.8, 123.6, 120.2, 116.9, 114.0 ppm.

*1,3-Diaminobenzene (**b5**).* The title compound was synthesized according to the standard procedure. 69.2 mg (0.501 mmol) of **a5** was used, 3.1 mol% catalyst. Appearance: brown solid, 34.5 mg (0.319 mmol, 64 % yield). Analytical data: ^1H NMR (300 MHz, $CDCl_3$, 25 °C): δ = 6.97 – 6.92 (m, 1H), 6.14 – 6.11 (m, 2H), 6.11 – 6.02 (m, 1H), 3.57 (br, 4H) ppm; $^{13}C\{^1H\}$ NMR (75.5 MHz, $CDCl_3$, 25 °C): δ = 147.4, 130.1, 105.9, 101.8 ppm; HR-MS (ESI-Orbitrap) *m/z*: $[M+H]^+$ calcd. for $C_6H_9N_2$: 109.0760; found: 109.0762.

*1,4-Diaminobenzene (**b6**).* The title compound was synthesized according to the standard procedure. 69.8 mg (0.505 mmol) of **a6** was used, 3.2 mol% catalyst. Appearance: red powder, 48.1 mg (0.445 mmol, 88 % yield). Analytical data: ^1H NMR (300 MHz, $CDCl_3$, 25 °C): δ = 6.57 (s, 4H), 3.32 (br, 2H) ppm; $^{13}C\{^1H\}$ NMR (75.5 MHz, $CDCl_3$, 25 °C): δ = 138.7, 116.83 ppm; HR-MS (ESI-Orbitrap) *m/z*: $[M+H]^+$ calcd. for $C_6H_9N_2$: 109.0760; found: 109.0762.

*2-Hydroxybenzenaminium chloride (**c7**).* The title compound was synthesized according to the standard procedure including the precipitation step. 69.7 mg (0.501 mmol) of **a7** was used, 2.9 mol% catalyst. Appearance: red-brown powder, 68.2 mg (0.469 mmol, 94 % yield).

Analytical data: ^{1}H NMR (300 MHz, D_2O, 25 °C): δ = 7.33 – 7.28 (m, 2H), 7.04 – 6.95 (m, 2H) ppm; $^{13}C\{^{1}H\}$ NMR (75.5 MHz, D_2O, 25 °C): δ = 157.7, 149.5, 129.8, 123.6, 120.6, 116.3 ppm; HR-MS (ESI-Orbitrap) *m/z*: $[M-Cl]^+$ calcd. for C_6H_8NO: 110.0600; found: 110.0602.

*3-Hydroxybenzenaminium chloride (**c8**)*. The title compound was synthesized according to the standard procedure including the precipitation step. 69.5 mg (0.500 mmol) of **a8** was used, 3.1 mol% catalyst. Appearance: olive powder, 54.5 mg (0.374 mmol, 75 % yield). Analytical data: ^{1}H NMR (300 MHz, D_2O, 25 °C): δ = 7.39 – 7.34 (m, 1H), 6.97 – 6.89 (m, 2H), 6.86 – 6.84 (m, 1H) ppm; $^{13}C\{^{1}H\}$ NMR (75.5 MHz, D_2O, 25 °C): δ = 156.8, 131.2, 131.1, 116.0, 114.5, 110.0 ppm; HR-MS (ESI-Orbitrap) *m/z*: $[M-Cl]^+$ calcd. for C_6H_8NO: 110.0600; found: 110.0603.

*4-Hydroxybenzenaminium chloride (**c9**)*. The title compound was synthesized according to the standard procedure including the precipitation step. 69.4 mg (0.499 mmol) of **a9** was used, 2.8 mol% catalyst. Appearance: dark-violet powder, 68.0 mg (0.467 mmol, 94 % yield). Analytical data: ^{1}H NMR (300 MHz, D_2O, 25 °C): δ = 7.22 (d, J = 8.8 Hz, 2H), 6.93 (d, J = 8.8 Hz, 2H) ppm; $^{13}C\{^{1}H\}$ NMR (75.5 MHz, D_2O, 25 °C): δ = 155.7, 124.0, 122.7, 116.5 ppm; HR-MS (ESI-Orbitrap) *m/z*: $[M-Cl]^+$ calcd. for C_6H_8NO: 110.0600; found: 110.604.

*2-Aminobenzyl alcohol (**b10**)*. The title compound was synthesized according to the standard procedure. 76.2 mg (0.498 mmol) of **a10** was used, 2.9 mol% catalyst. Appearance: white powder, 60.0 mg (0.487 mmol, 98 % yield). Analytical data: ^{1}H NMR (300 MHz, $CDCl_3$, 25 °C): δ = 7.17 – 7.05 (m, 2H), 6.75 – 6.69 (m, 2H), 4.66 (s, 2H) ppm; $^{13}C\{^{1}H\}$ NMR (75.5 MHz, $CDCl_3$, 25 °C): δ = 146.1, 129.4, 129.2, 124.8, 118.2, 116.0, 64.4 ppm; HR-MS (ESI-Orbitrap) *m/z*: $[M+H]^+$ calcd. for $C_7H_{10}NO$: 124.0757; found: 124.0756.

*2-Aminoacetophenone (**b11**)*. The title compound was synthesized according to the standard procedure. 82.0 mg (0.497 mmol) of **a11** was used, 3.2 mol% catalyst. Appearance: white crystalline powder, 65.4 mg (0.483 mmol, 98 % yield). Analytical data: ^{1}H NMR (300 MHz, $CDCl_3$, 25 °C): δ = 7.72 – 7.69 (m, 1H), 7.28 – 7.23 (m, 1H), 6.66 – 6.61 (m, 2H), 6.62 (br, 2H), 2.57 (s, 3H) ppm; $^{13}C\{^1H\}$ NMR (75.5 MHz, $CDCl_3$, 25 °C): δ = 150.2, 134.3, 131.9, 118.1, 117.1, 115.6, 27.7 ppm; HR-MS (ESI-Orbitrap) *m*/*z*: $[M-H]^+$ calcd. for $C_8H_{10}NO$: 136.0757; found: 136.0758.

*3-Acetylbenzenaminium chloride (**c12**)*. The title compound was synthesized according to the standard procedure including the precipitation step. 82.7 mg (0.501 mmol) of **a12** was used, 3.0 mol% catalyst. Appearance: brownish powder, 56.3 mg (0.328 mmol, 66% yield). Analytical data: ^{1}H NMR (300 MHz, D_2O, 25 °C): δ = 8.11 – 8.08 (m, 1H), 7.94 (s, 1H), 7.70 – 7.68 (m, 2H), 2.68 (s, 3H) ppm; $^{13}C\{^1H\}$ NMR (75.5 MHz, D_2O, 25 °C): δ = 202.0, 138.1, 130.7, 129.2, 127.8, 122.3, 26.3 ppm; HR-MS (ESI-Orbitrap) *m*/*z*: $[M-Cl]^+$ calcd. for $C_8H_{10}NO$: 136.0757; found: 136.0757.

*4-Aminoacetophenone (**b13**)*. The title compound was synthesized according to the standard procedure. 82.8 mg (0.501 mmol) of **a13** was used, 3.1 mol% catalyst. Appearance: yellow powder, 64.5 mg (0.477 mmol, 95 % yield). Analytical data: ^{1}H NMR (300 MHz, $CDCl_3$, 25 °C): δ = 7.79 (d, J = 8.8 Hz, 2H), 6.63 (d, J = 8.8 Hz, 2H), 4.10 (br, 2H), 2.48 (s, 3H) ppm; $^{13}C\{^1H\}$ NMR (75.5 MHz, $CDCl_3$, 25 °C): δ = 196.5, 151.1, 130.8, 128.0, 113.7, 26.1 ppm; HR-MS (ESI-Orbitrap) *m*/*z*: $[M-H]^+$ calcd. for $C_8H_{10}NO$: 136.0757; found: 136.0759.

*2-Carboxybenzenaminium chloride (**c14**)*. The title compound was synthesized according to the standard procedure including the precipitation step. 83.6 mg (0.500 mmol) of **a14** was used, 3.3 mol% catalyst. Appearance: off-white powder, 75.9 mg (0.437 mmol, 87 % yield).

Analytical data: ^{1}H NMR (300 MHz, D_2O, 25 °C): δ = 8.17 – 8.14 (m, 1H), 7.76 – 7.70 (m, 1H), 7.58 – 7.53 (m, 1H), 7.48 – 7.46 (m, 1H) ppm; ^{13}C{^{1}H} NMR (75.5 MHz, D_2O, 25 °C): δ = 168.8, 134.7, 132.7, 132.3, 128.7, 124.2, 122.7 ppm; HR-MS (ESI-Orbitrap) *m*/*z*: [M-Cl]$^{+}$ calcd. for $C_7H_8NO_2$: 136.0550; found: 136.0551.

*3-Carboxybenzenaminium chloride (**c15**)*. The title compound was synthesized according to the standard procedure including the precipitation step. 83.5 mg (0.500 mmol) of **a15** was used, 3.0 mol% catalyst. Appearance: yellowish powder, 71.9 mg (0.415 mmol, 83 % yield). Analytical data: ^{1}H NMR (300 MHz, D_2O, 25 °C): δ = 7.95 – 7.84 (m, 1H), 7.84 (s, 1H), 7.55 – 7.52 (m, 2H) ppm; ^{13}C{^{1}H} NMR (75.5 MHz, D_2O, 25 °C): δ = 170.4, 133.5, 131.8, 130.3, 129.0, 126.4, 123.0 ppm; HR-MS (ESI-Orbitrap) *m*/*z*: [M-Cl]$^{+}$ calcd. for $C_7H_8NO_2$: 138.0550; found: 138.0550.

*4-(2-ammonio-2-carboxyethyl)benzenaminium chloride (**c16**)*. The title compound was synthesized according to the standard procedure including the precipitation step. 105.6 mg (0.502 mmol) of **a15** was used, 3.1 mol% catalyst. Appearance: yellowish powder, 91.2 mg (0.360 mmol, 72 % yield). Analytical data: ^{1}H NMR (300 MHz, D_2O, 25 °C): δ = 8.23 (d, J = 8.5 Hz, 1H), 7.53 (d, J = 8.6 Hz, 1H), 7.47 – 7.39 (m, 2H), 4.38 – 4.28 (m, 1H), 3.44 – 3.29 (m, 2H) ppm; ^{13}C{^{1}H} NMR (75.5 MHz, D_2O, 25 °C): δ = 171.3, 147.2, 142.3, 135.5, 131.1, 129.4, 124.2, 54.2, 35.2 ppm; HR-MS (ESI-Orbitrap) *m*/*z*: [M-Cl]$^{+}$ calcd. for $C_9H_{13}N_2O_2$: 181.0972; found: 181.0970.

*4-(carboxymethyl)benzenaminium chloride (**c17**)*. The title compound was synthesized according to the standard procedure including the precipitation step. 90.8 mg (0.501 mmol) of **a17** was used, 3.0 mol% catalyst . Appearance: yellow powder, 77.1 mg (0.411 mmol, 82 % yield). Analytical data: ^{1}H NMR (300 MHz, D_2O, 25 °C): δ = 7.44 – 7.35 (m, 4H), 3.75 (s, 2H) ppm;

$^{13}C\{^1H\}$ NMR (75.5 MHz, D_2O, 25 °C): δ = 176.9, 135.6, 131.0, 129.1, 123.0, 40.4 ppm; HR-MS (ESI-Orbitrap) *m/z*: $[M-Cl]^+$ calcd. for $C_8H_9NO_2$: 152.0706; found: 152.0709.

*(E)-3-(2-carboxyvinyl)benzenaminium chloride (**c18**).* The title compound was synthesized according to the standard procedure including the precipitation step. 96.8 mg (0.501 mmol) of **a18** was used, 3.5 mol% catalyst. Appearance: grey powder, 96.6 mg (0.484 mmol, 97 % yield). Analytical data: ^1H NMR (300 MHz, D_2O, 25 °C): δ = 7.75 – 7.70 (m, 2H), 7.62 – 7.56 (m, 2H), 7.47 – 7.44 (m, 1H), 6.62 – 6.56 (m, 1H) ppm; $^{13}C\{^1H\}$ NMR (75.5 MHz, D_2O, 25 °C): δ = 170.6, 144.2, 136.1, 130.9, 130.7, 128.6, 124.5, 122.3, 119.6 ppm; HR-MS (ESI-Orbitrap) *m/z*: $[M-Cl]^+$ calcd. for $C_9H_{10}NO_2$: 164.0706; found: 164.0706.

*Ethyl (E)-3-(3-aminophenyl)acrylate (**b19**).* The title compound was synthesized according to the standard procedure. 110.7 mg (0.500 mmol) of **a19** was used, 3.2 mol% catalyst. Appearance: yellow needles, 91.5 mg (0.479 mmol, 96 % yield). Analytical data: ^1H NMR (300 MHz, $CDCl_3$, 25 °C): δ = 7.62 (d, J = 15.6 Hz, 1H), 7.38 (d, J = 8.5 Hz, 2H), 6.68 (d, J = 8.5 Hz, 2H), 6.26 (d, J = 15.6 Hz, 1H), 4.30 – 4.23 (m, 2H), 3.94 (br, 2H), 3.51 (d, J = 5.6 Hz, 1H), 1.37 – 1.32 (m, 3H) ppm; $^{13}C\{^1H\}$ NMR (75.5 MHz, $CDCl_3$, 25 °C): δ = 167.8, 148.8, 144.9, 129.9, 124.6, 114.8, 113.6, 60.2, 14.4 ppm; HR-MS (ESI-Orbitrap) *m/z*: $[M+H]^+$ calcd. for $C_{11}H_{13}NO_2$: 192.1019; found: 192.1016.

*(E)-3-(3-oxo-3-phenylprop-1-en-1-yl)benzenaminium chloride (**c20**).* The title compound was synthesized according to the standard procedure including the precipitation step. 126.4 mg (0.499 mmol) of **a20** was used, 3.0 mol% catalyst. Appearance: dark-yellow powder, 76.1 mg (0.293 mmol, 59 % yield). Analytical data: ^1H NMR (300 MHz, D_2O, 25 °C): δ = 8.00 – 7.98 (m, 2H), 7.83 – 7.80 (m, 1H), 7.68 – 7.62 (m, 4H), 7.59 – 7.54 (m, 3H), 7.48 – 7.46 (m, 1H) ppm; $^{13}C\{^1H\}$ NMR (75.5 MHz, D_2O, 25 °C): δ = 194.6, 144.2, 136.9, 136.4, 134.0, 130.7, 130.5, 129.0, 128.9, 128.8,

124.9, 124.2, 122.9 ppm; HR-MS (ESI-Orbitrap) *m/z*: [M-Cl]$^+$ calcd. for $C_{15}H_{14}NO$: 224.1070; found: 224.1075.

*2-Chloroanilinium chloride (**c21**)*. The title compound was synthesized according to the standard procedure including the precipitation step. 78.7 mg (0.500 mmol) of **a21** was used, 3.1 mol% catalyst. Appearance: off-white powder, 72.7 mg (0.443 mmol, 89 % yield). Analytical data: ^{1}H NMR (300 MHz, D_2O, 25 °C): δ = 7.60 – 7.57 (m, 1H), 7.45 – 7.40 (m, 3H) ppm; ^{13}C{^{1}H} NMR (75.5 MHz, D_2O, 25 °C): δ = 130.5, 129.7, 128.8, 128.4, 127.0, 124.0 ppm; HR-MS (ESI-Orbitrap) *m/z*: [M-Cl]$^+$ calcd. for C_6H_7ClN: 128.0262; found: 128.0256.

*3-Chloroanilinium chloride (**c22**)*. The title compound was synthesized according to the standard procedure including the precipitation step. 78.3 mg (0.497 mmol) of **a22** was used, 3.1 mol% catalyst. Appearance: off-white powder, 58.5 mg (0.357 mmol, 72 % yield). Analytical data: ^{1}H NMR (300 MHz, D_2O, 25 °C): δ = 7.64 – 7.44 (m, 1H), 7.48 – 7.44 (m, 3H) ppm; ^{13}C{^{1}H} NMR (75.5 MHz, D_2O, 25 °C): δ = 131.3, 129.0, 123.0, 121.2 ppm; HR-MS (ESI-Orbitrap) *m/z*: [M-Cl]$^+$ calcd. for C_6H_7ClN: 128.0262; found: 128.0267.

*4-Chloroaniline (**b23**)*. The title compound was synthesized according to the standard procedure. 78.7 mg (0.500 mmol) of **a23** was used, 3.1 mol% catalyst. Appearance: off-white powder, 61.5 mg (0.482 mmol, 97 % yield). Analytical data: ^{1}H NMR (300 MHz, $CDCl_3$, 25 °C): δ = 7.08 (d, J = 9.0 Hz, 2H), 6.58 (d, J = 9.0 Hz, 2H), 3.63 (br, 2H) ppm; ^{13}C{^{1}H} NMR (75.5 MHz, $CDCl_3$, 25 °C): δ = 145.0, 129.2, 123.2, 116.3 ppm; HR-MS (ESI-Orbitrap) *m/z*: [M-H]$^+$ calcd. for C_6H_7ClN: 128.0262; found: 128.0269.

*3,5-Dichloroaniline (**b24**)*. The title compound was synthesized according to the standard procedure. 95.7 mg (0.498 mmol) of **a24** was used, 3.0 mol% catalyst. Appearance: yellow oil,

71.4 mg (0.440 mmol, 88 % yield). Analytical data: ^1H NMR (300 MHz, $CDCl_3$, 25 °C): δ = 6.75 (s, 1H), 6.55 (s, 2H), 3.82 (br, 2H) ppm; $^{13}C\{^1H\}$ NMR (75.5 MHz, $CDCl_3$, 25 °C): δ = 148.3, 135.4, 118.3, 113.2 ppm; HR-MS (ESI-Orbitrap) *m*/*z*: $[M-H]^+$ calcd. for $C_6H_6Cl_2N$: 161.9872; found: 161.9866.

*2-Bromoanilinium chloride (**c25**)*. The title compound was synthesized according to the standard procedure including the precipitation step. 101.5 mg (0.502 mmol) of **a25** was used, 3.3 mol% catalyst. Appearance: light-brown powder, 47.7 mg (0.229 mmol, 46 % yield). Analytical data: ^1H NMR (300 MHz, D_2O, 25 °C): δ = 7.78 – 7.76 (m, 1H), 7.49 – 7.47 (m, 2H), 7.37 – 7.34 (m, 1H) ppm; $^{13}C\{^1H\}$ NMR (75.5 MHz, D_2O, 25 °C): δ = 133.8, 130.2, 130.0, 129.1, 124.1, 116.2 ppm; HR-MS (ESI-Orbitrap) *m*/*z*:
$[M-Cl]^+$ calcd. for C_6H_7BrN: 171.9756; found: 171.9750.

*3-Bromoanilinium chloride (**c26**)*. The title compound was synthesized according to the standard procedure including the precipitation step. 101.6 mg (0.503 mmol) of **a26** was used, 3.3 mol% catalyst. Appearance: light-brown powder, 73.1 mg (0.350 mmol, 70 % yield). Analytical data: ^1H NMR (300 MHz, D_2O, 25 °C): δ = 7.67 – 7.65 (m, 1H), 7.61 – 7.60 (m, 1H), 7.45 – 7.44 (m, 1H), 7.37 – 7.35 (m, 1H) ppm; $^{13}C\{^1H\}$ NMR (75.5 MHz, D_2O, 25 °C): δ = 132.4, 131.5, 131.5, 125.5, 122.6, 121.4 ppm; HR-MS (ESI-Orbitrap) *m*/*z*: $[M-Cl]^+$ calcd. for C_6H_7BrN: 171.9756; found: 171.9755.

*4-Bromoaniline (**b27**)*. The title compound was synthesized according to the standard procedure. 101.6 mg (0.503 mmol) of **a27** was used, 2.9 mol% catalyst. Appearance: colorless oil, 81.1 mg (0.471 mmol, 94 % yield). Analytical data: ^1H NMR (300 MHz, $CDCl_3$, 25 °C): δ = 7.23 (d, J = 8.8 Hz, 2H), 6.55 (d, J = 8.8 Hz, 2H), 3.66 (br, 2H) ppm; $^{13}C\{^1H\}$ NMR (75.5 MHz, $CDCl_3$,

25 °C): δ = 145.4, 132.0, 116.7, 110.2 ppm; HR-MS (ESI-Orbitrap) *m*/*z*: [M-H]$^+$ calcd. for C_6H_7BrN: 171.9756; found: 171.9758.

*2-Iodoanilinium chloride (**c28**)*. The title compound was synthesized according to the standard procedure including the precipitation step. 124.3 mg (0.499 mmol) of **a28** was used, 3.0 mol% catalyst. Appearance: light-brown powder, 112.6 mg (0.441 mmol, 88 % yield). Analytical data: ^{1}H NMR (300 MHz, D_2O, 25 °C): δ = 8.06 – 8.03 (m, 1H), 7.54 – 7.46 (m, 2H), 7.26 – 7.20 (m, 1H) ppm; ^{13}C{^{1}H} NMR (75.5 MHz, D_2O, 25 °C): δ = 145.1, 129.3, 123.3, 116.4 ppm; HR-MS (ESI-Orbitrap) *m*/*z*: [M-Cl]$^+$ calcd. for C_6H_7IN: 219.9618; found: 219.9616.

*3-Iodoanilinium chloride (**c29**)*. The title compound was synthesized according to the standard procedure including the precipitation step. 124.7 mg (0.501 mmol) of **a29** was used, 2.9 mol% catalyst. Appearance: light-brown powder, 84.4 mg (0.330 mmol, 66 % yield). Analytical data: ^{1}H NMR (300 MHz, D_2O, 25 °C): δ = 7.90 – 7.88 (m, 1H), 7.80 (s, 1H), 7.43 – 7.40 (m, 1H), 7.32 – 7.27 (m, 1H) ppm; ^{13}C{^{1}H} NMR (75.5 MHz, D_2O, 25 °C): δ = 132.4, 131.5, 131.5, 125.5, 122.6, 121.4 ppm; HR-MS (ESI-Orbitrap) *m*/*z*: [M-Cl]$^+$ calcd. for C_6H_7IN: 219.9618; found: 219.9617.

*2,4-Diaminiumdiphenylaminium trichloride (**c30**)*. The title compound was synthesized according to the standard procedure including the precipitation step. 129.9 mg (0.501 mmol) of **a30** was used, 5 mol% catalyst. Appearance: violet powder, 135.5 mg (0.439 mmol, 88 % yield). Analytical data: ^{1}H NMR (300 MHz, D_2O, 25 °C): δ = 7.44 – 7.41 (m, 1H), 7.37 – 7.32 (m, 3H), 7.26 – 7.23 (m, 1H), 7.04 – 6.99 (m, 1H), 6.95 - 6.92 (m, 2H) ppm; ^{13}C{^{1}H} NMR (75.5 MHz, D_2O, 25 °C): δ = 143.9, 135.5, 130.1, 129.7, 126.0, 124.8, 121.2, 121.1, 117.0, 116.4 ppm; HR-MS (ESI-Orbitrap) *m*/*z*: [M-Cl]$^+$ calcd. for $C_{12}H_{14}N_3$: 200.1182; found: 200.1183.

6-Aminoquinoline (***b31***). The title compound was synthesized according to the standard procedure. 87.3 mg (0.501 mmol) of **a31** was used, 3.1 mol% catalyst. Appearance: dark-brown powder, 67.5 mg (0.468 mmol, 93 % yield). Analytical data: ^{1}H NMR (300 MHz, $CDCl_3$, 25 °C): δ = 8.77 (s, 1H), 8.07 – 8.04 (m, 1H), 7.36 – 7.34 (m, 2H), 6.94 – 6.92 (m, 2H), 5.01 (br, 2H) ppm; ^{13}C{^{1}H} NMR (75.5 MHz, $CDCl_3$, 25 °C): δ = 147.5, 144.1, 138.5, 136.1, 128.9, 127.5, 121.4, 116.1, 110.1 ppm; HR-MS (ESI-Orbitrap) *m/z*: $[M\text{-}H]^+$ calcd. for $C_9H_9N_2$: 145.0760; found: 145.0762.

8-Aminoquinoline (***b32***). The title compound was synthesized according to the standard procedure. 87.8 mg (0.504 mmol) of **a32** was used, 3.1 mol% catalyst. Appearance: light-brown powder, 65.8 mg (0.456 mmol, 91 % yield). Analytical data: ^{1}H NMR (300 MHz, $CDCl_3$, 25 °C): δ = 8.77 (s, 1H), 8.07 – 8.04 (m, 1H), 7.36 – 7.34 (m, 2H), 6.94 – 6.92 (m, 2H), 5.01 (br, 2H) ppm; ^{13}C{^{1}H} NMR (75.5 MHz, $CDCl_3$, 25 °C): δ = 147.5, 144.1, 138.5, 136.1, 128.9, 127.5, 121.4, 116.1, 110.1 ppm; HR-MS (ESI-Orbitrap) *m/z*: $[M\text{-}H]^+$ calcd. for $C_9H_9N_2$: 145.0760; found: 145.0761.

4-Aminothiophenol (***b33***). The title compound was synthesized according to the standard procedure. 77.6 mg (0.500 mmol) of **a33** was used, 3.4 mol% catalyst. Appearance: yellow-brownish oil, 56.2 mg (0.478 mmol, 90 % yield). Analytical data: ^{1}H NMR (300 MHz, $CDCl_3$, 25 °C): δ = 7.27 (d, J = 8.9 Hz, 2H), 6.60 (d, J = 8.9 Hz, 2H), 3.78 (s, 2H) ppm; ^{13}C{^{1}H} NMR (75.5 MHz, $CDCl_3$, 25 °C): δ = 147.1, 133.9, 125.8, 115.4 ppm; HR-MS (ESI-Orbitrap) *m/z*: $[M+H]^+$ calcd. for C_6H_8NS: 126.0372; found: 126.0370.

5-Aminothiophene-2-carbonitrile (***b34***). The title compound was synthesized according to the standard procedure. 76.9 mg (0.499 mmol) of **a34** was used, 3.0 mol% catalyst. Appearance: yellow oil, 61.0 mg (0.491 mmol, 98 % yield). Analytical data: ^{1}H NMR (300 MHz, $CDCl_3$, 25 °C): δ = 7.25 (d, J = 4.1 Hz, 1H), 6.08 (d, J = 4.1 Hz, 1H), 4.43 (br, 2H) ppm; ^{13}C{^{1}H} NMR (75.5 MHz,

CDCl$_3$, 25 °C): δ = 158.4, 138.9, 115.8, 107.4, 93.6 ppm; HR-MS (ESI-Orbitrap) *m/z*: [M+H]$^+$ calcd. for $C_5H_5N_2S$: 125.0168; found: 125.0167.

*Methyl 3-phenylpropanoate (**e1**)*. The title compound was synthesized according to the standard procedure. 81.9 mg (0.505 mmol) of **d1** was used, 3.3 mol% catalyst. Appearance: colorless oil, 80.2 mg (0.488 mmol, 98 % yield). Analytical data: ^{1}H NMR (300 MHz, CDCl$_3$, 25 °C): δ = 7.23 – 7.17 (m, 2H), 7.14 – 7.11 (m, 3H), 3.59 (s, 3H), 2.90 – 2.85 (m, 2H), 2.58 – 2.53 (m, 2H) pp m; ^{13}C{^{1}H}NMR (75.5 MHz, CDCl$_3$, 25 °C): δ = 172.3, 140.0, 127.8, 127.6, 125.5, 50.6, 34.9, 30.2, 29.9 ppm; HR-MS (ESI-Orbitrap) *m/z*: [M+H]$^+$ calcd. for $C_{10}H_{15}O_2$: 165.0910; found: 165.0915.

*Ethyl 3-phenylpropanoate (**e2**)*. The title compound was synthesized according to the standard procedure. 89.8 mg (0.510 mmol) of **d2** was used, 2.8 mol% catalyst. Appearance: colorless oil, 88.1 mg (0.494 mmol, 96 % yield). Analytical data: ^{1}H NMR (300 MHz, CDCl$_3$, 25 °C): δ = 7.35 – 7.21 (m, 5H), 4.19 – 4.12 (m, 2H), 3.01 – 2.96 (m, 2H), 2.68 – 2.63 (m, 2H), 1.29 – 1.24 (m, 3H) ppm; ^{13}C{^{1}H}NMR (75.5 MHz, CDCl$_3$, 25 °C): δ = 172.9, 140.6, 128.5, 128.3, 126.3, 60.4, 36.0, 31.0, 14.2 ppm; HR-MS (ESI-Orbitrap) *m/z*: [M+H]$^+$ calcd. for $C_{11}H_{15}O_2$: 179.1067; found: 179.1068.

*Isobutyl 3-phenylpropanoate (**e3**)*. The title compound was synthesized according to the standard procedure. 122.5 (0.600 mmol) of **d3** was used, 2.8 mol% catalyst. Appearance: colorless oil, 118.9 mg (0.576 mmol, 96 % yield). Analytical data: ^{1}H NMR (300 MHz, CDCl$_3$, 25 °C): δ = 7.23 – 7.18 (m, 2H), 7.14 – 7.11 (m, 3H), 3.77 (d, J = 7.2 Hz, 2H), 2.91 – 2.86 (m, 2H), 2.59 – 2.54 (m, 2H), 1.86 – 1.78 (m, 1H), 0.82 (d, J = 6.9 Hz, 6H) ppm; ^{13}C{^{1}H}NMR (75.5 MHz, CDCl$_3$, 25 °C): δ = 173.1, 140.7, 128.6, 128.4, 126.4, 70.7, 36.0, 31.2, 27.8, 19.2 ppm; HR-MS (ESI-Orbitrap) *m/z*: [M+H]$^+$ calcd. for $C_{13}H_{19}O_2$: 207.1380; found: 207.1379.

*Methyl 3-(4-chlorophenyl)propanoate (**e4**)*. The title compound was synthesized according to the standard procedure. 98.4 mg (0.500 mmol) of **d4** was used, 3.2 mol% catalyst. Appearance: colorless oil, 96.6 mg (0.486 mmol, 97 % yield). Analytical data: ^{1}H NMR (300 MHz, $CDCl_3$, 25 °C): δ = 7.30 – 7.27 (m, 2H), 7.18 – 7.15 (m, 2H), 3.70 (s, 3H), 2.98 – 2.93 (m, 2H), 2.67 – 2.62 (m, 2H) ppm; ^{13}C{^{1}H}NMR (75.5 MHz, $CDCl_3$, 25 °C): δ = 173.1, 138.9, 132.1, 126.7, 128.6, 51.7, 35.7, 35.5, 30.3 ppm.

*Methyl 3-(p-tolyl)propanoate (**e5**)*. The title compound was synthesized according to the standard procedure. 88.0 mg (0.499 mmol) of **d5** was used, 3.0 mol% catalyst. Appearance: colorless oil, 83.0 mg (0.466 mmol, 93 % yield). Analytical data: ^{1}H NMR (300 MHz, $CDCl_3$, 25 °C): δ = 7.12 (s, 4H), 3.69 (s, 3H), 2.96 – 2.91 (m, 2H), 2.66 – 2.61 (m, 2H), 2.34 (s, 3H) ppm; ^{13}C{^{1}H}NMR (75.5 MHz, $CDCl_3$, 25 °C): δ = 173.4, 137.4, 135.8, 129.2, 128.1, 51.6, 35.9, 30.5, 21.0 ppm; HR-MS (ESI-Orbitrap) *m*/*z*: $[M+H]^+$ calcd. for $C_{11}H_{15}O_2$: 179.1067; found: 179.1070.

*Benzyl 3-phenylpropanoate (**e6**)*. The title compound was synthesized according to the standard procedure. 118.9 mg (0.499 mmol) of **d6** was used, 3.3 mol% catalyst. Appearance: colorless oil, 110.9 mg (0.462 mmol, 92 % yield). Analytical data: ^{1}H NMR (300 MHz, $CDCl_3$, 25 °C): δ = 7.38 – 7.34 (m, 7H), 7.32 – 7.21 (m, 3H), 5.15 (s, 2H), 3.02 – 2.99 (m, 2H), 2.75 – 2.70 (m, 2H) ppm; ^{13}C{^{1}H}NMR (75.5 MHz, $CDCl_3$, 25 °C): δ = 172.4, 140.1, 135.6, 128.2, 128.2, 128.0, 127.9, 126.0, 66.0, 35.6, 30.6 ppm; HR-MS (ESI-Orbitrap) *m*/*z*: $[M+H]^+$ calcd. for $C_{16}H_{17}O_2$: 241.1223; found: 241.1220.

*3,3,5-Trimethylcyclohexan-1-one (**e7**)*. The title compound was synthesized according to the standard procedure. 70.3 mg (0.509 mmol) of **d7** was used, 2.8 mol% catalyst. Appearance:

colorless oil, 58.4 mg (0.417 mmol, 82 % yield). Analytical data: ^{1}H NMR (300 MHz, $CDCl_3$, 25 °C): δ = 2.27 – 2.21 (m, 1H), 2.11 – 2.07 (m, 1H), 1.99 – 1.89 (m, 1H), 1.86 – 1.77 (m, 1H), 1.53 – 1.49 (m, 1H), 1.27 – 1.18 (m, 1H), 0.98 – 0.93 (m, 6H), 0.81 (s, 3H) ppm; ^{13}C{^{1}H}NMR (75.5 MHz, $CDCl_3$, 25 °C): δ = 212.1, 54.2, 49.3, 47.3, 35.4, 32.1, 29.7, 25.8, 22.5 ppm.

7. ^{1}H NMR, ^{13}C{^{1}H} NMR, and HR-MS Spectra of the Isolated Products

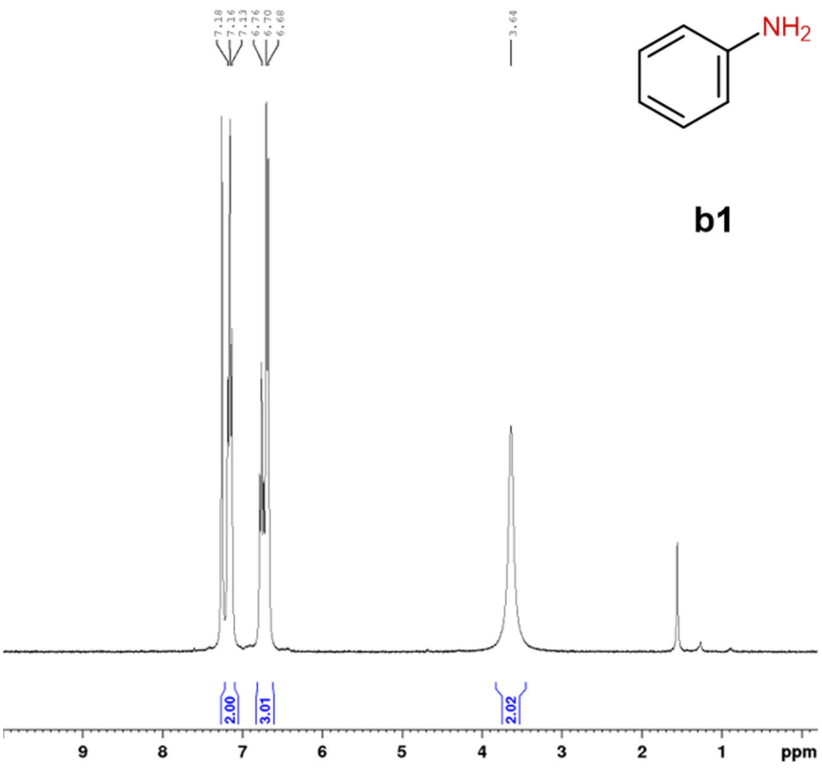

Figure S9. ^{1}H NMR of aniline (**b1**) in $CDCl_3$.

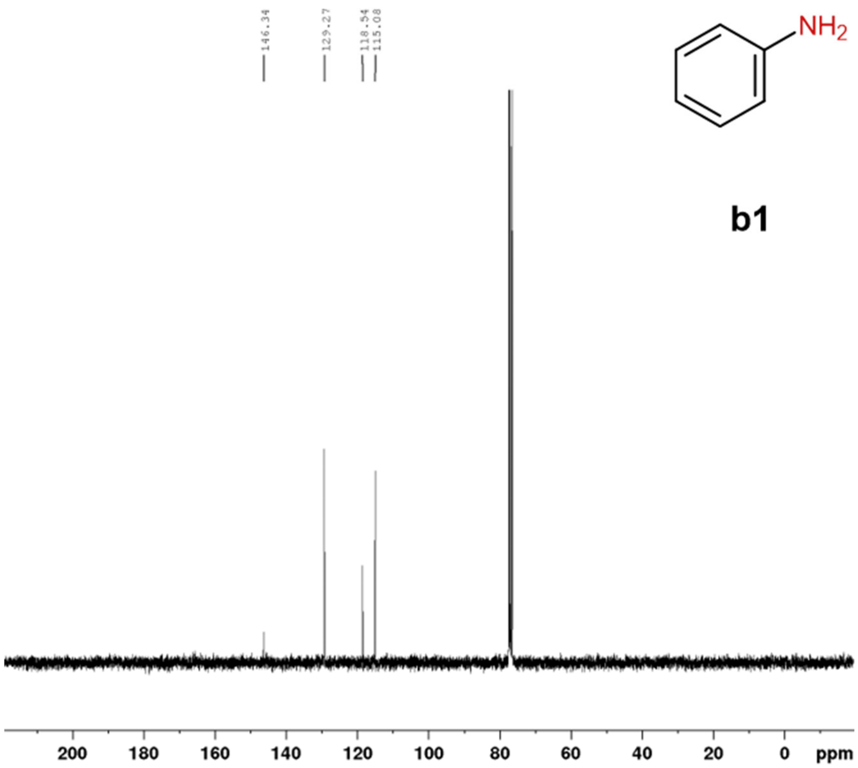

Figure S10. $^{13}C\{^{1}H\}$ NMR of aniline (**b1**) in $CDCl_3$.

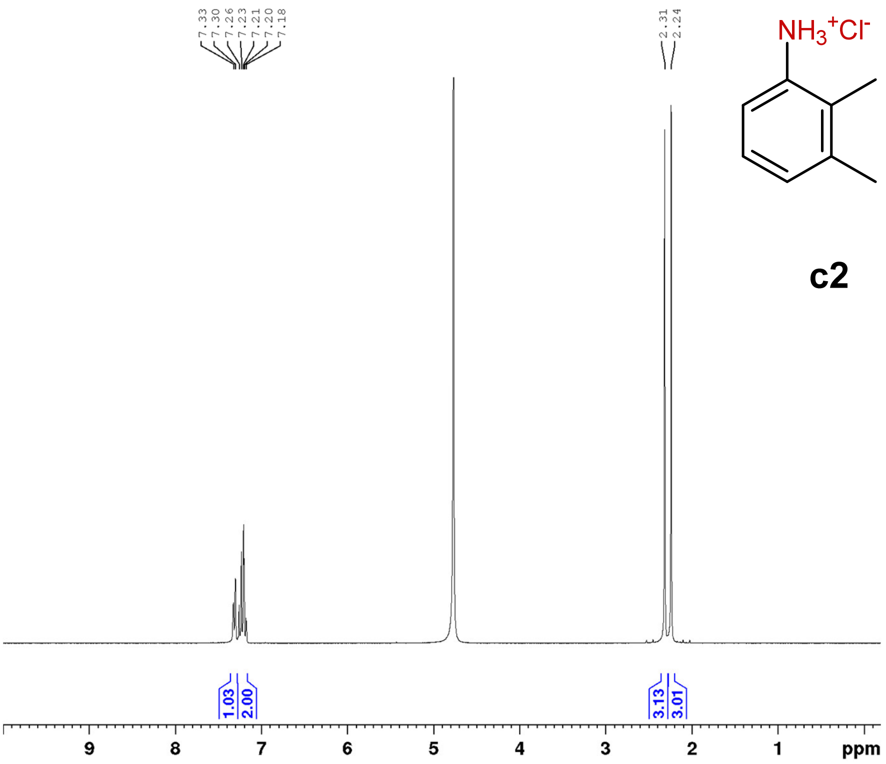

Figure S11. ^{1}H NMR of 2,3-dimethylbenzenaminium chloride (**c2**) in D_2O.

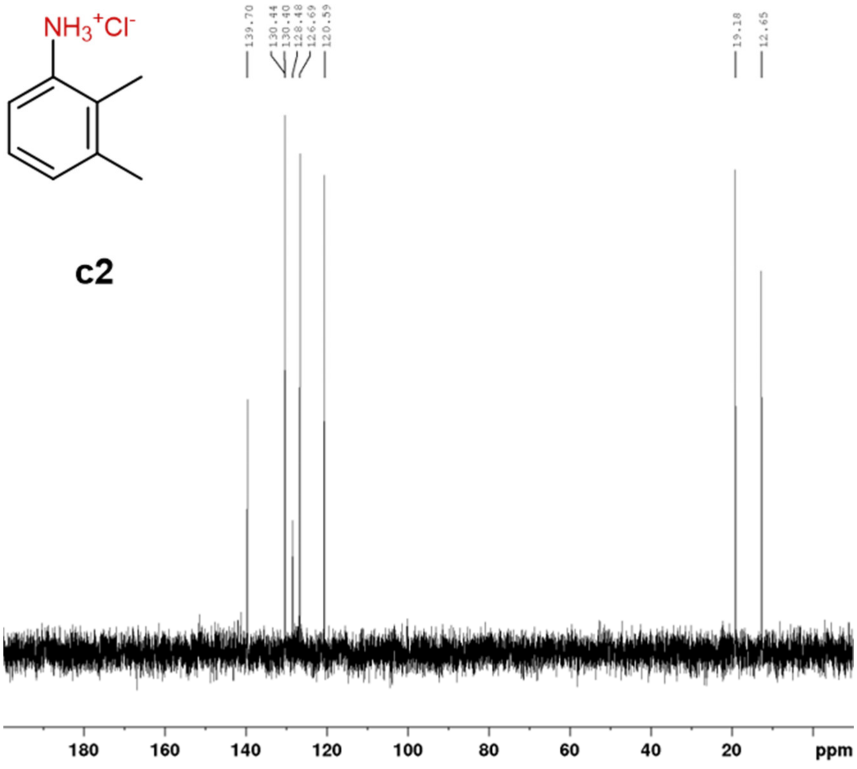

Figure S12. $^{13}C\{^{1}H\}$ NMR of 2,3-dimethylbenzenaminium chloride (**c2**) in D_2O.

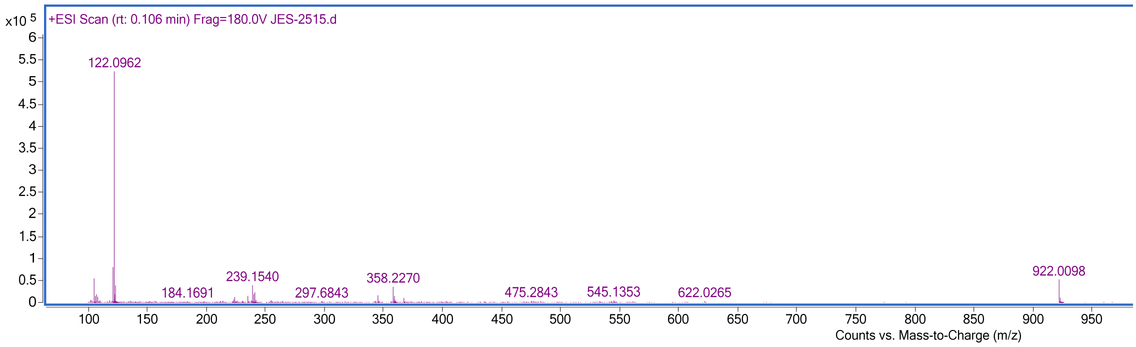

Figure S13. HR-MS of 2,3-dimethylbenzenaminium chloride (**c2**).

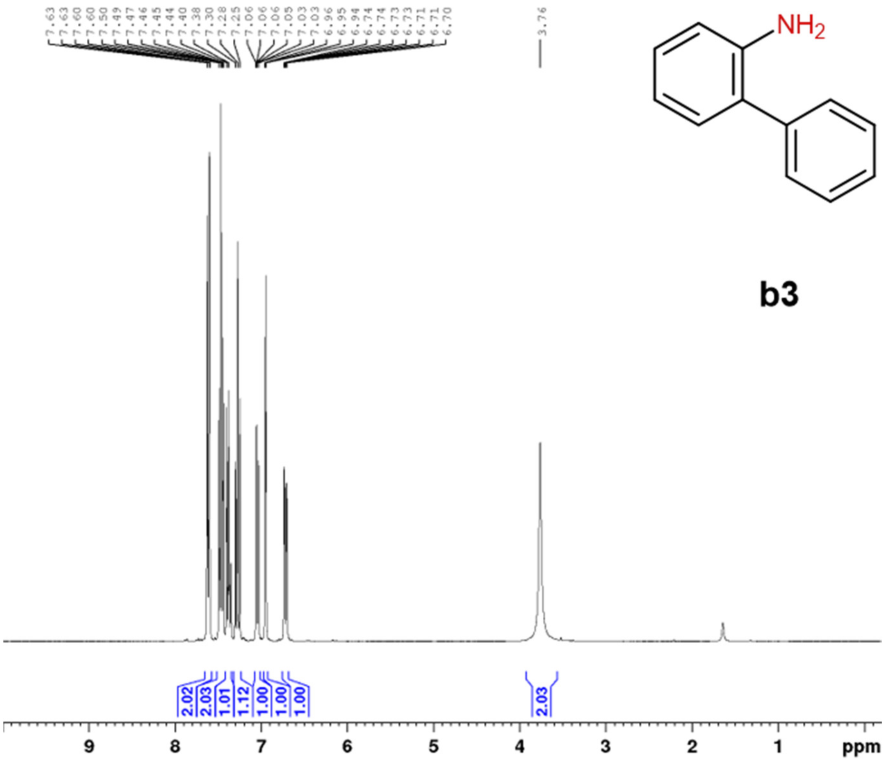

Figure S14. ^{1}H NMR of 2-aminobiphenyl (**b3**) in $CDCl_3$.

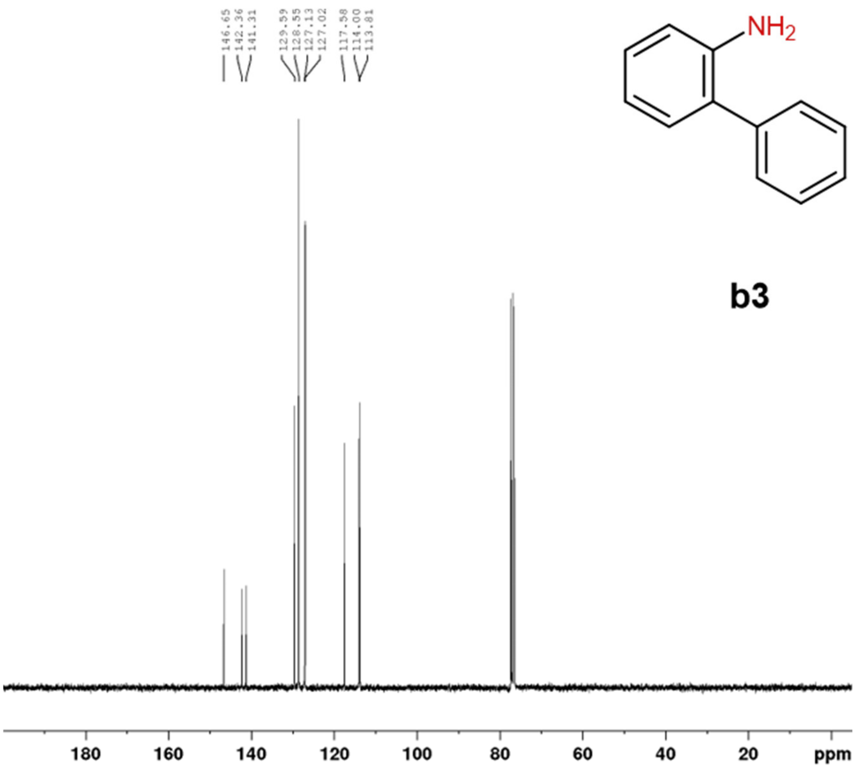

Figure S15. $^{13}C\{^{1}H\}$ NMR of 2-aminobiphenyl (**b3**) in $CDCl_3$.

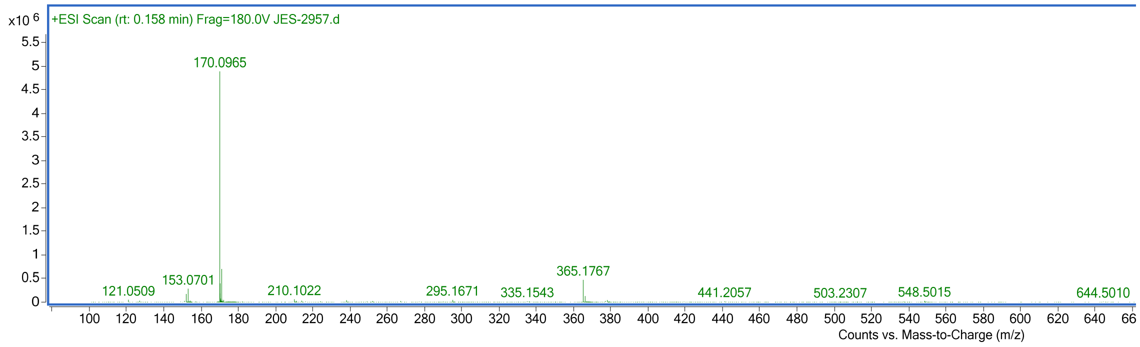

Figure S16. HR-MS of 2-aminobiphenyl (**b3**).

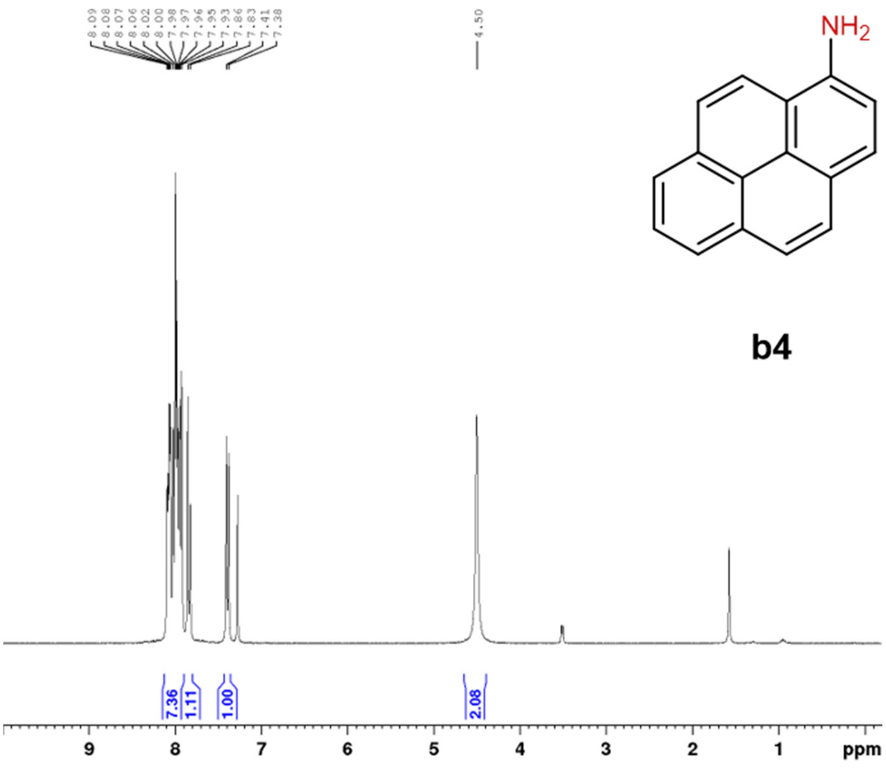

Figure S17. ^{1}H NMR of 1-aminopyrene (**b4**) in $CDCl_3$.

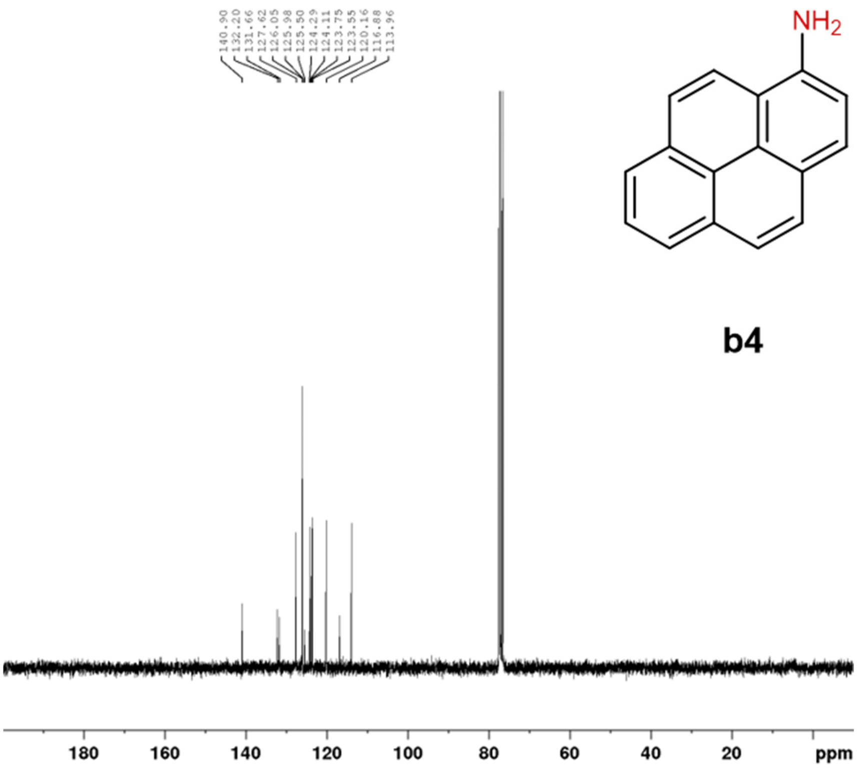

Figure S18. $^{13}C\{^{1}H\}$ NMR of 1-aminopyrene (**b4**) in $CDCl_3$.

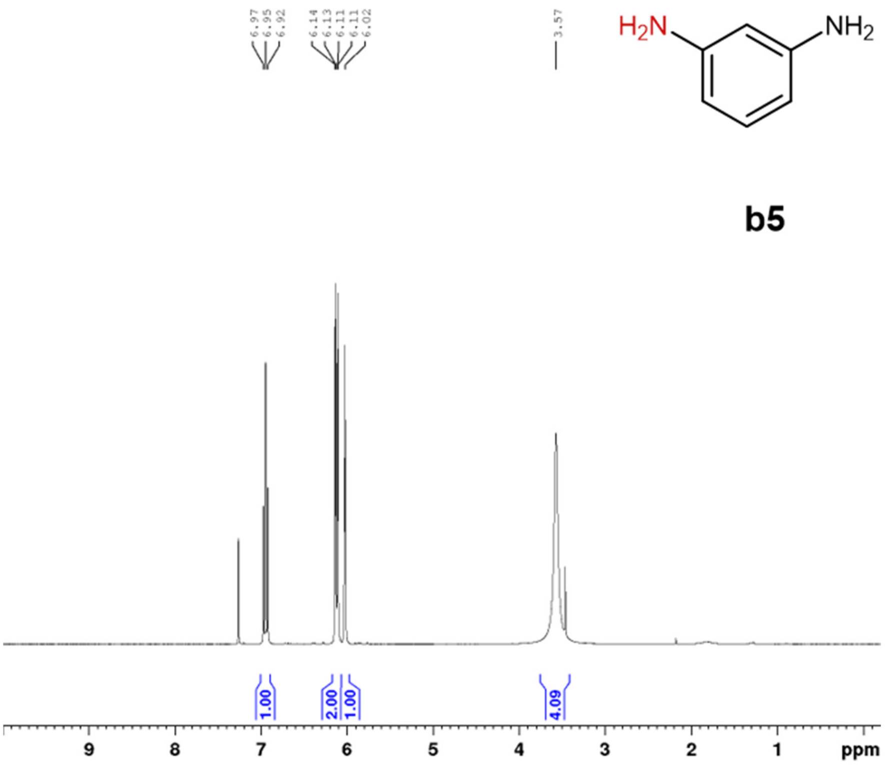

Figure S19. ^{1}H NMR of 1,3-diaminobenzene (**b5**) in $CDCl_3$.

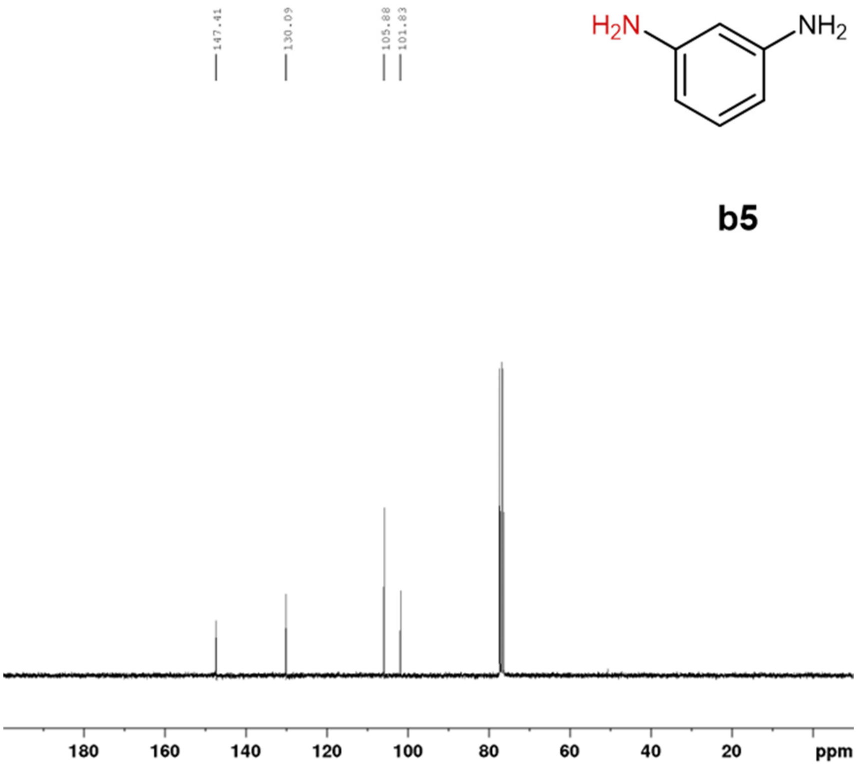

Figure S20. $^{13}C\{^{1}H\}$ NMR of 1,3-diaminobenzene (**b5**) in $CDCl_3$.

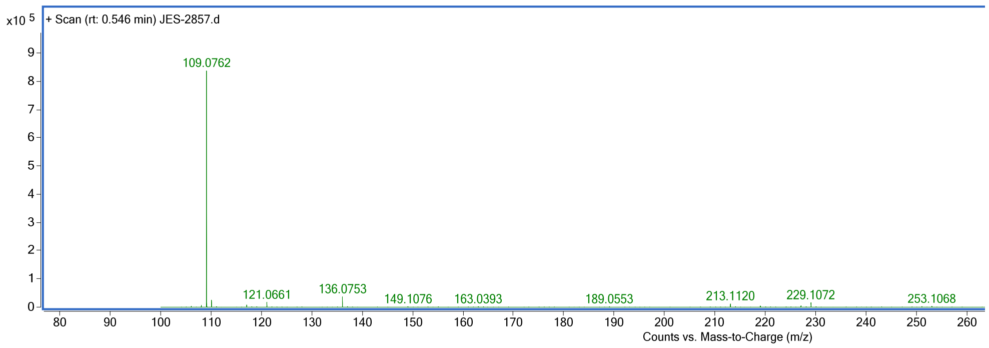

Figure S21. HR-MS of 1,3-diaminobenzene (**b5**).

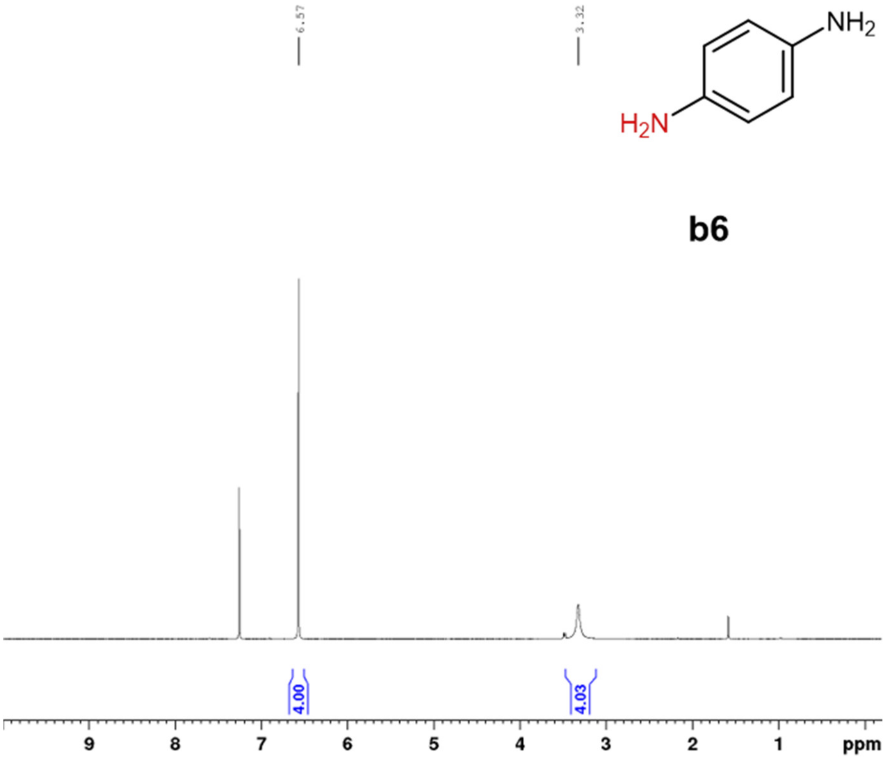

Figure S22. ^{1}H NMR of 1,4-diaminobenzene (**b6**) in $CDCl_3$.

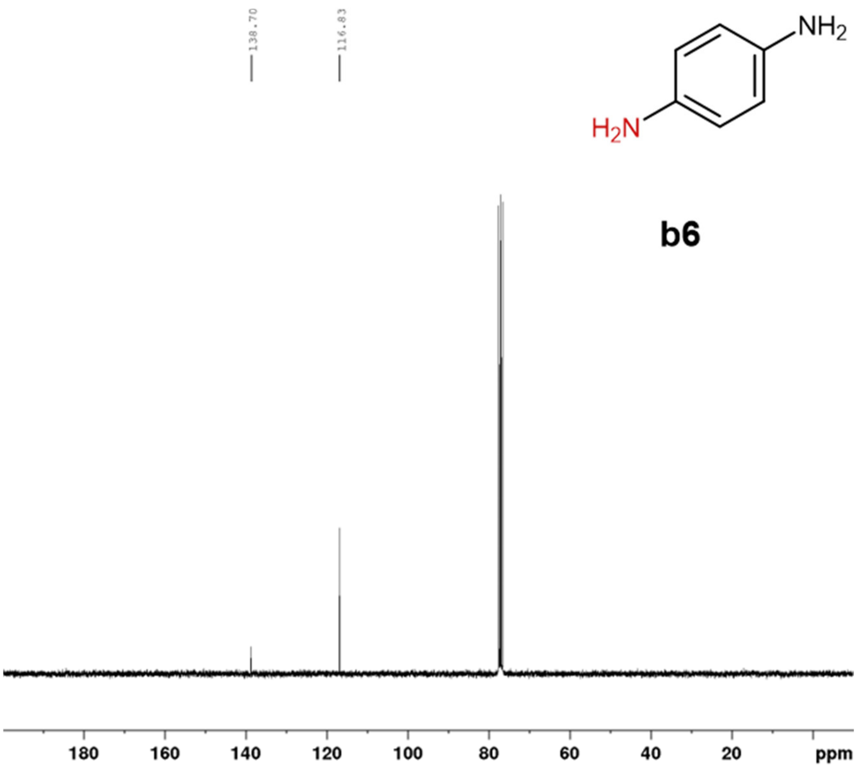

Figure S23. $^{13}C\{^{1}H\}$ NMR of 1,4-diaminobenzene (**b6**) in $CDCl_3$.

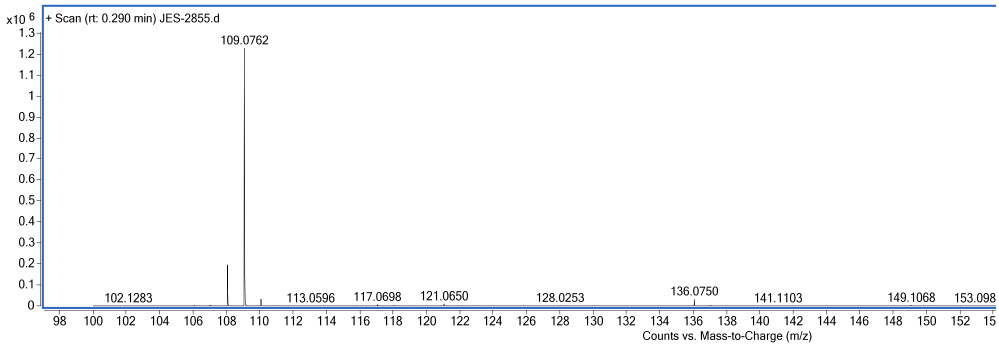

Figure S24. HR-MS of 1,4-diaminobenzene (**b6**).

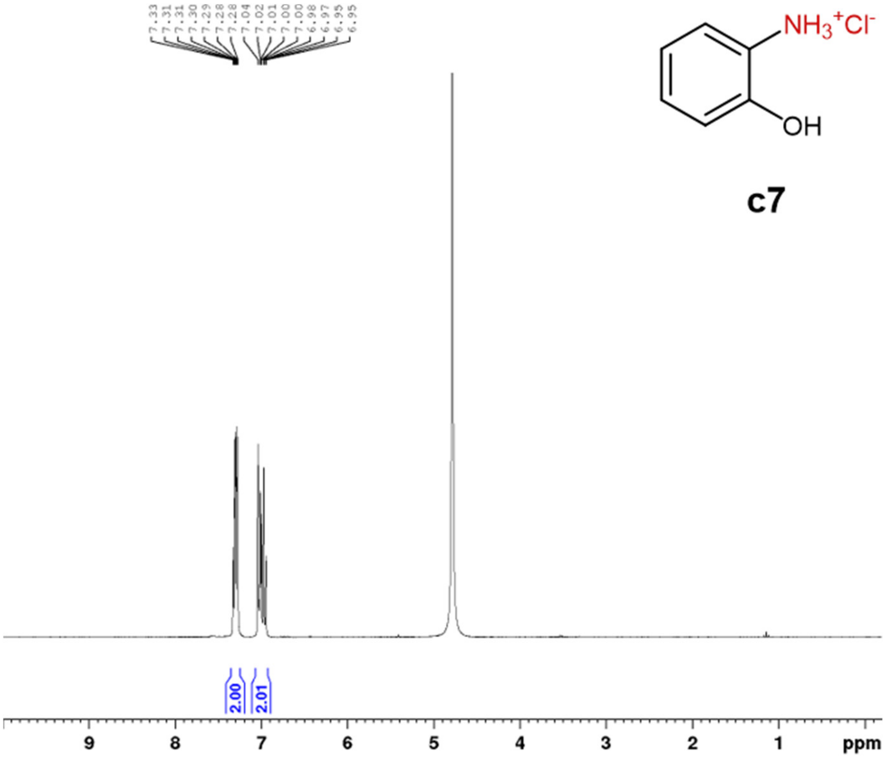

Figure S25. ^{1}H NMR of 2-hydroxybenzenaminium chloride (**c7**) in D_2O.

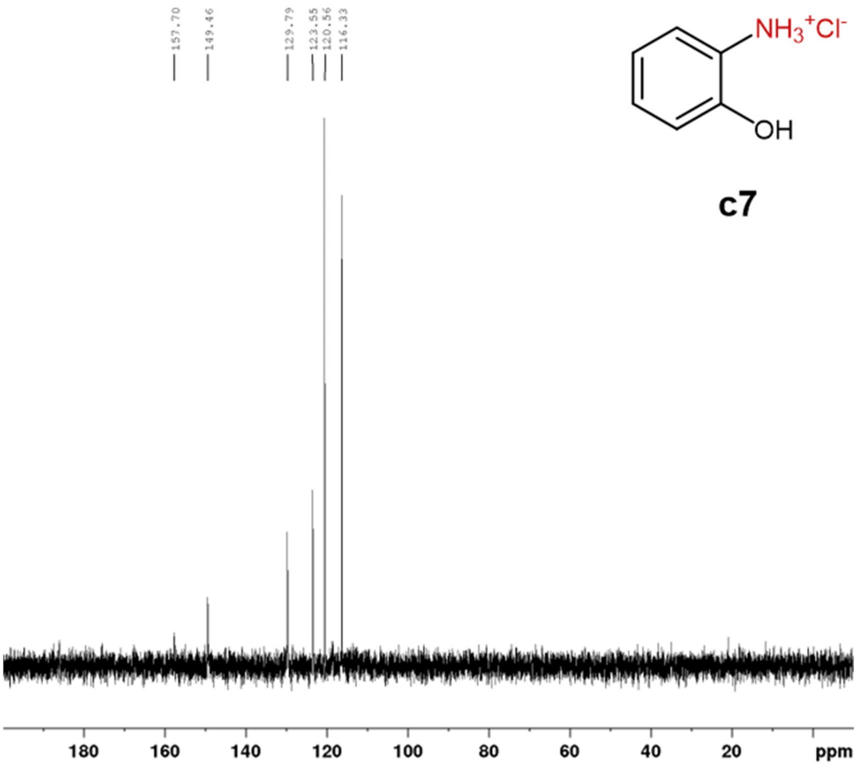

Figure S26. $^{13}C\{^{1}H\}$ NMR of 2-hydroxybenzenaminium chloride (**c7**) in D_2O.

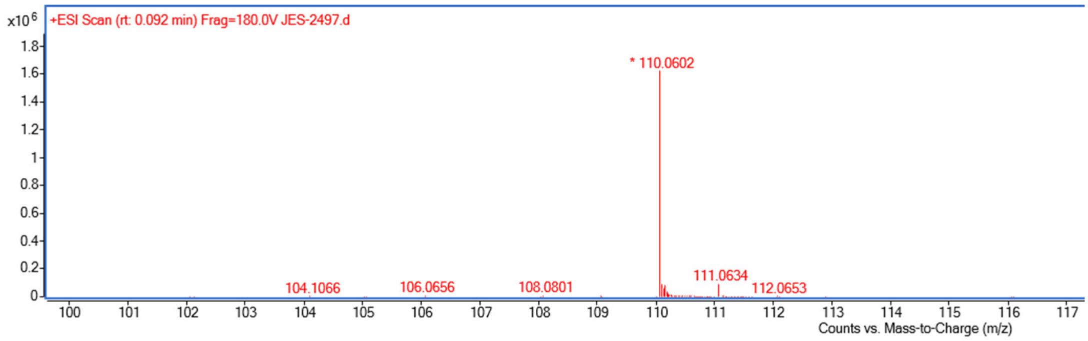

Figure S27. HR-MS of 2-hydroxybenzenaminium chloride (**c7**).

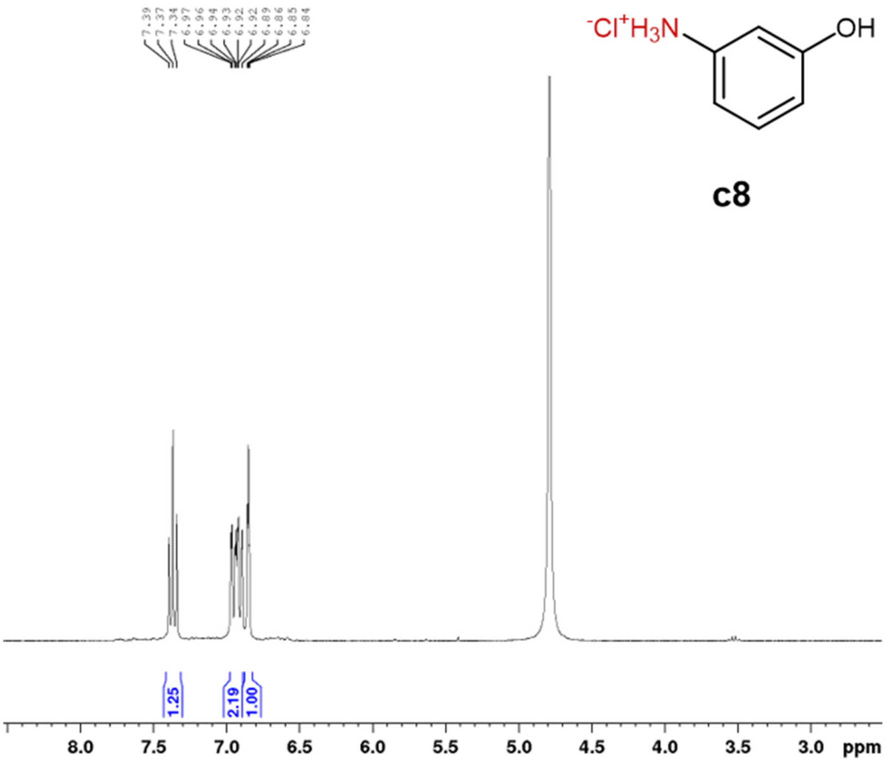

Figure S28. ^{1}H NMR of 3-hydroxybenzenaminium chloride (**c8**) in D_2O.

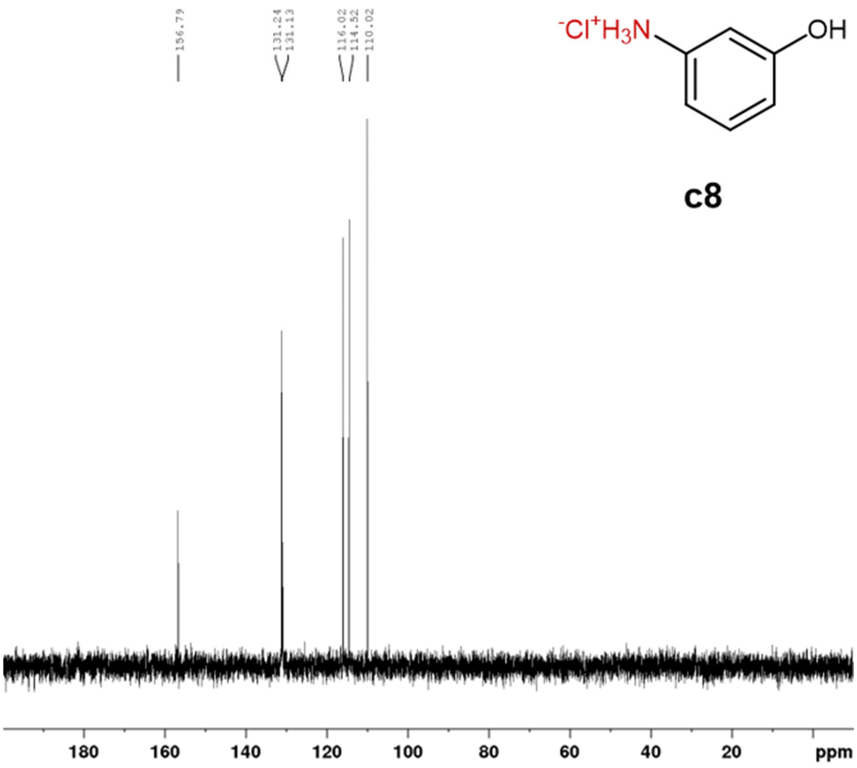

Figure S29. $^{13}C\{^{1}H\}$ NMR of 3-hydroxybenzenaminium chloride (**c8**) in D_2O.

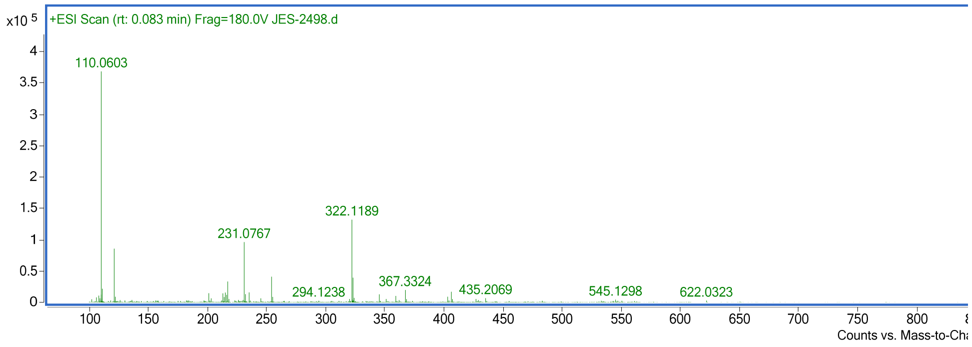

Figure S30. HR-MS of 3-hydroxybenzenaminium chloride (**c8**).

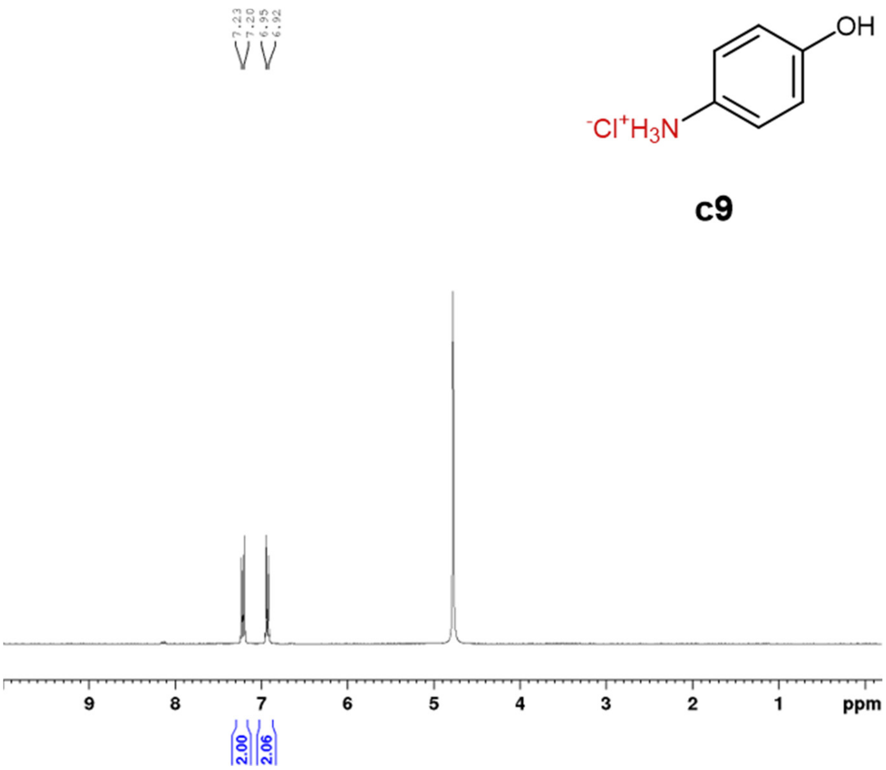

Figure S31. ^{1}H NMR of 4-hydroxybenzenaminium chloride (**c9**) in D_2O.

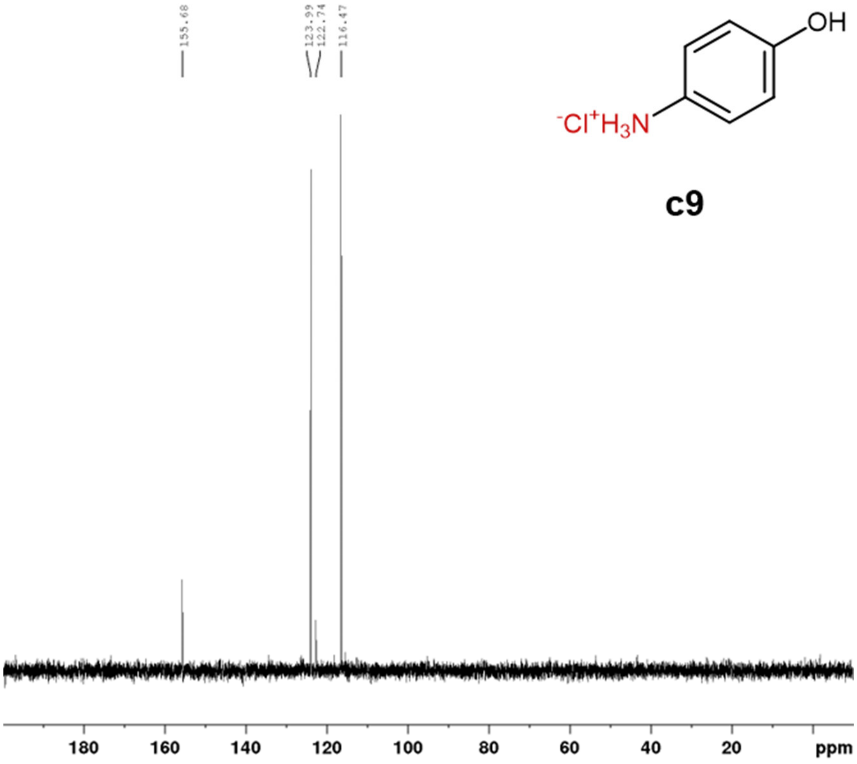

Figure S32. $^{13}C\{^{1}H\}$ NMR of 4-hydroxybenzenaminium chloride (**c9**) in D_2O.

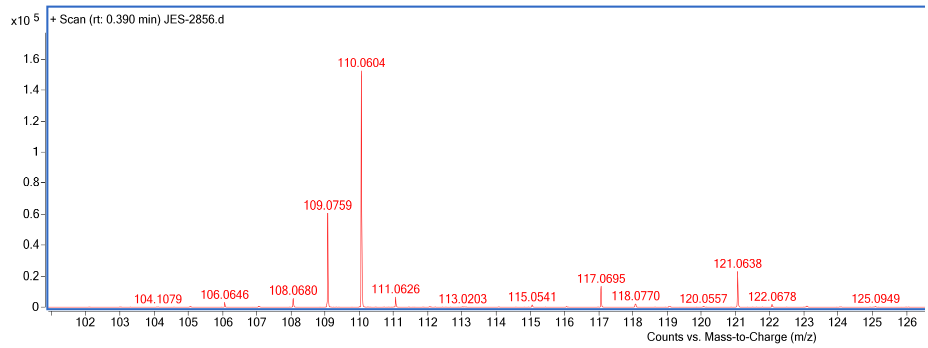

Figure S33. HR-MS of 4-hydroxybenzenaminium chloride (**c9**).

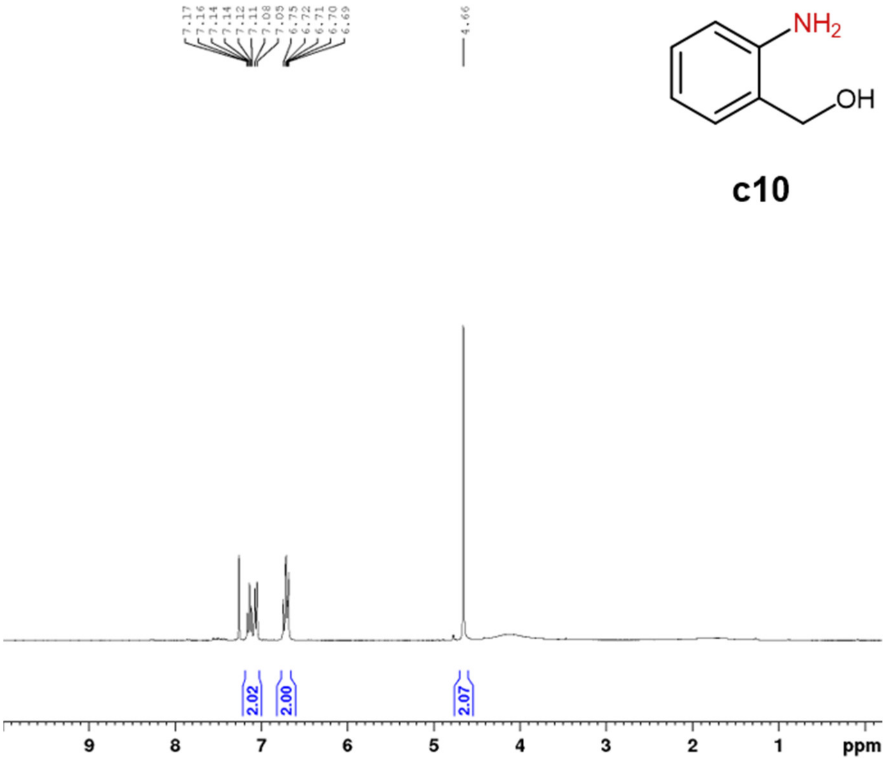

Figure S34. ^{1}H NMR of 2-aminobenzyl alcohol (**b10**) in $CDCl_3$.

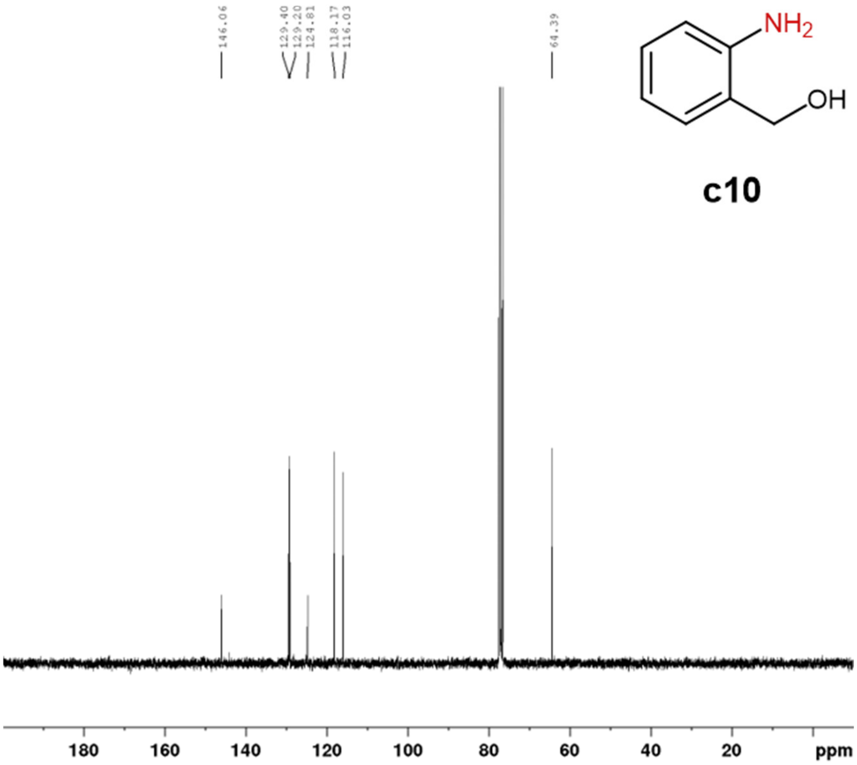

Figure S35. $^{13}C\{^{1}H\}$ NMR of 2-aminobenzyl alcohol (**b10**) in $CDCl_3$.

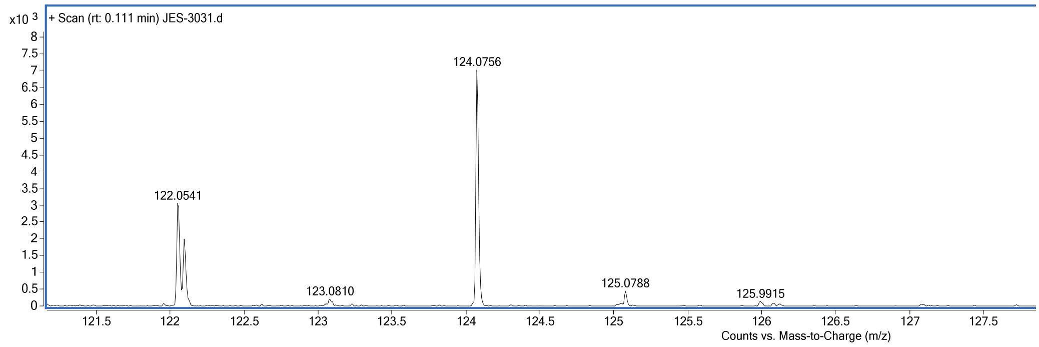

Figure S36. HR-MS of 2-aminobenzyl alcohol (**b10**).

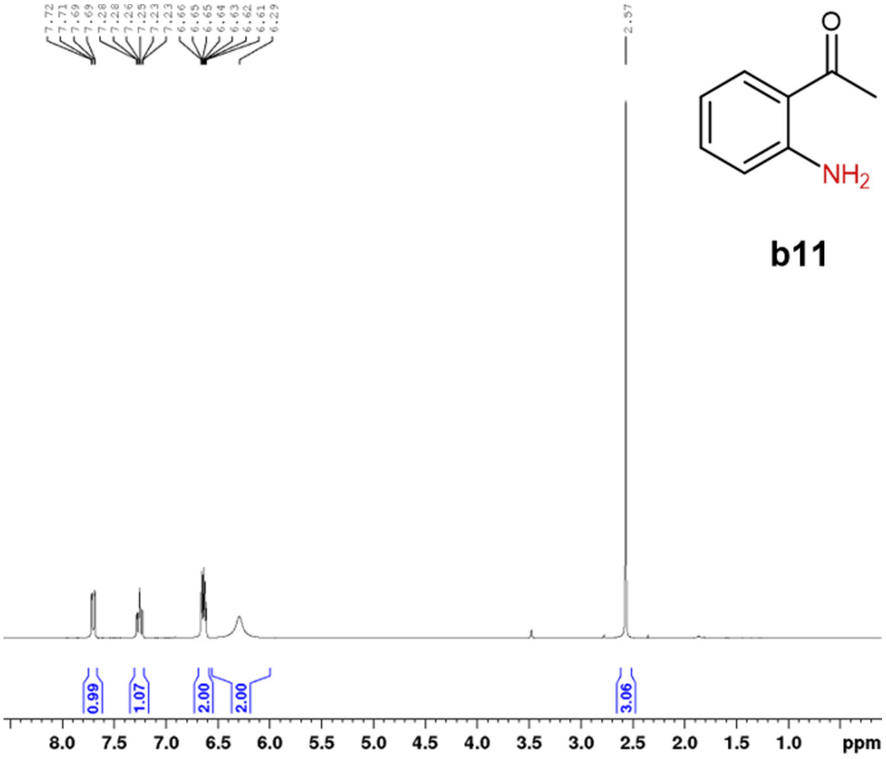

Figure S37. ^{1}H NMR of 2-aminoacetophenone (**b11**) in $CDCl_3$.

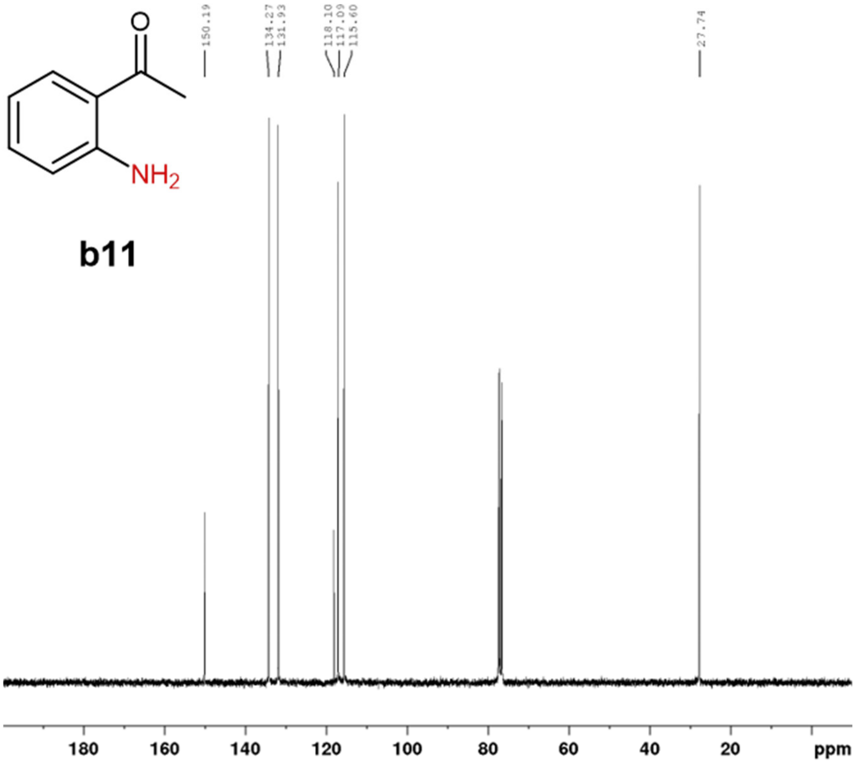

Figure S38. $^{13}C\{^{1}H\}$ NMR of 2-aminoacetophenone (**b11**) in $CDCl_3$.

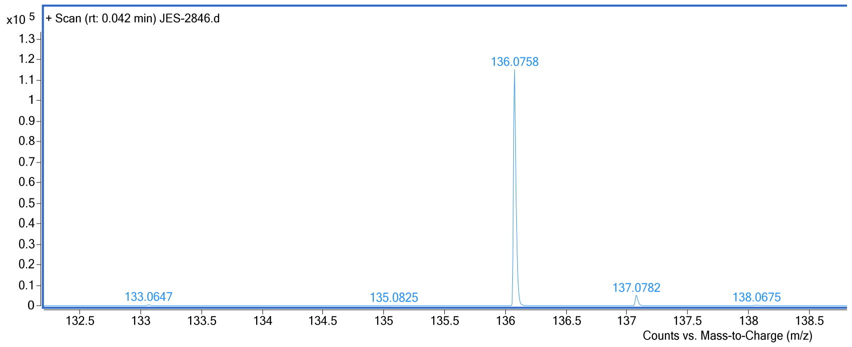

Figure S39. HR-MS of 2-aminoacetophenone (**b11**).

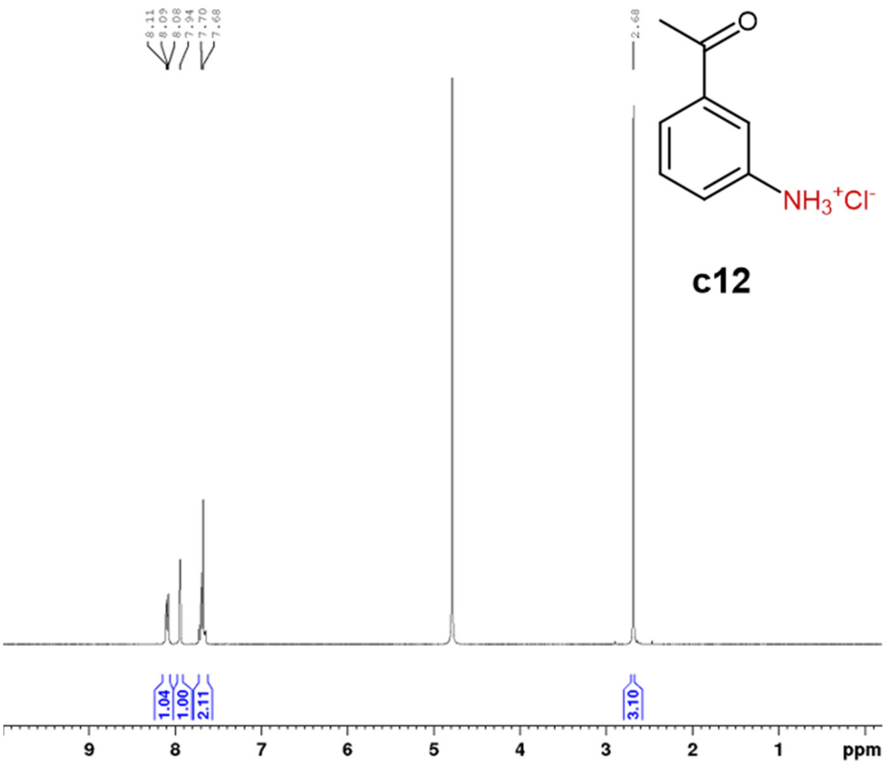

Figure S40. ^{1}H NMR of 3-acetylbenzenaminium chloride (**c12**) in D_2O.

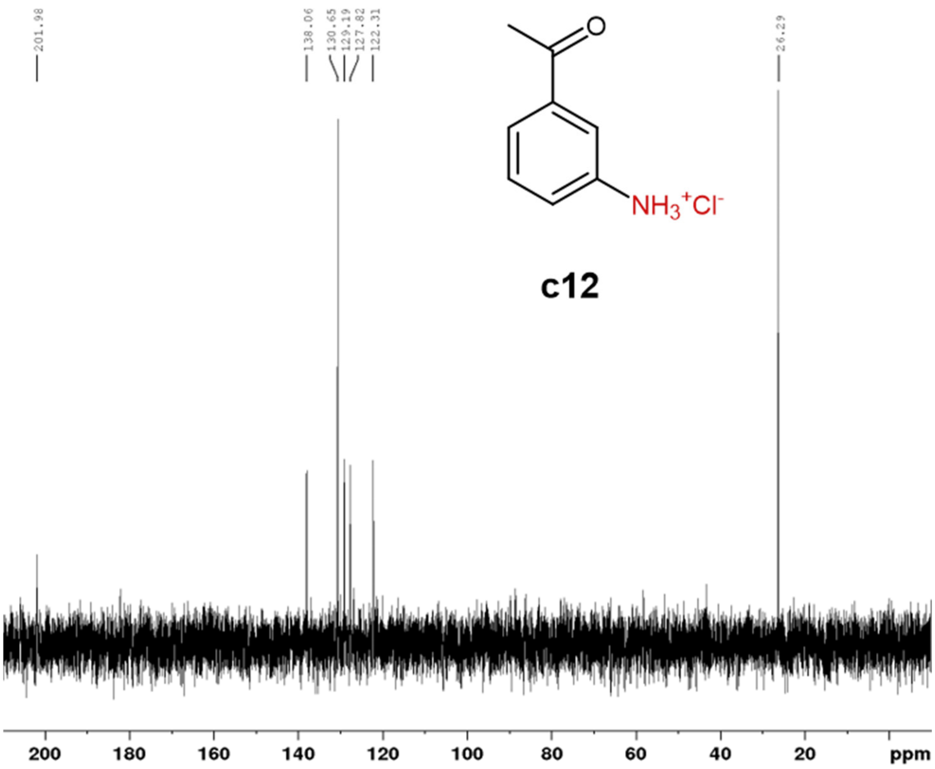

Figure S41. $^{13}C\{^{1}H\}$ NMR of 3-acetylbenzenaminium chloride (**c12**) in D_2O.

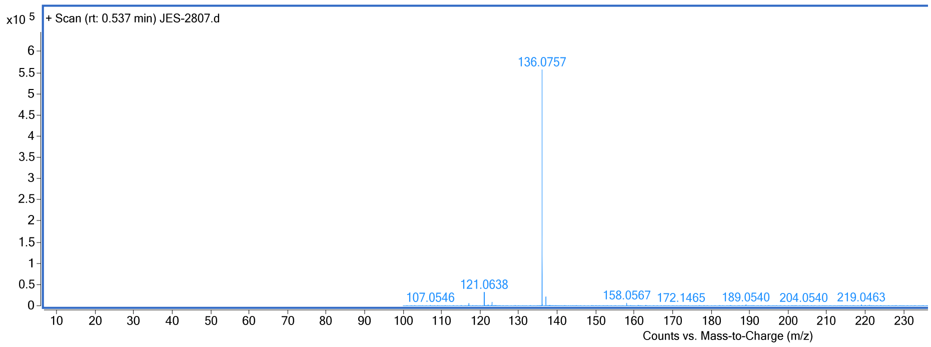

Figure S42. HR-MS of 3-acetylbenzenaminium chloride (**c12**).

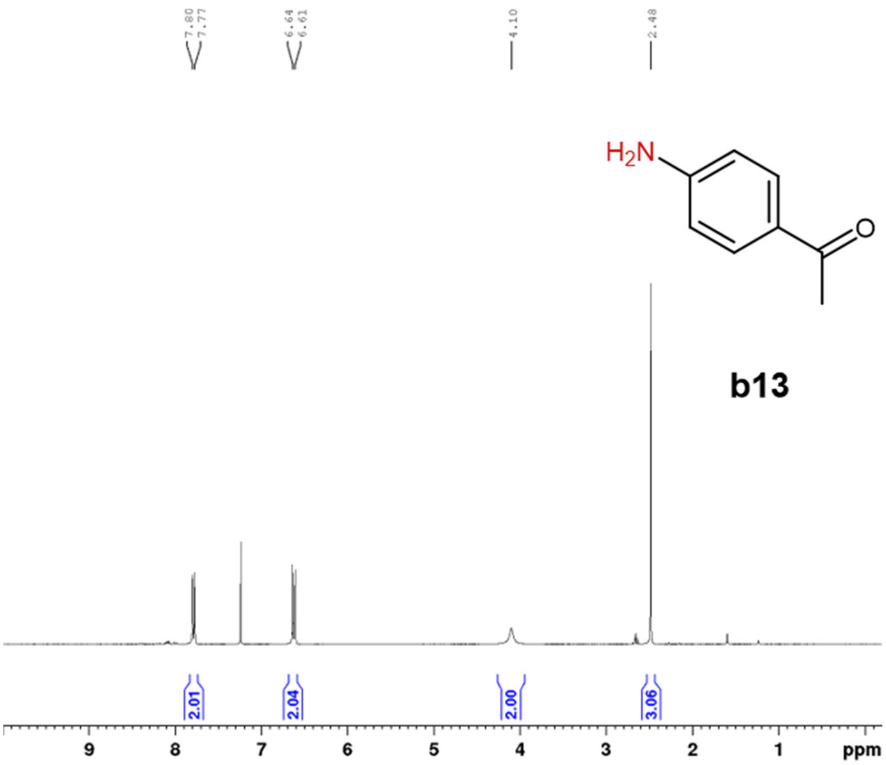

Figure S43. ^{1}H NMR of 4-aminoacetophenone (**b13**) in $CDCl_3$.

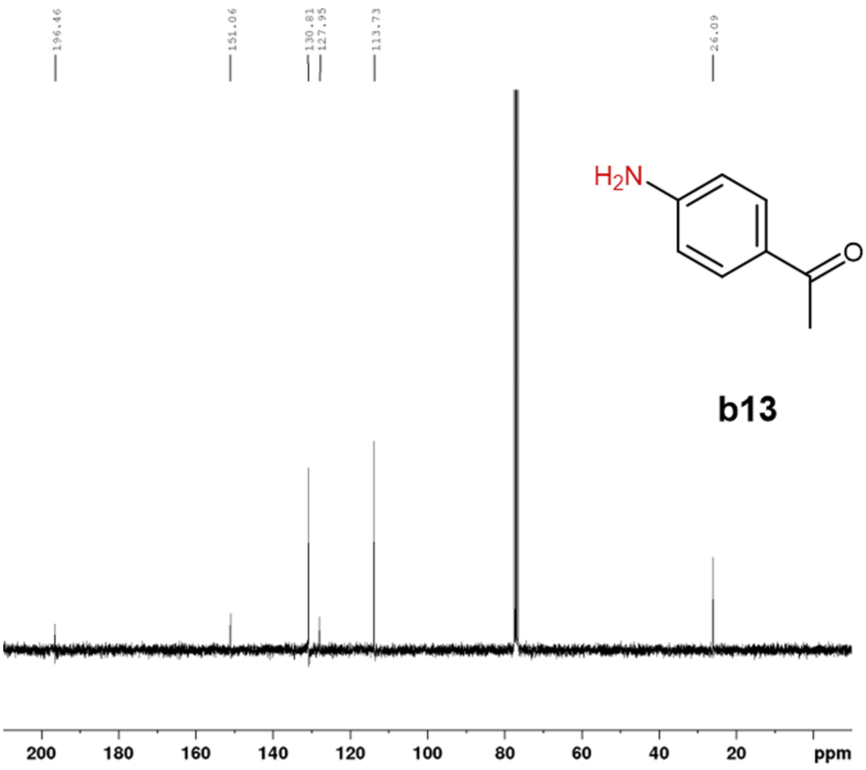

Figure S44. $^{13}C\{^{1}H\}$ NMR of 4-aminoacetophenone (**b13**) in $CDCl_3$.

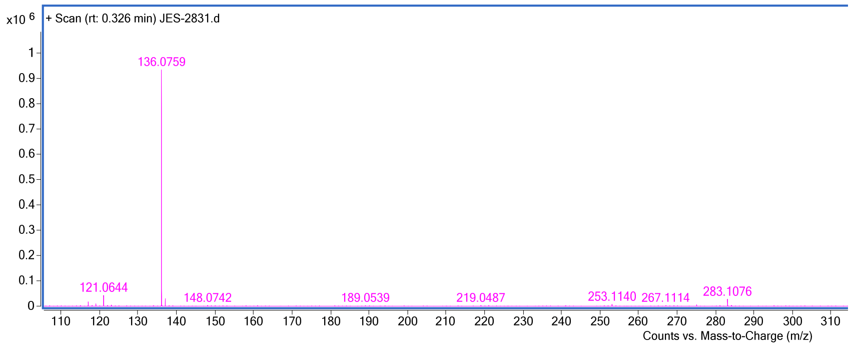

Figure S45. HR-MS of 4-aminoacetophenone (**b13).**

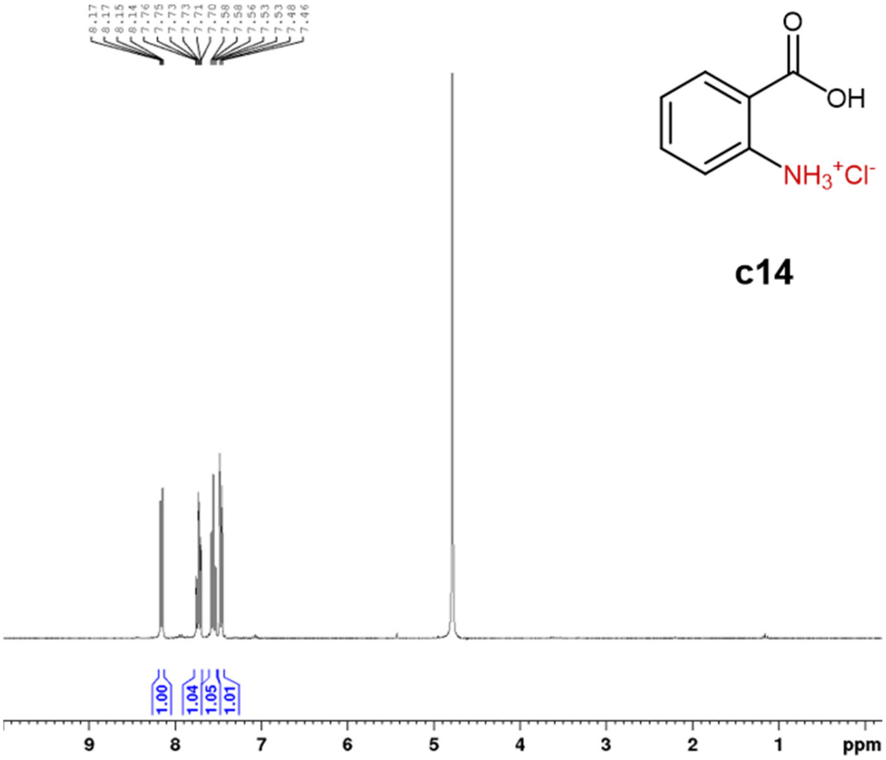

Figure S46. ^{1}H NMR of 2-carboxybenzenaminium chloride (c14) in D_2O.

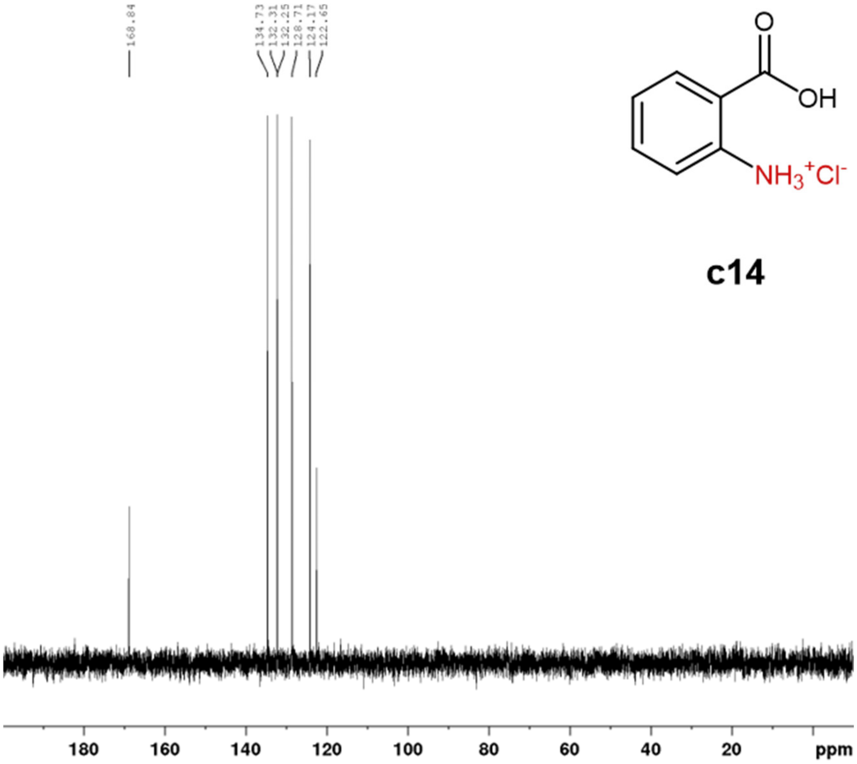

Figure S47. $^{13}C\{^1H\}$ NMR of 2-carboxybenzenaminium chloride (**c14**) in D_2O.

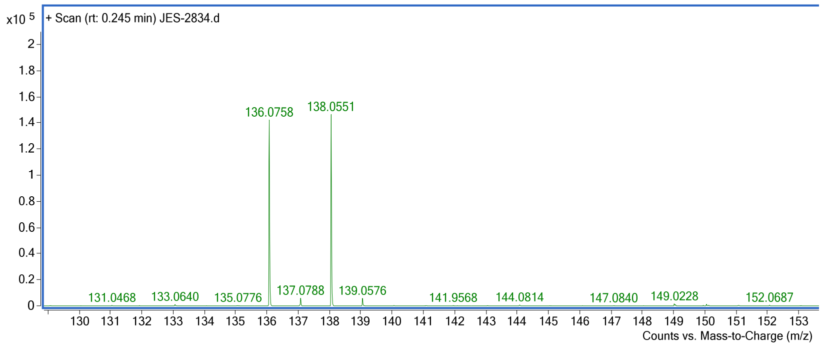

Figure S48. HR-MS of 2-carboxybenzenaminium chloride (**c14**).

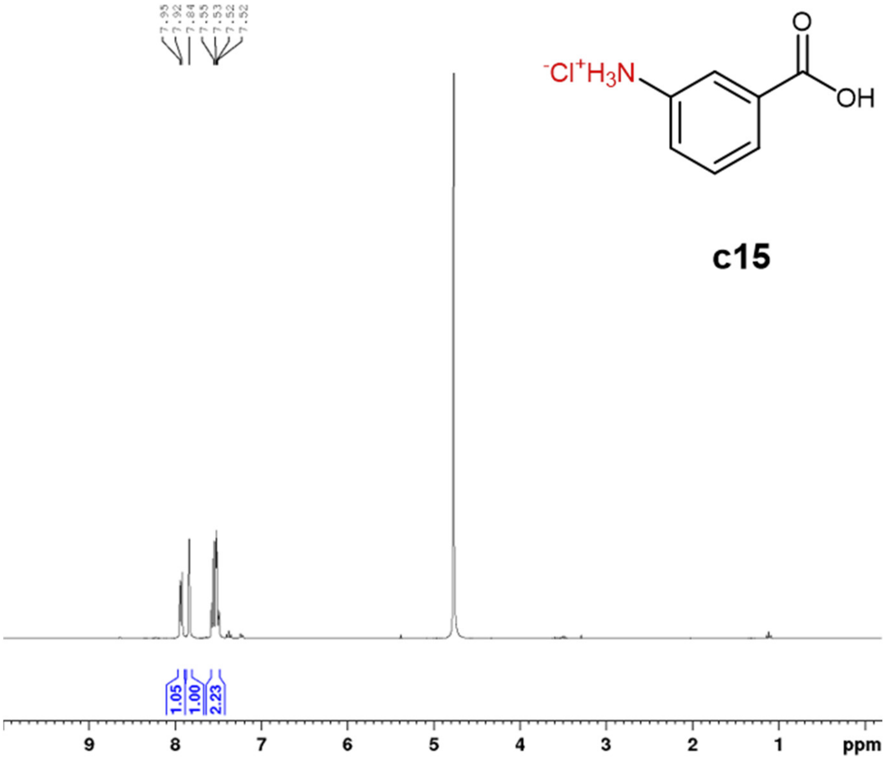

Figure S49. ^{1}H NMR of 3-carboxybenzenaminium chloride (**c15**) in D_2O.

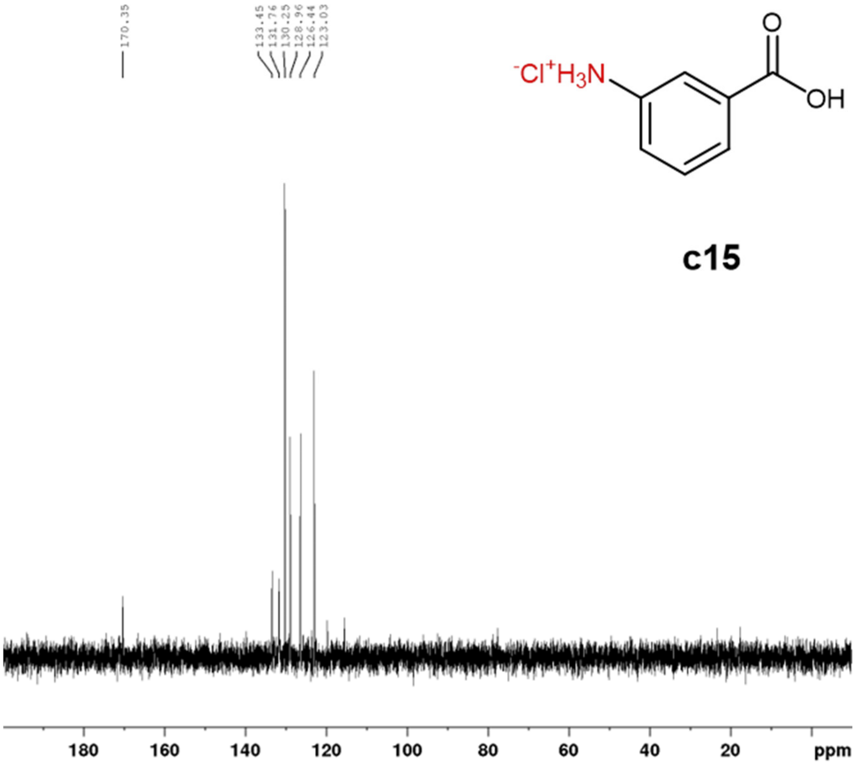

Figure S50. $^{13}C\{^{1}H\}$ NMR of 3-carboxybenzenaminium chloride (**c15**) in D_2O.

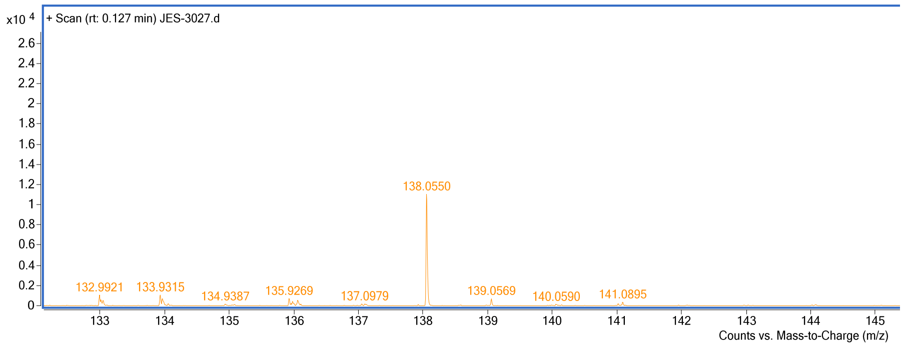

Figure S51. HR-MS of 3-carboxybenzenaminium chloride (**c15**).

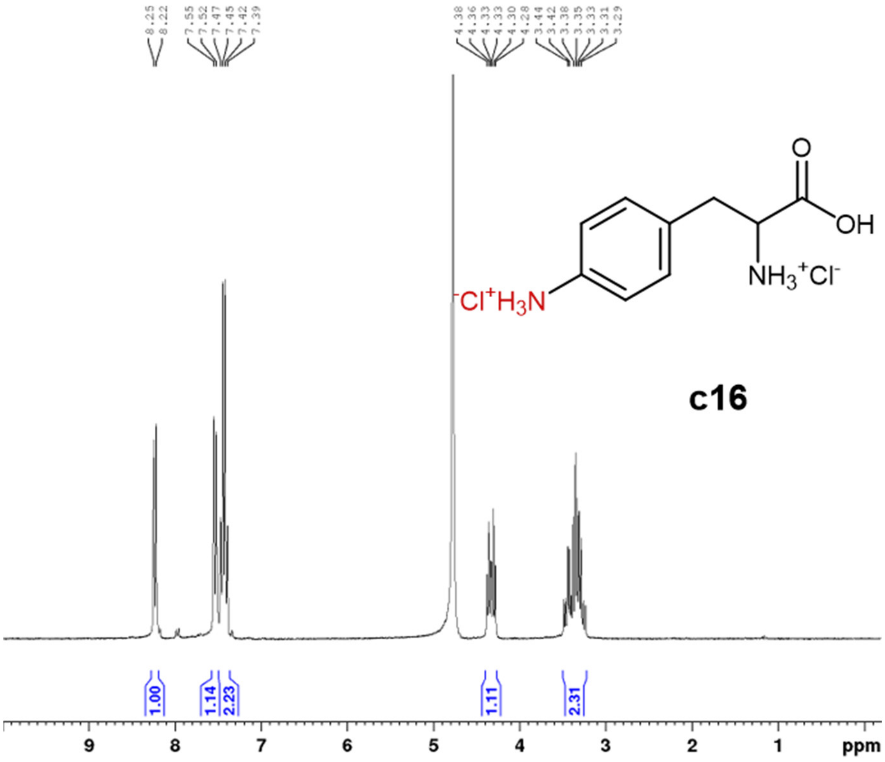

Figure S52. ^{1}H NMR of 4-(2-ammonio-2-carboxyethyl)benzenaminium chloride (**c16**) in D_2O; mixture of mono and bis salt.

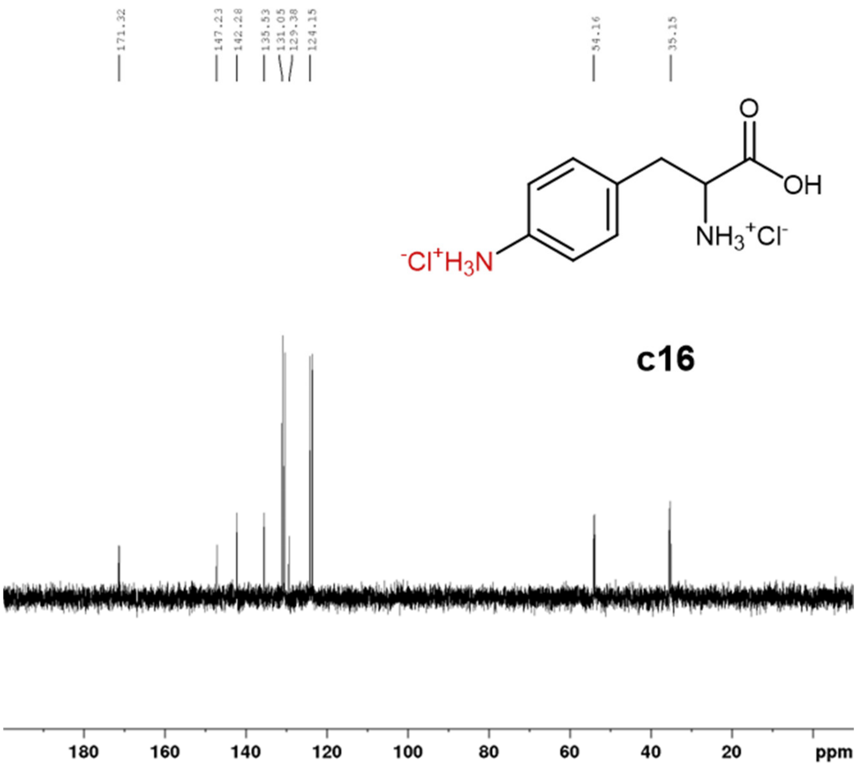

Figure S53. $^{13}C\{^{1}H\}$ NMR of 4-(2-ammonio-2-carboxyethyl)benzenaminium chloride (**c16**) in D_2O; mixture of mono and bis salt.

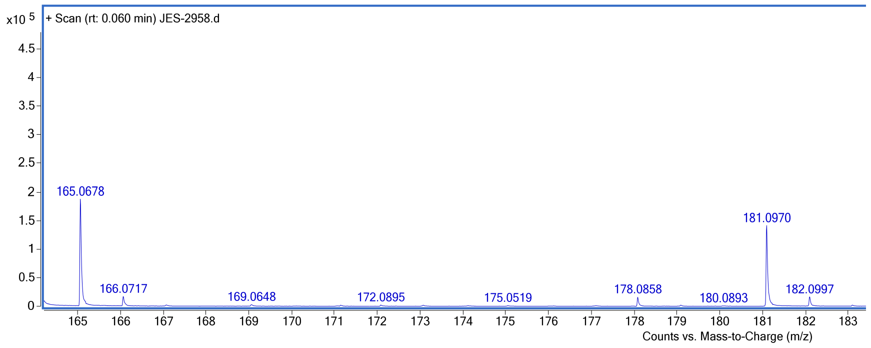

Figure S54. HR-MS of 4-(2-ammonio-2-carboxyethyl)benzenaminium chloride (**c16**).

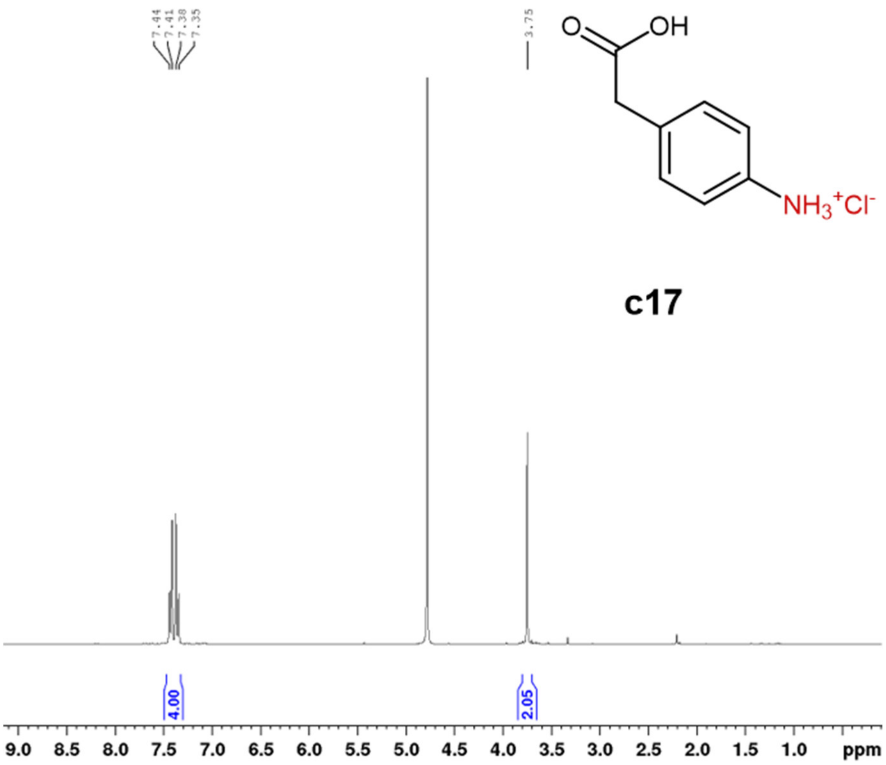

Figure S55. ^{1}H NMR of 4-(carboxymethyl)benzenaminium chloride (**c17**) in D_2O.

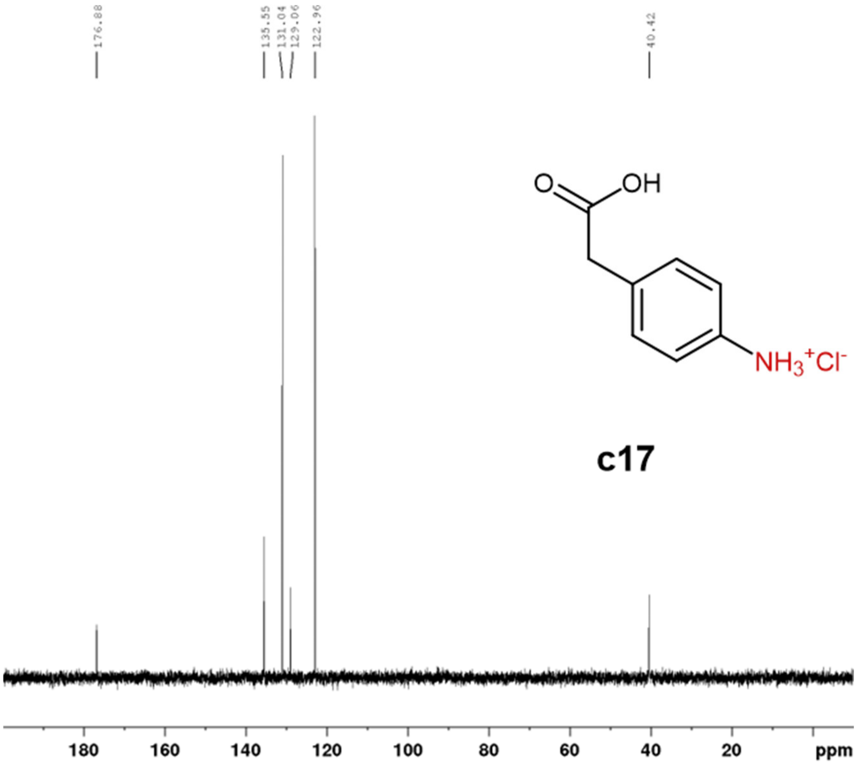

Figure S56. $^{13}C\{^{1}H\}$ NMR of 4-(carboxymethyl)benzenaminium chloride (**c17**) in D_2O.

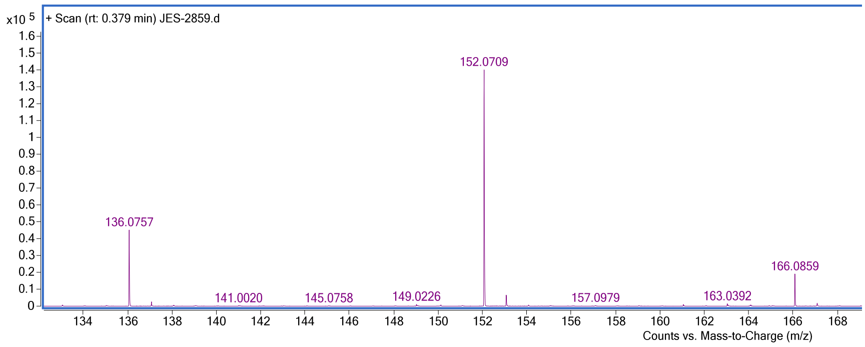

Figure S57. HR-MS of 4-(carboxymethyl)benzenaminium chloride (**c17**).

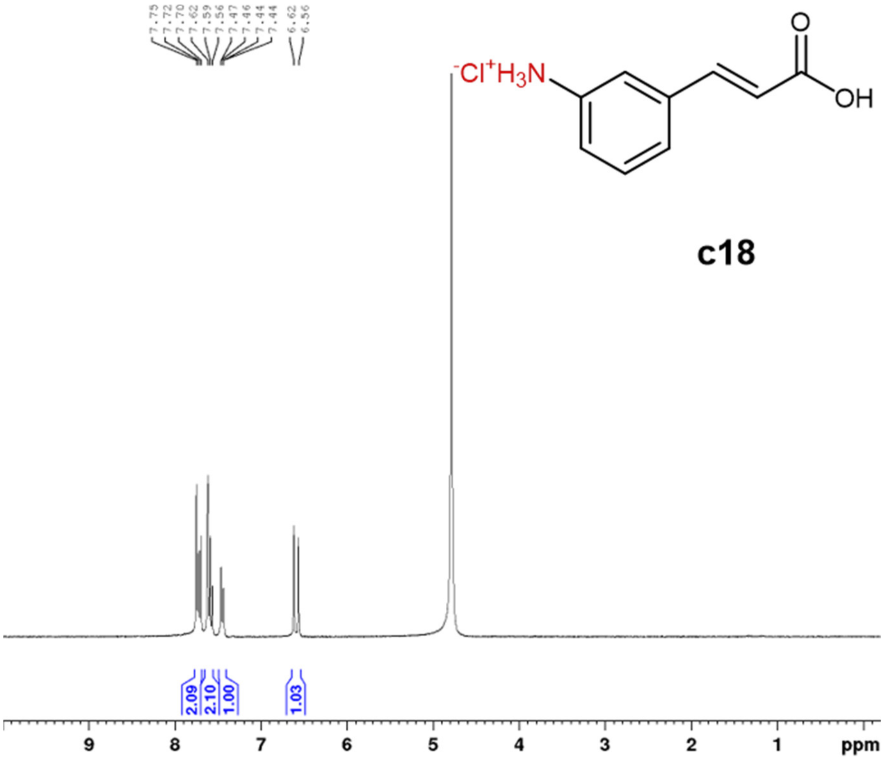

Figure S58. ^{1}H NMR of (*E*)-3-(2-carboxyvinyl)benzenaminium chloride (**c18**) in D_2O.

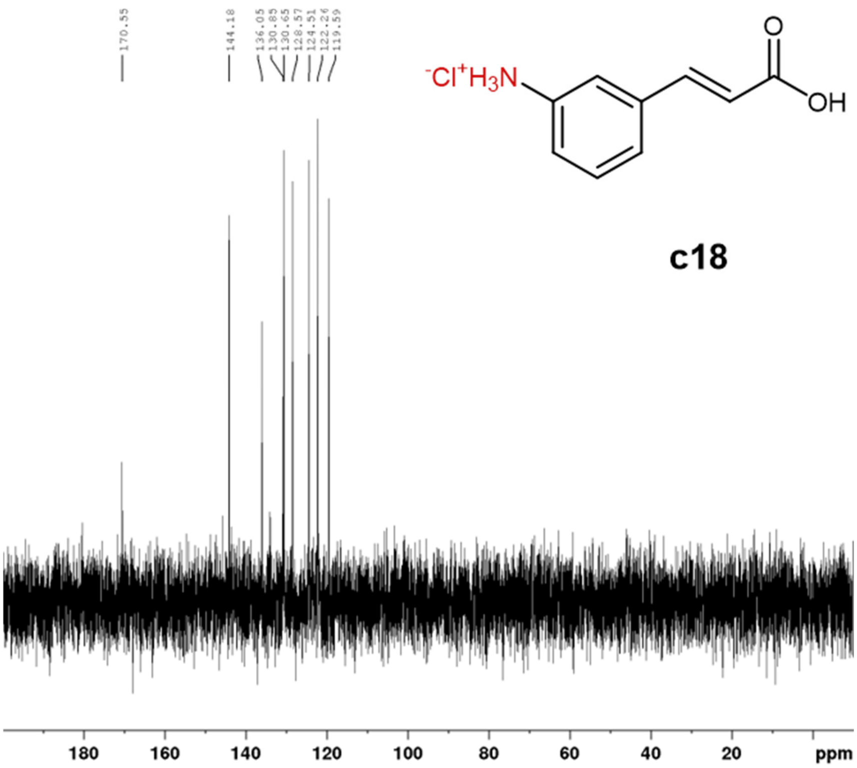

Figure S59. $^{13}C\{^{1}H\}$ NMR of (*E*)-3-(2-carboxyvinyl)benzenaminium chloride (**c18**) in D_2O.

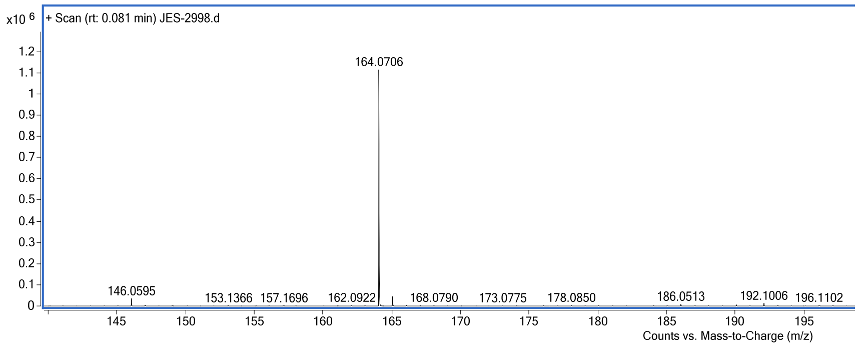

Figure S60. HR-MS of (*E*)-3-(2-carboxyvinyl)benzenaminium chloride (**c18).**

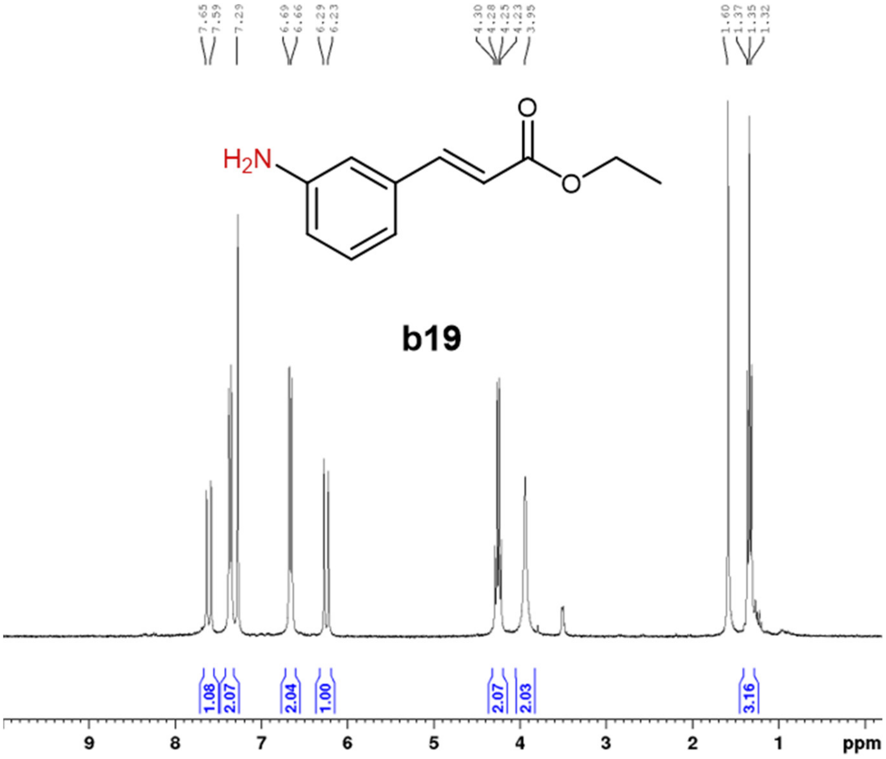

Figure S61. ^{1}H NMR of ethyl (*E*)-3-(3-aminophenyl)acrylate (**b19**) in $CDCl_3$.

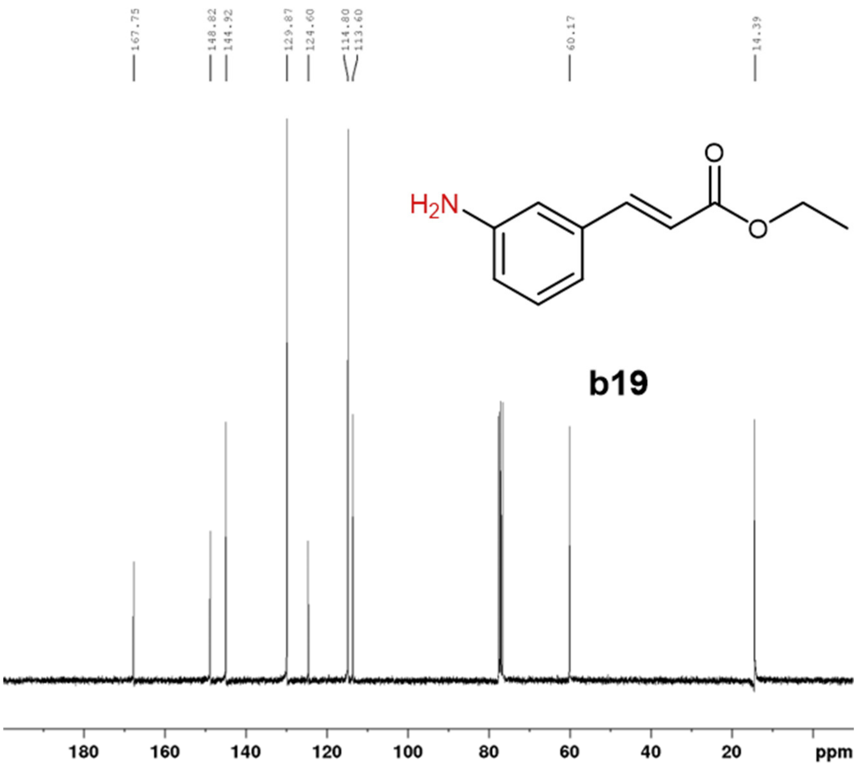

Figure S62. $^{13}C\{^{1}H\}$ NMR of ethyl (*E*)-3-(3-aminophenyl)acrylate (**b19**) in $CDCl_3$.

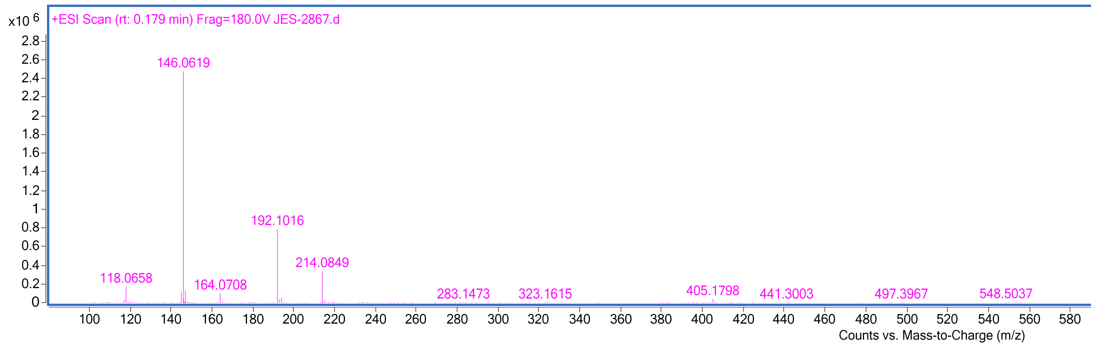

Figure S63. HR-MS of ethyl (*E*)-3-(3-aminophenyl)acrylate (**b19**).

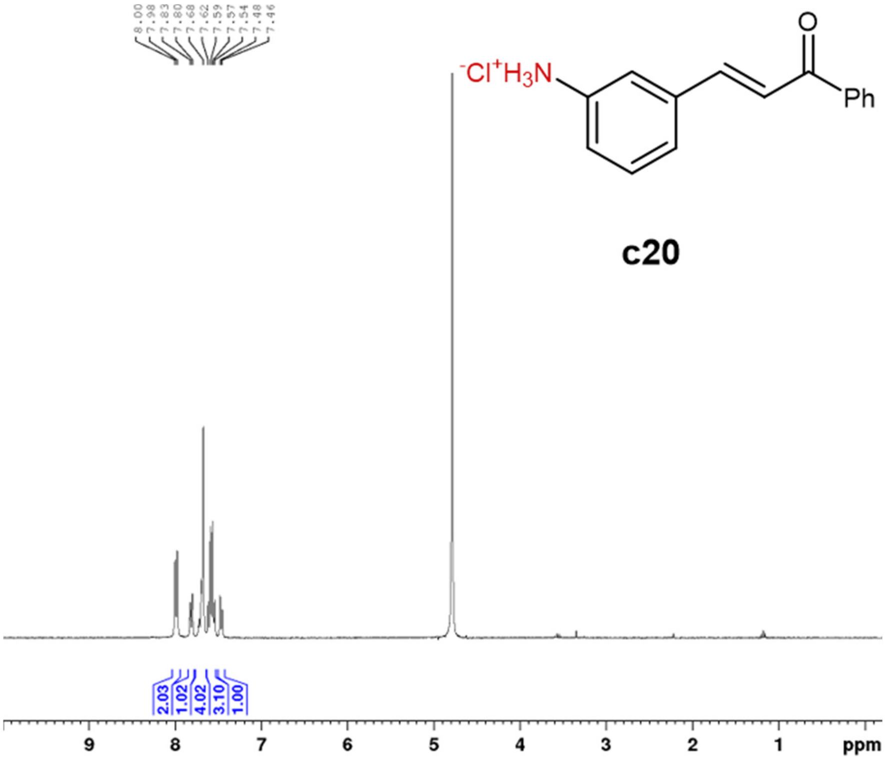

Figure S64. ¹H NMR of (*E*)-3-(3-oxo-3-phenylprop-1-en-1-yl)benzenaminium chloride (**c20**) in D_2O.

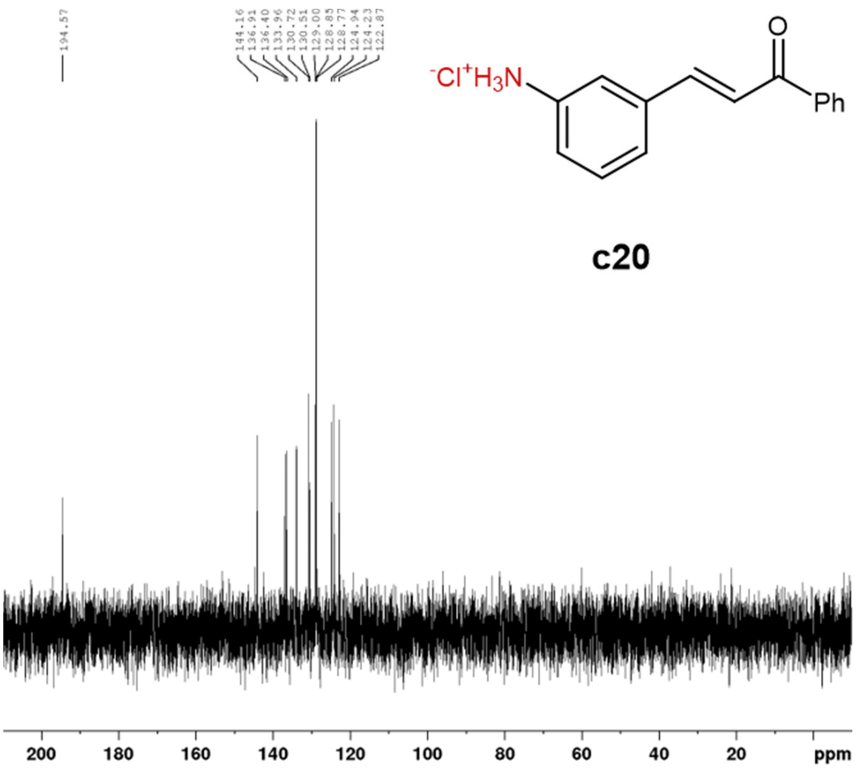

Figure S65. $^{13}C\{^{1}H\}$ NMR of (*E*)-3-(3-oxo-3-phenylprop-1-en-1-yl)benzenaminium chloride (**c20**) in D_2O.

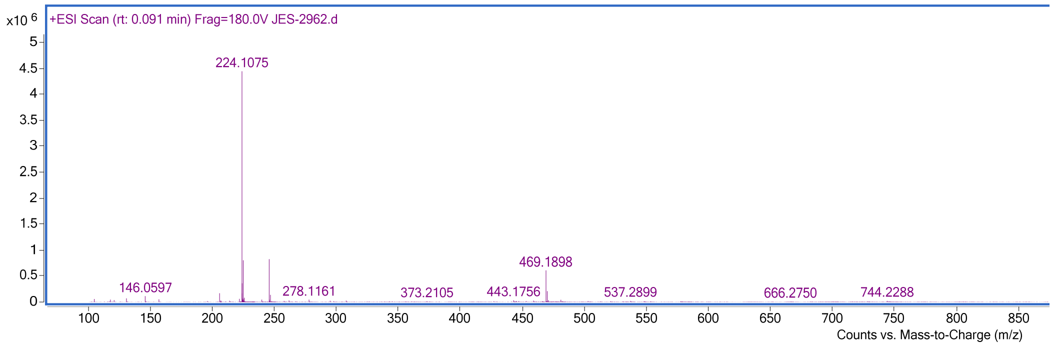

Figure S66. HR-MS of (*E*)-3-(3-oxo-3-phenylprop-1-en-1-yl)benzenaminium chloride (**c20).**

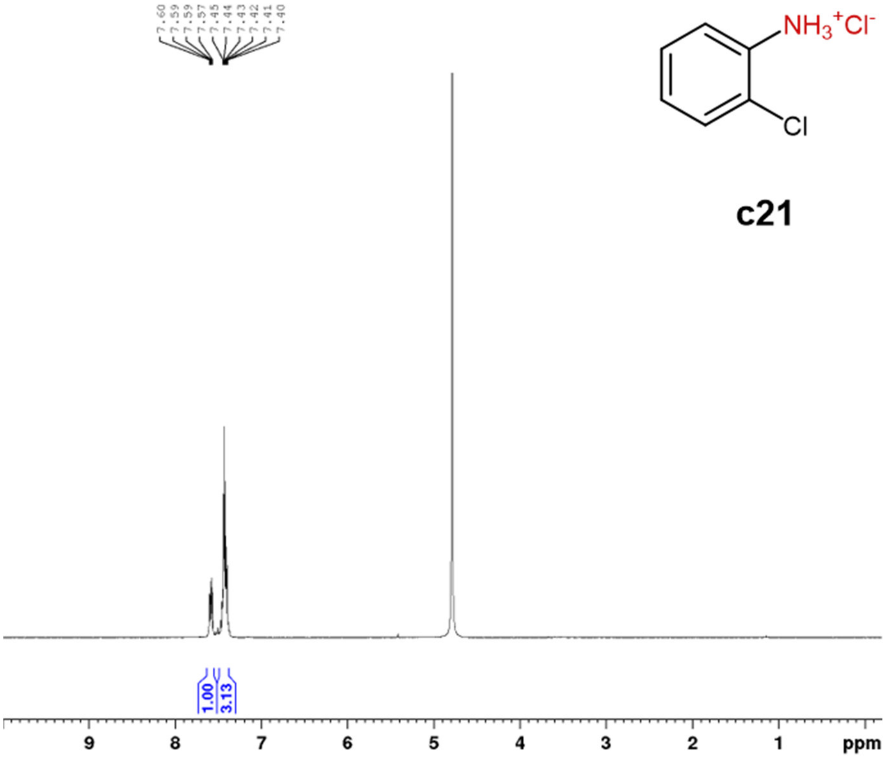

Figure S67. ^{1}H NMR of 2-chloroanilinium chloride (**c21**) in D_2O.

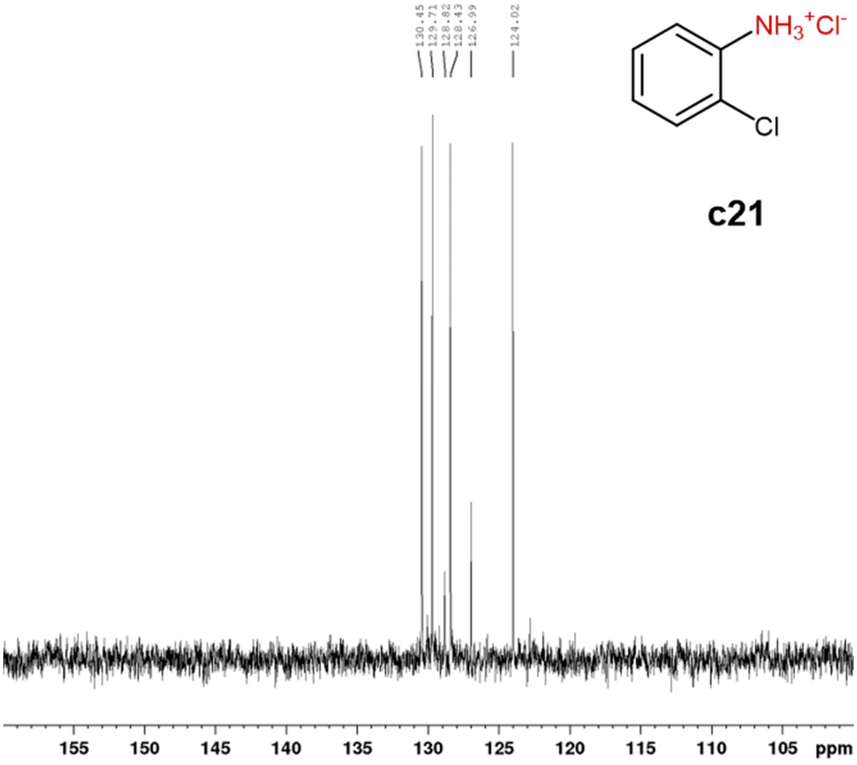

Figure S68. $^{13}C\{^{1}H\}$ NMR of 2-chloroanilinium chloride (**c21**) in D_2O.

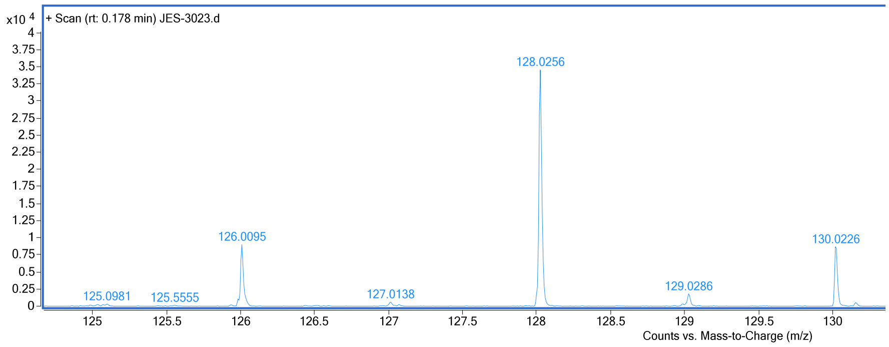

Figure S69. HR-MS of 2-chloroanilinium chloride (**c21**).

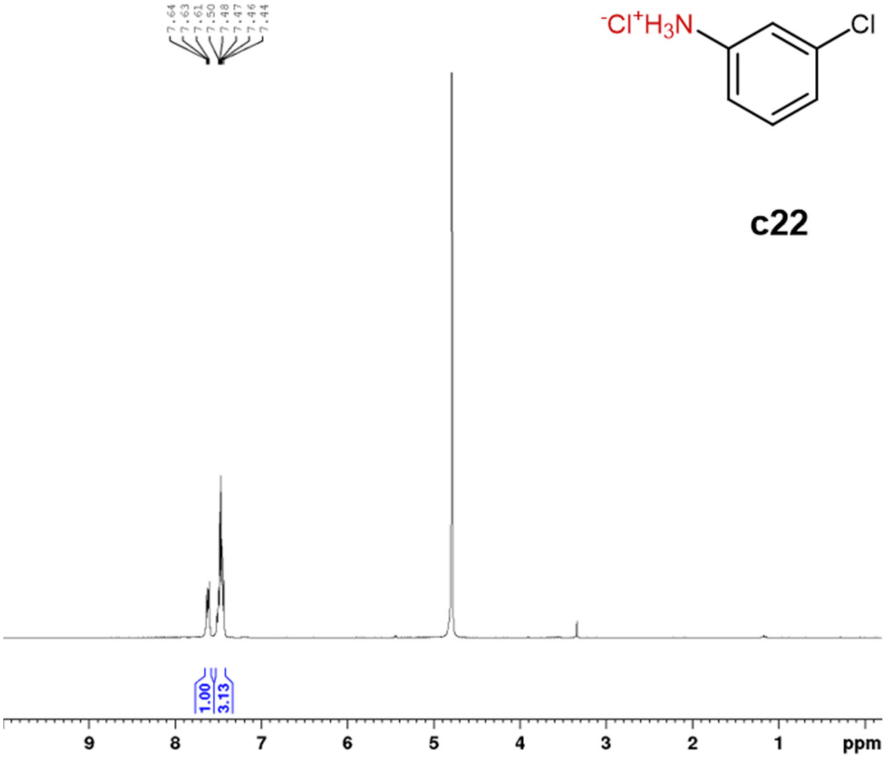

Figure S70. ^{1}H NMR of 3-chloroanilinium chloride (**c22**) in D_2O.

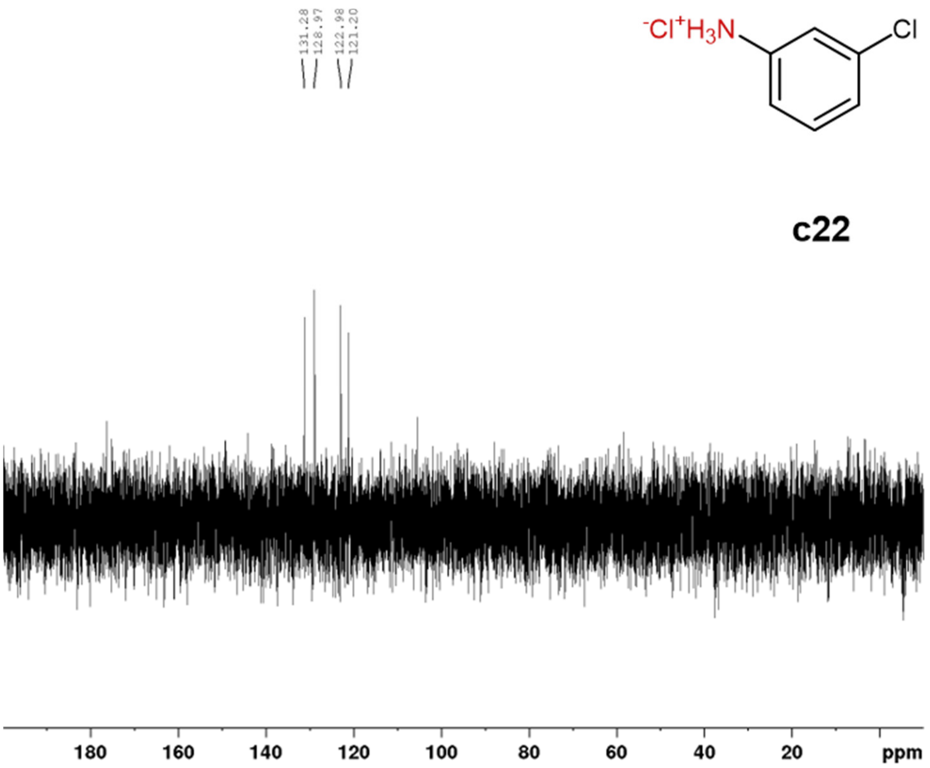

Figure S71. $^{13}C\{^1H\}$ NMR of 3-chloroanilinium chloride (**c22**) in D_2O.

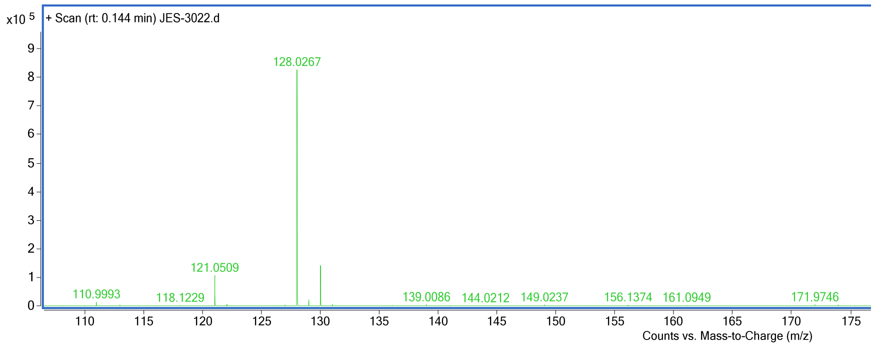

Figure S72. HR-MS of 3-chloroanilinium chloride (**c22**).

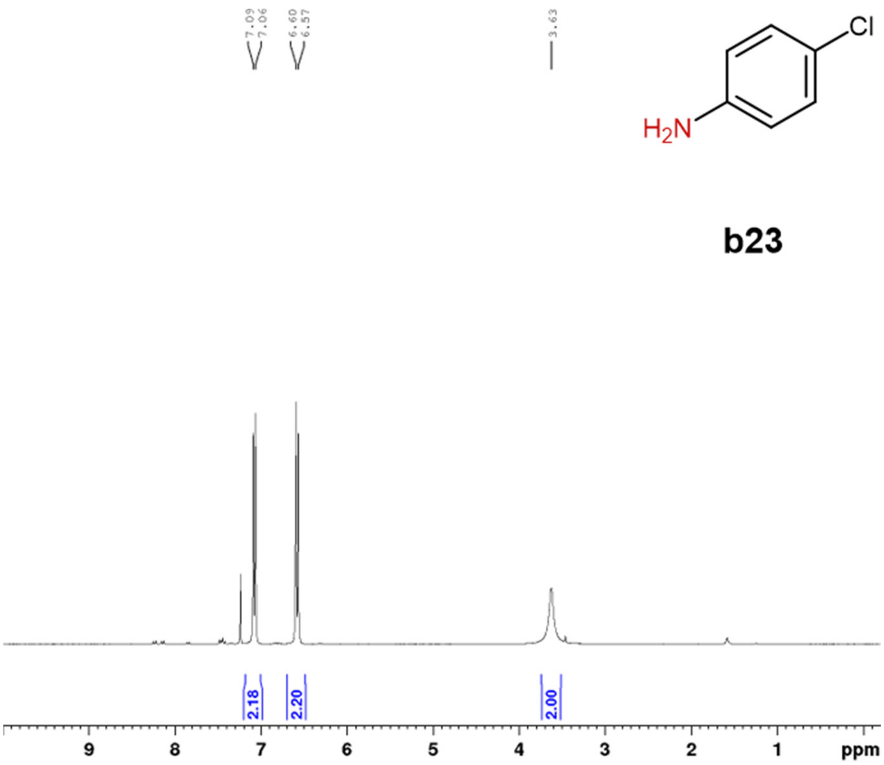

Figure S73. ^{1}H NMR of 4-chloroaniline (**b23**) in $CDCl_3$.

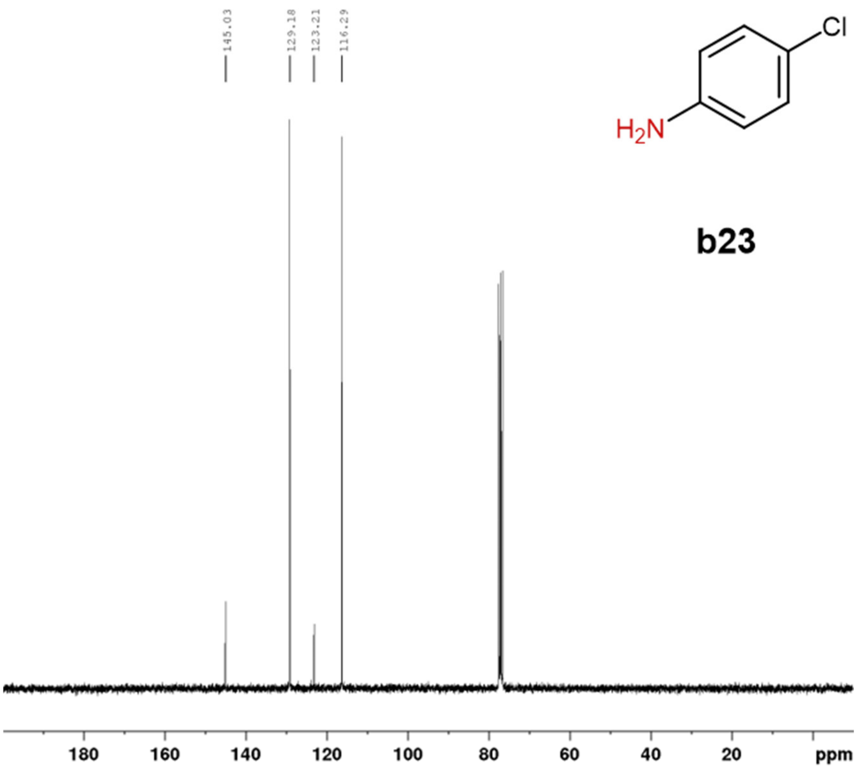

Figure S74. $^{13}C\{^{1}H\}$NMR of 4-chloroaniline (**b23**) in $CDCl_3$.

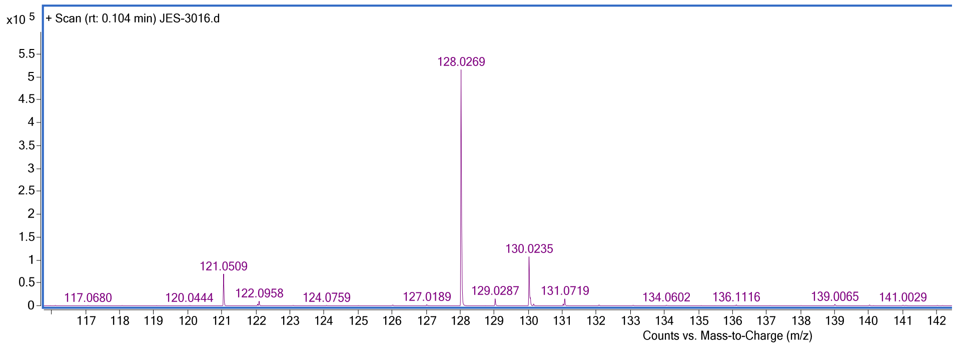

Figure S75. HR-MS of 4-chloroaniline (**b23**).

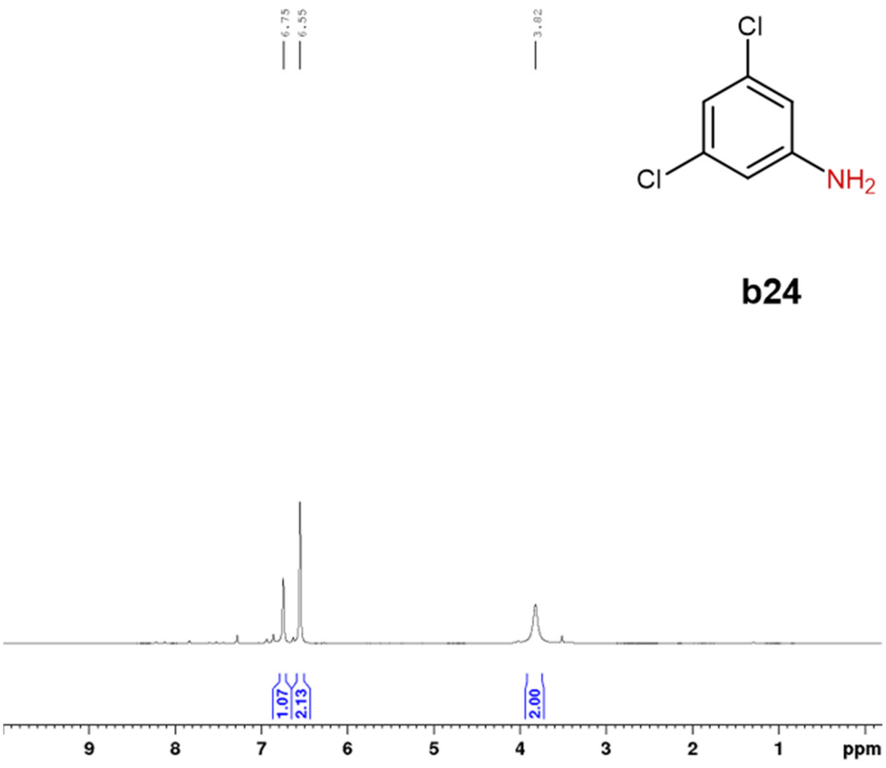

Figure S76. ^{1}H NMR of 3,5-dichloroaniline (**b24**) in $CDCl_3$.

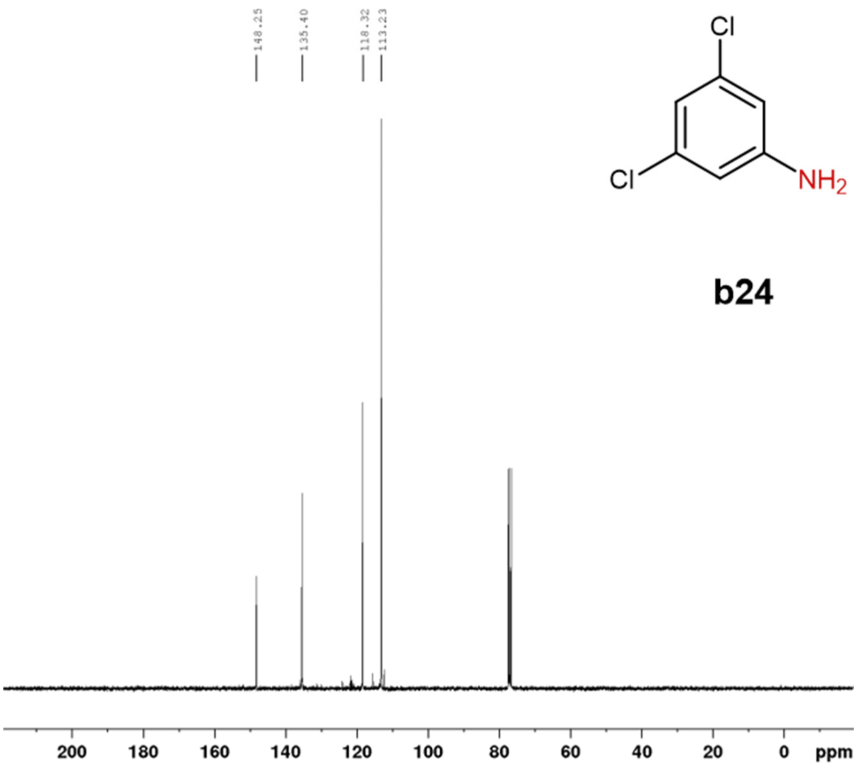

Figure S77. $^{13}C\{^{1}H\}$NMR of 3,5-dichloroaniline (**b24**) in $CDCl_3$.

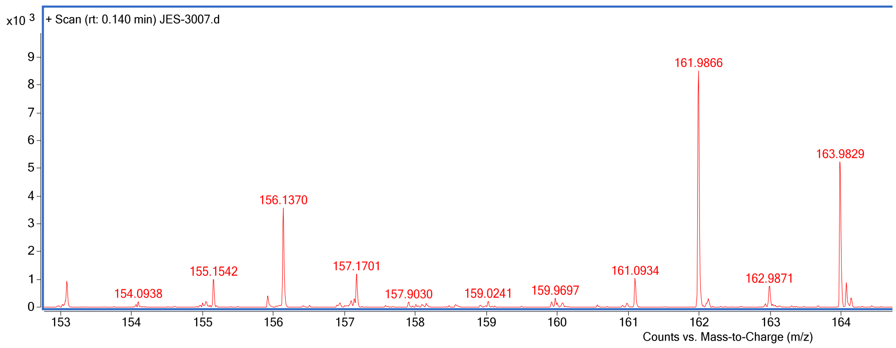

Figure S78. HR-MS of 3,5-dichloroaniline (**b24**).

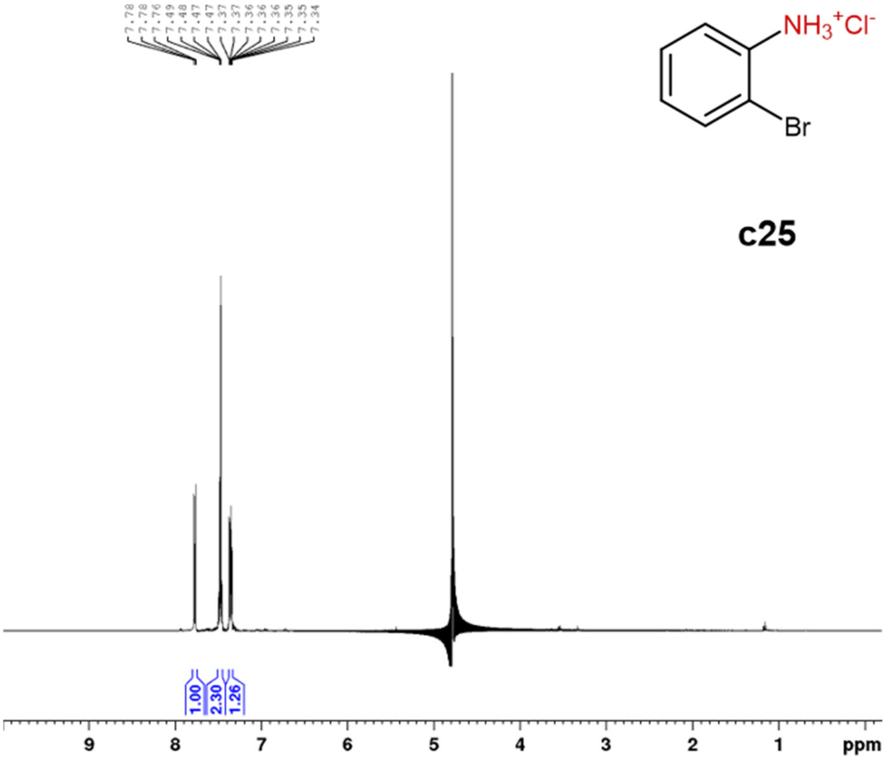

Figure S79. ^{1}H NMR of 2-bromoanilinium chloride (**c25**) in D_2O.

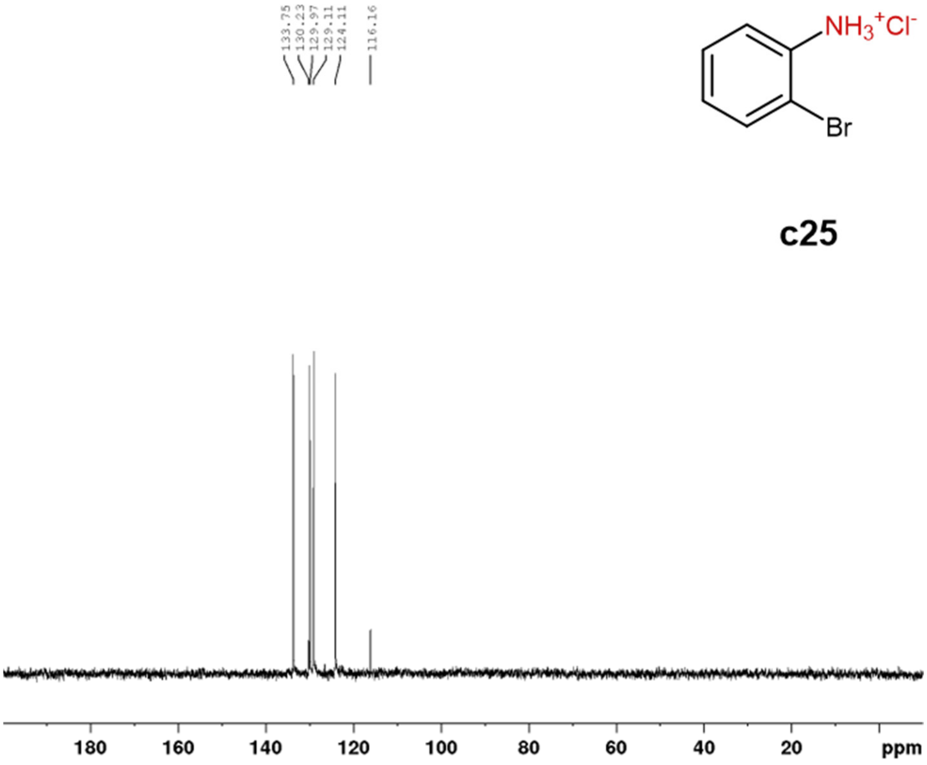

Figure S80. $^{13}C\{^{1}H\}$NMR of 2-bromoanilinium chloride (**c25**) in D_2O.

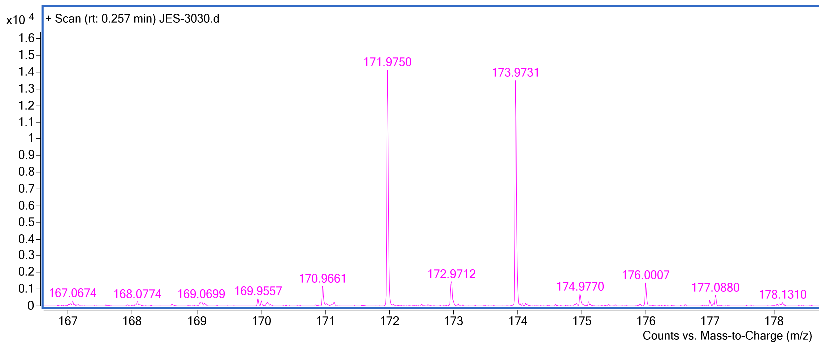

Figure S81. HR-MS of 2-bromoanilinium chloride (**c25**).

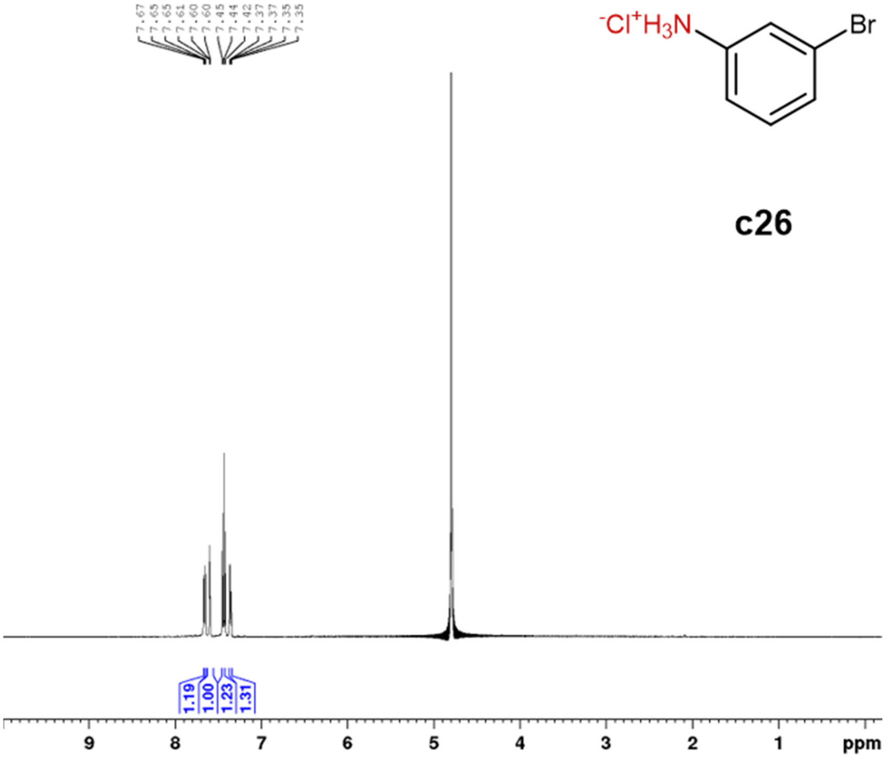

Figure S82. ^{1}H NMR of 3-bromoanilinium chloride (**c26**) in D_2O.

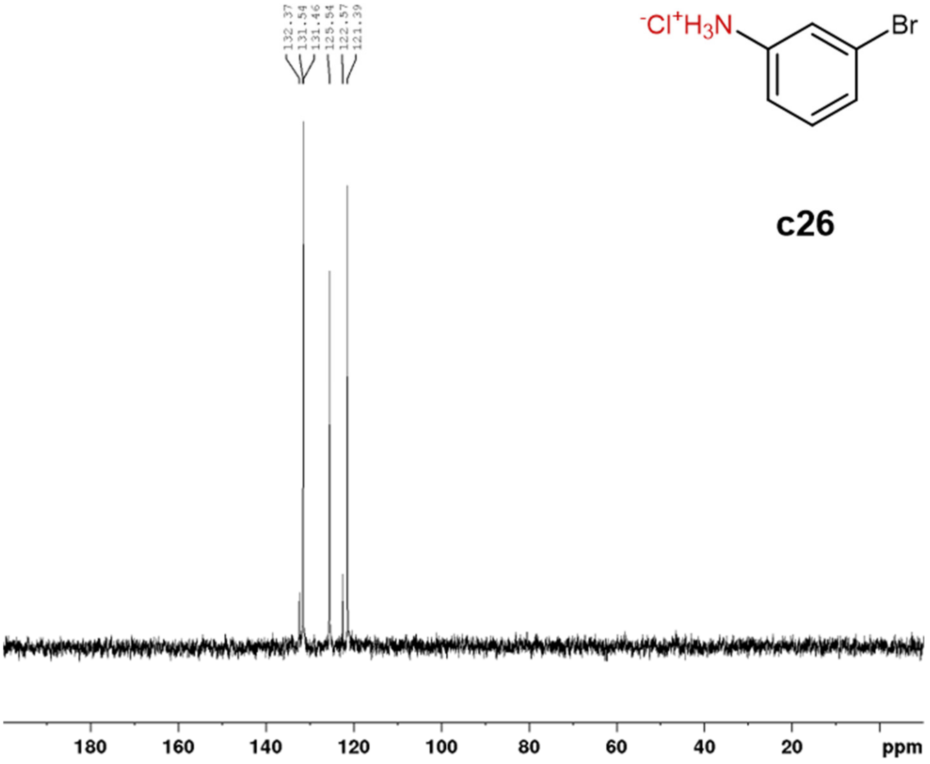

Figure S83. $^{13}C\{^{1}H\}$NMR of 3-bromoanilinium chloride (**c26**) in D_2O.

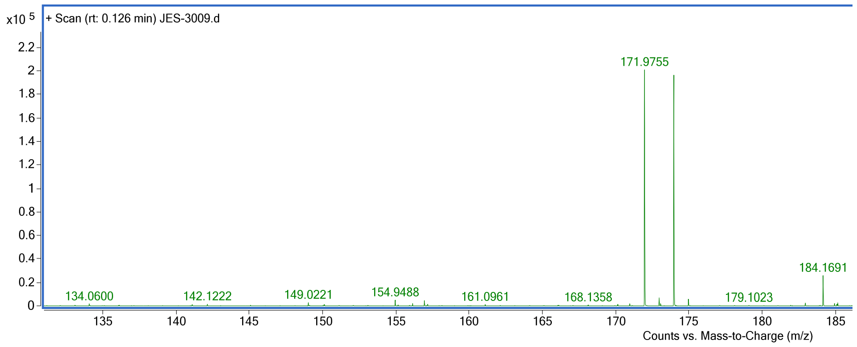

Figure S84. HR-MS of 3-bromoanilinium chloride (**c26**).

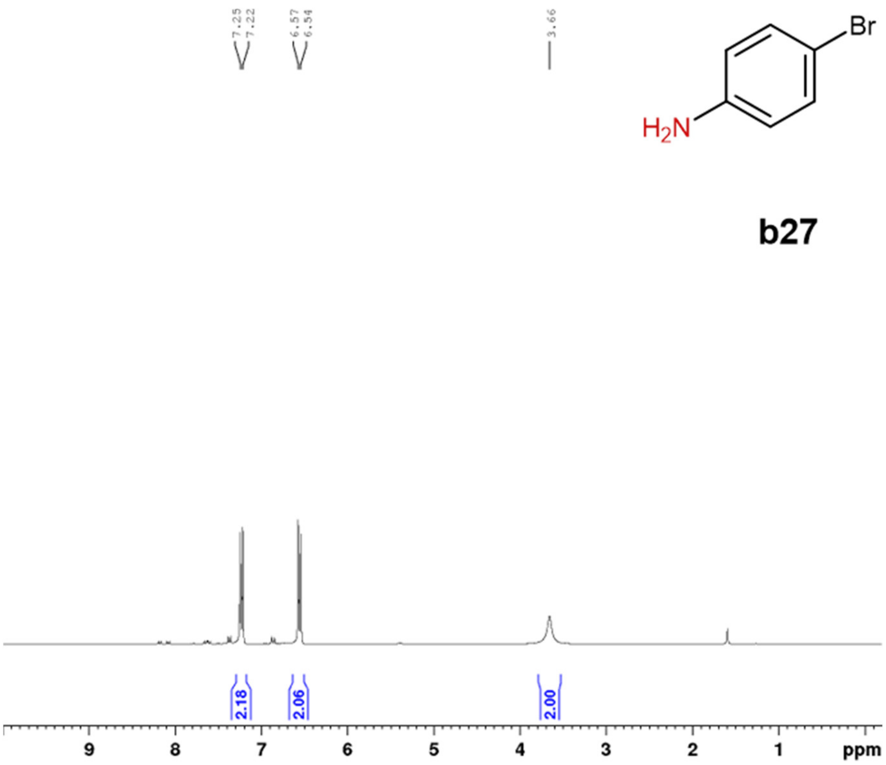

b27

Figure S85. ^{1}H NMR of 4-bromoaniline (**b27**) in $CDCl_3$.

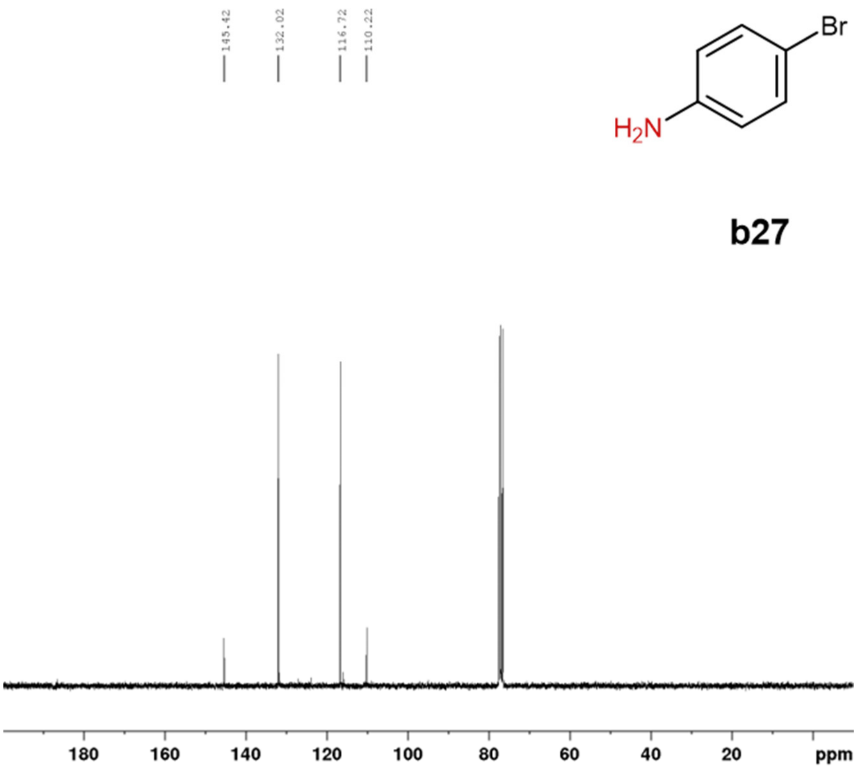

b27

Figure S86. $^{13}C\{^{1}H\}$NMR of 4-bromoaniline (**b27**) in $CDCl_3$.

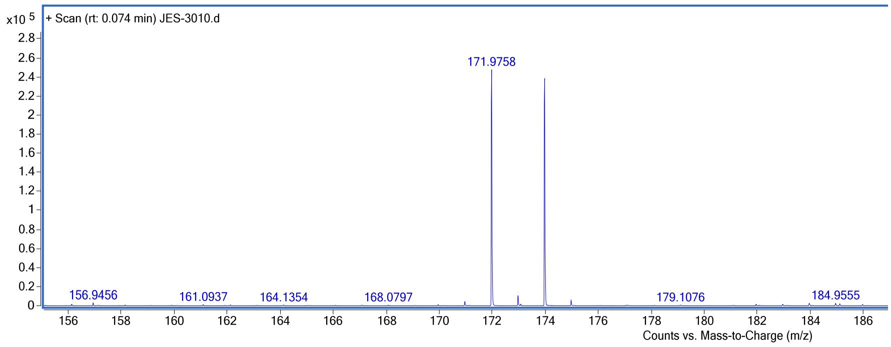

Figure S87. HR-MS of 4-bromoaniline (**b27**).

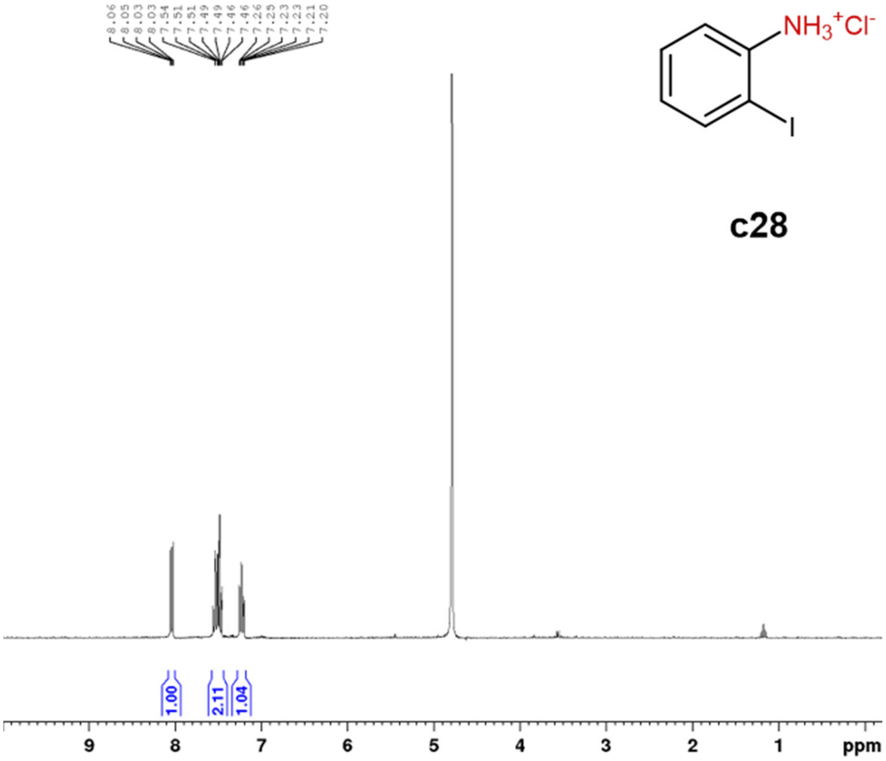

Figure S88. ^{1}H NMR of 2-iodoanilinium chloride (**c28**) in D_2O.

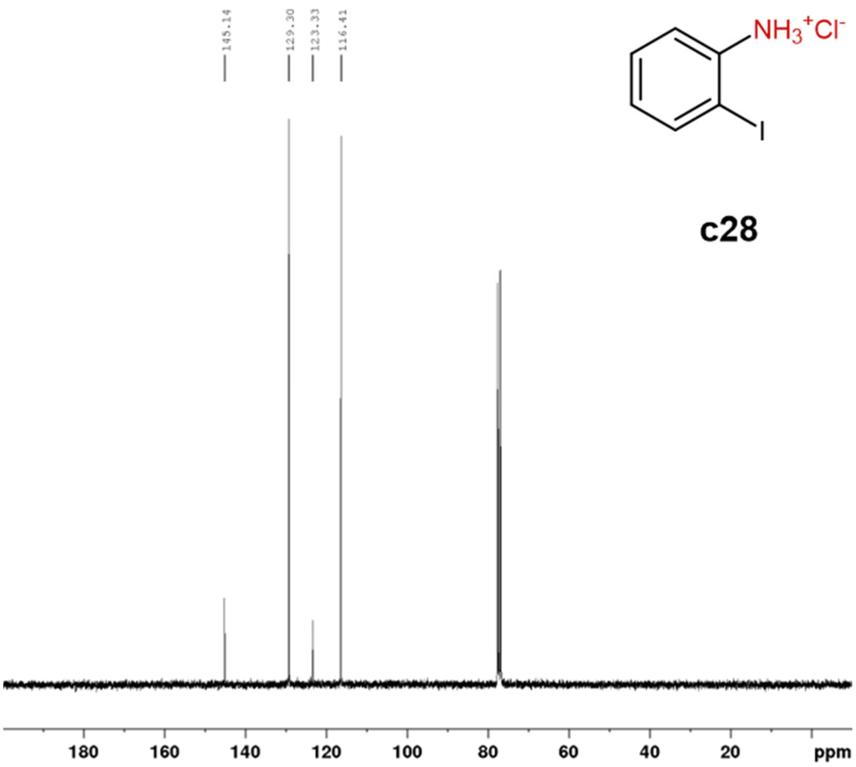

Figure S89. $^{13}C\{^1H\}$NMR of 2-iodoanilinium chloride (**c28**) in D_2O.

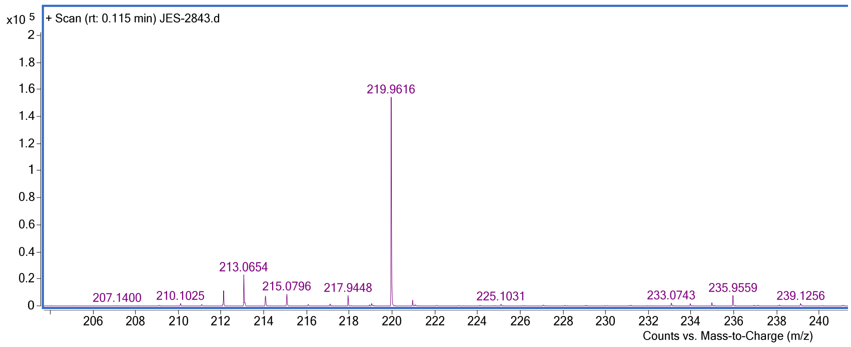

Figure S90. HR-MS of 2-iodoanilinium chloride (**c28**).

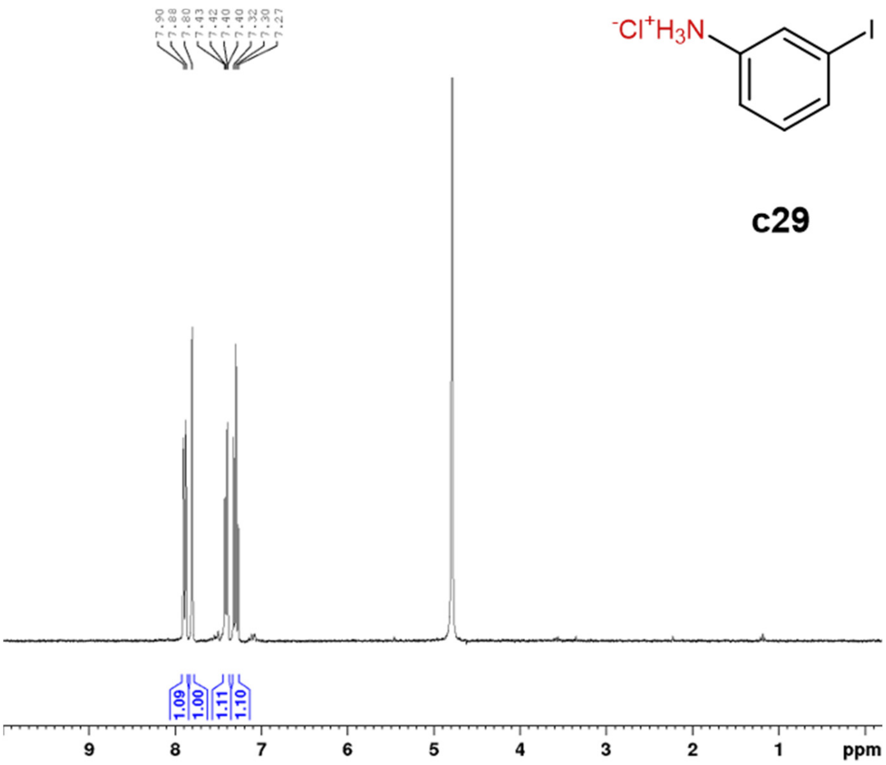

Figure S91. ^{1}H NMR of 3-iodoanilinium chloride (**c29**) in D_2O.

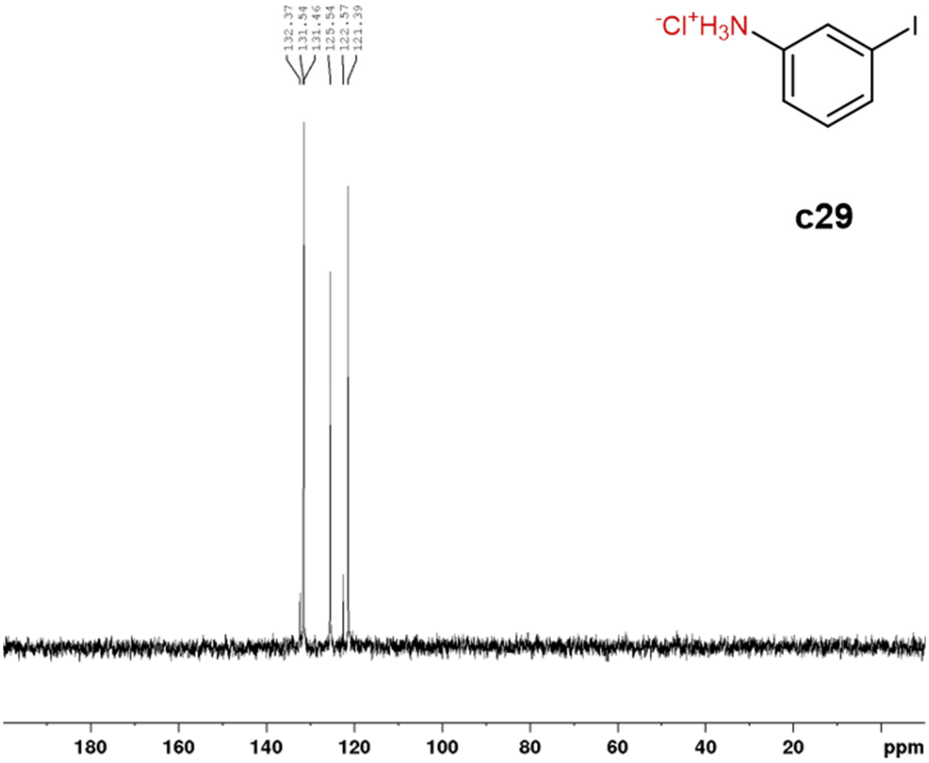

Figure S92. $^{13}C\{^{1}H\}$NMR of 3-iodoanilinium chloride (**c29**) in D_2O.

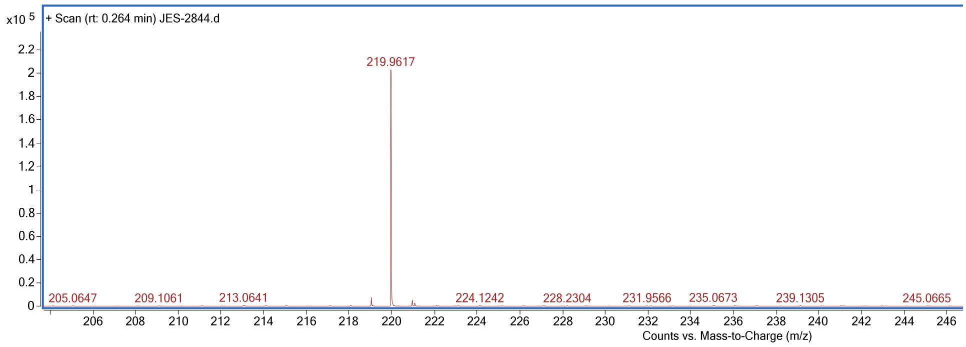

Figure S93. HR-MS of 3-iodoanilinium chloride (**c29**).

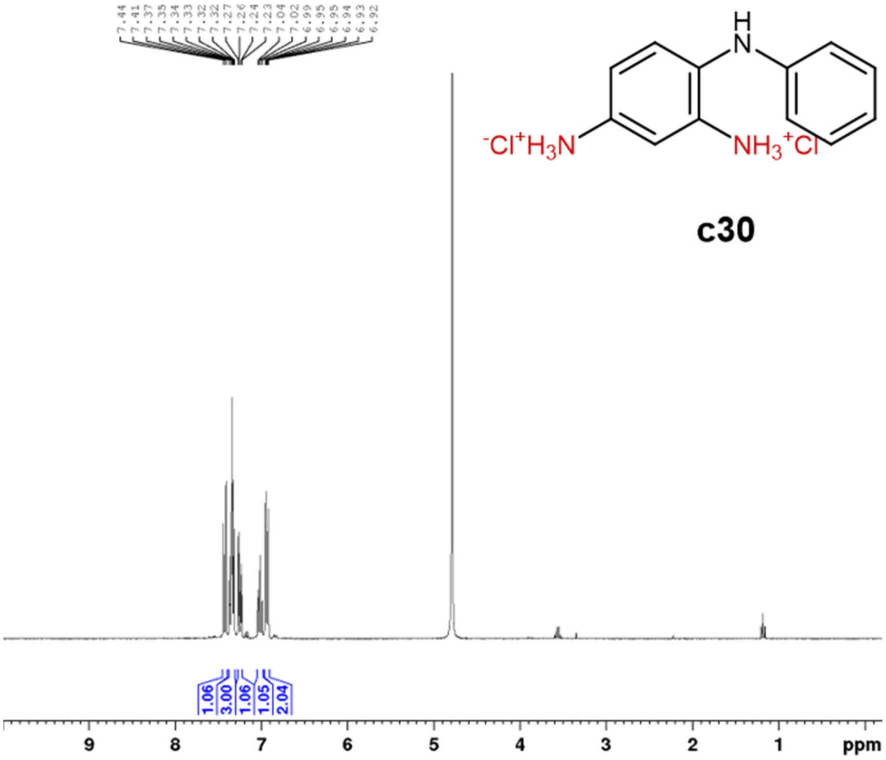

Figure S94. ^{1}H NMR of 2,4-diaminiumdiphenylaminium trichloride (**c30**) in D_2O.

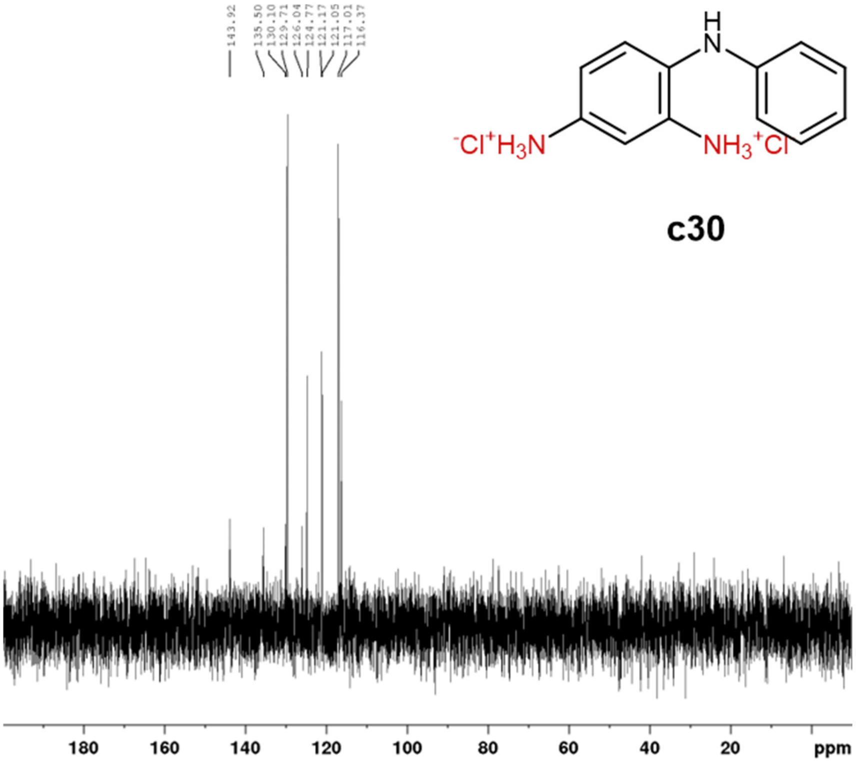

Figure S95. $^{13}C\{^1H\}$NMR of 2,4-diaminiumdiphenylaminium trichloride (**c30**) in D_2O.

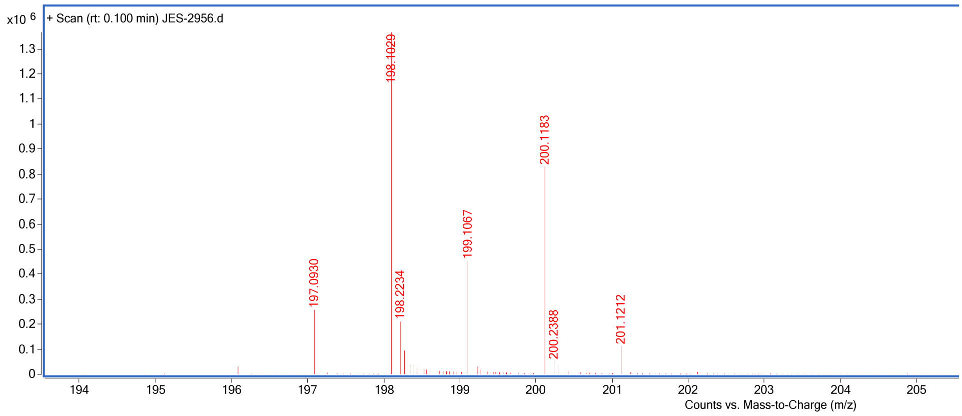

Figure S96. HR-MS of 2,4-diaminiumdiphenylaminium trichloride (**c30**).

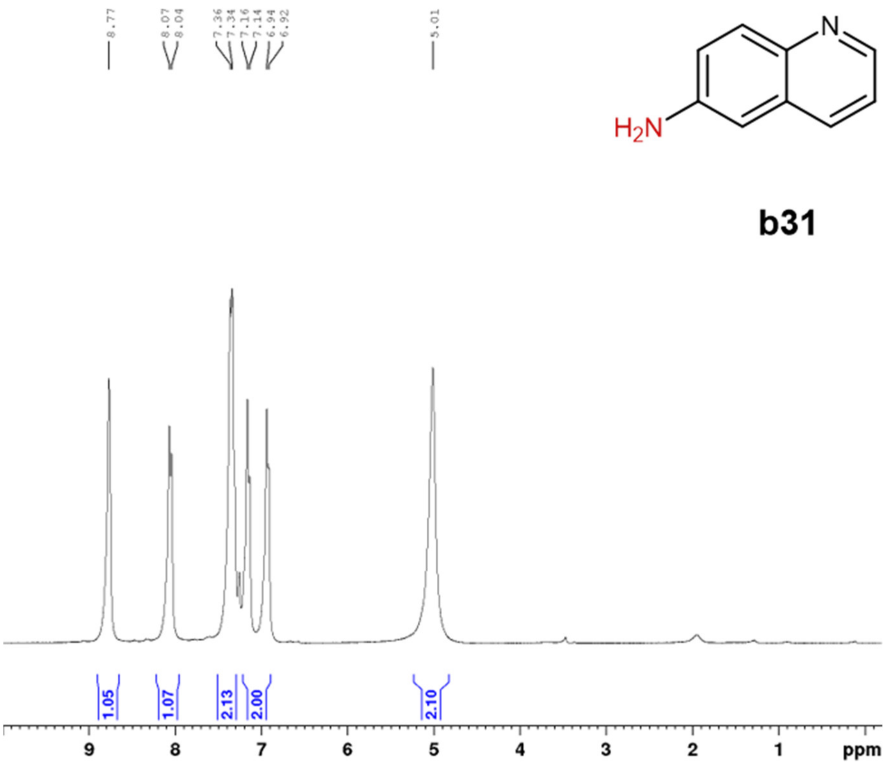

Figure S97. ^{1}H NMR of 6-aminoquinoline (**b31**) in $CDCl_3$.

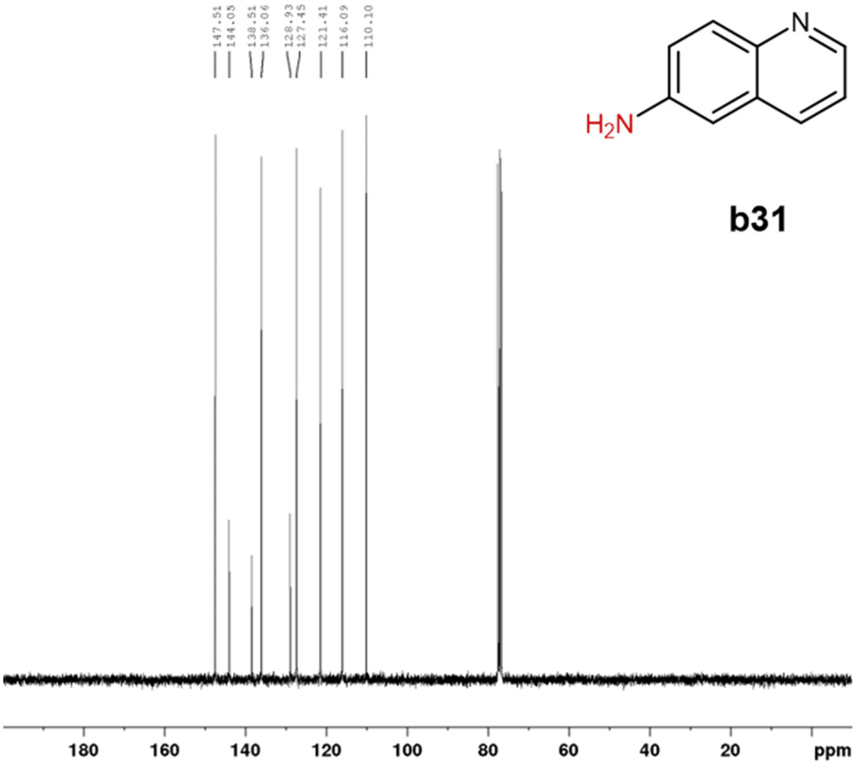

Figure S98. $^{13}C\{^{1}H\}$NMR of 6-aminoquinoline (**b31**) in $CDCl_3$.

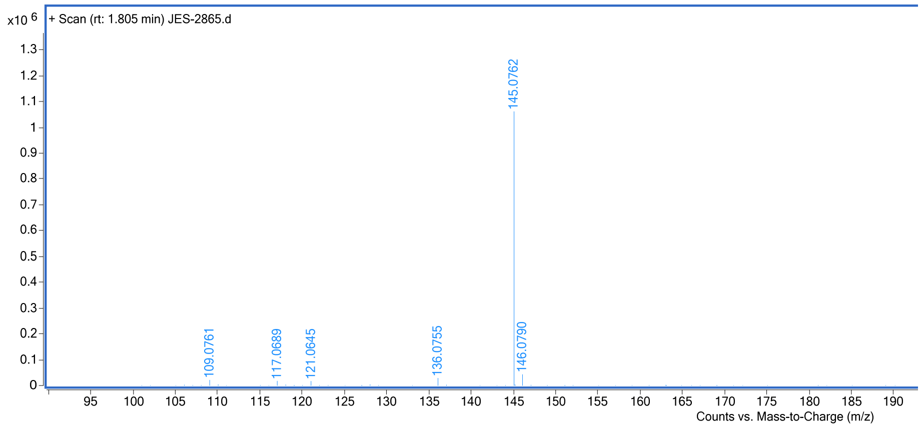

Figure S99. HR-MS of 6-aminoquinoline (**b31**).

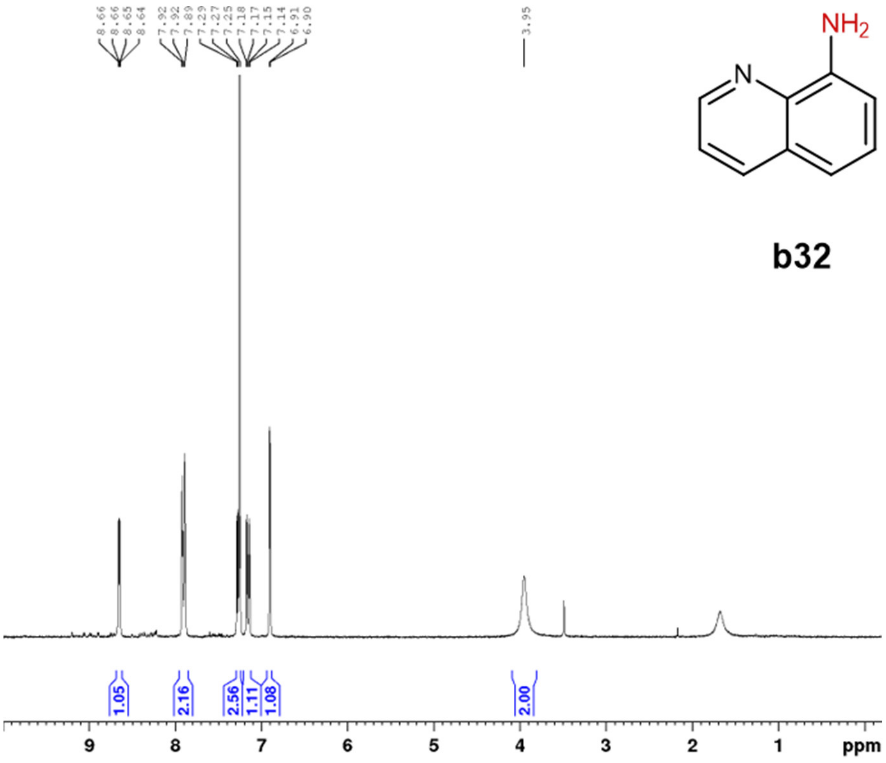

Figure S100. ^{1}H NMR of 8-aminoquinoline (**b32**) in $CDCl_3$.

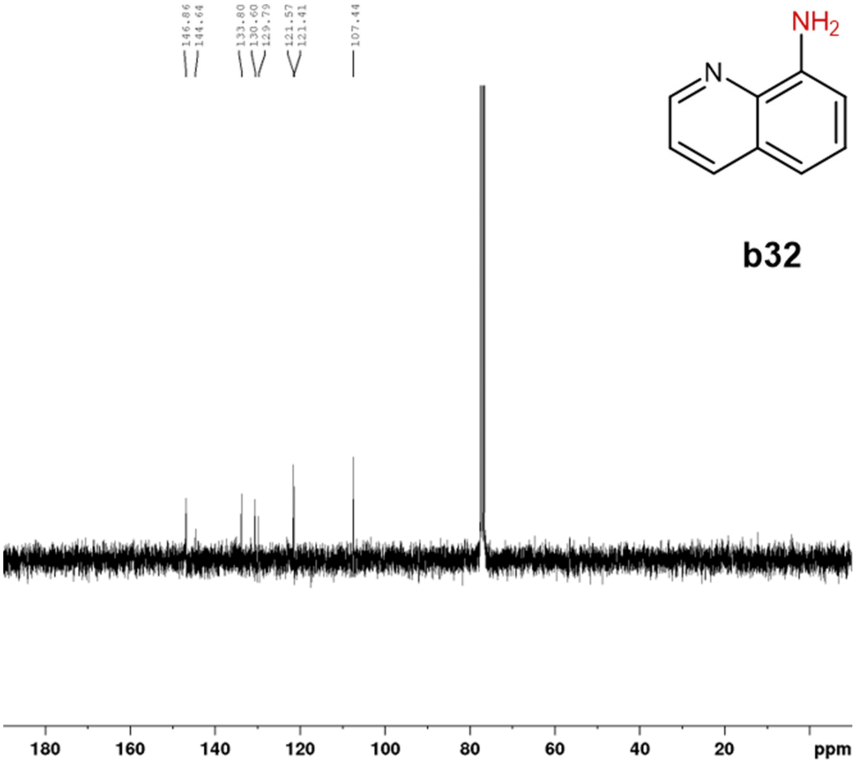

Figure S101. $^{13}C\{^{1}H\}$NMR of 8-aminoquinoline (**b32**) in $CDCl_3$.

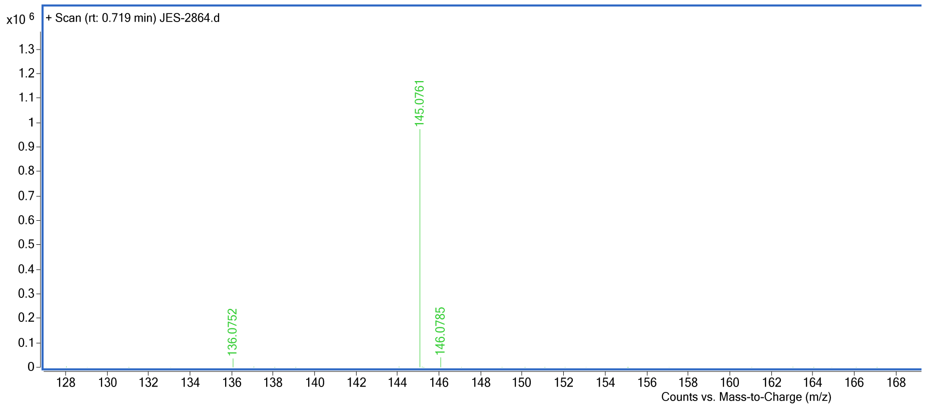

Figure S102. HR-MS of 8-aminoquinoline (**b32**).

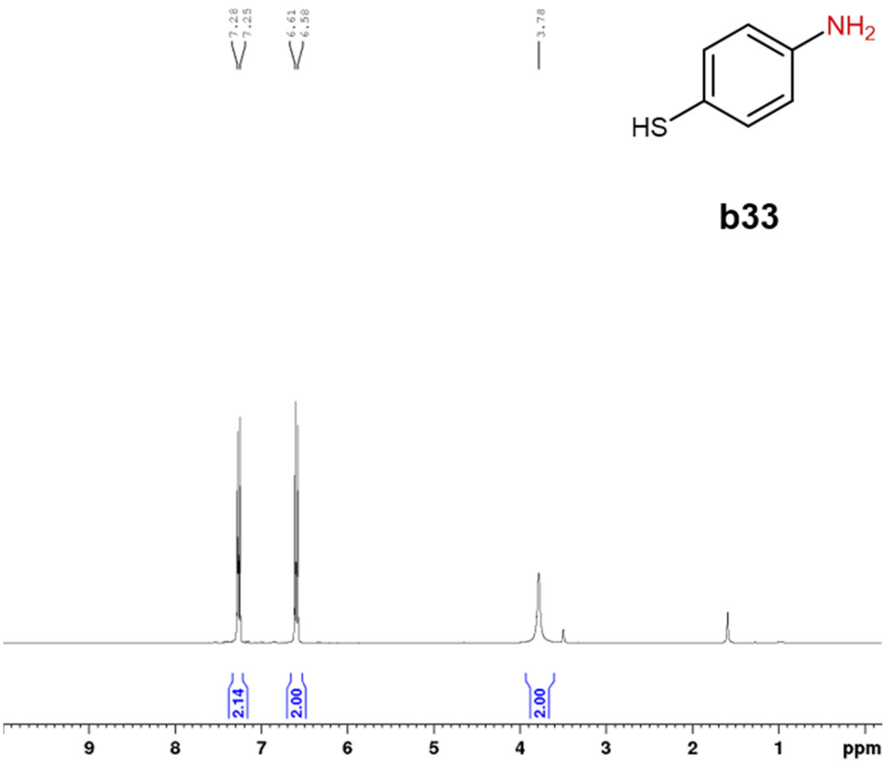

Figure S103. ^{1}H NMR of 4-aminothiophenol (**b33**) in $CDCl_3$.

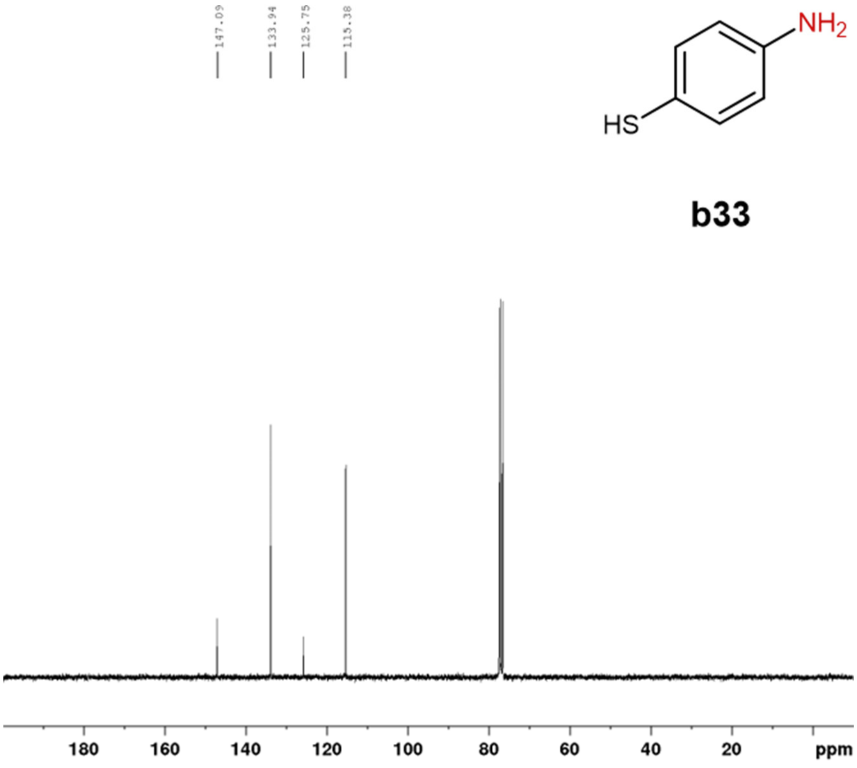

Figure S104. $^{13}C\{^1H\}$NMR of 4-aminothiophenol (**b33**) in $CDCl_3$.

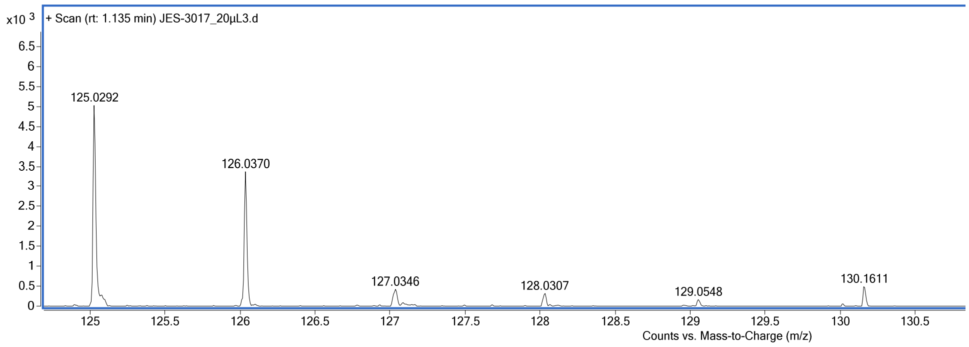

Figure S105. HR-MS of 4-aminothiophenol (**b33**).

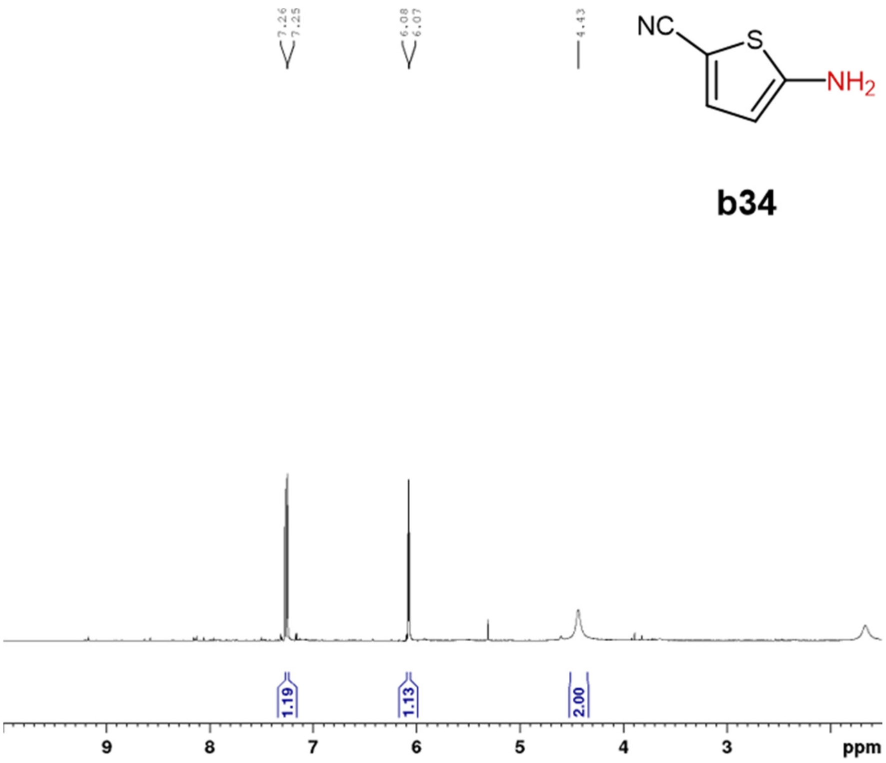

Figure S106. ^{1}H NMR of 5-aminothiophene-2-carbonitrile (**b34**) in $CDCl_3$.

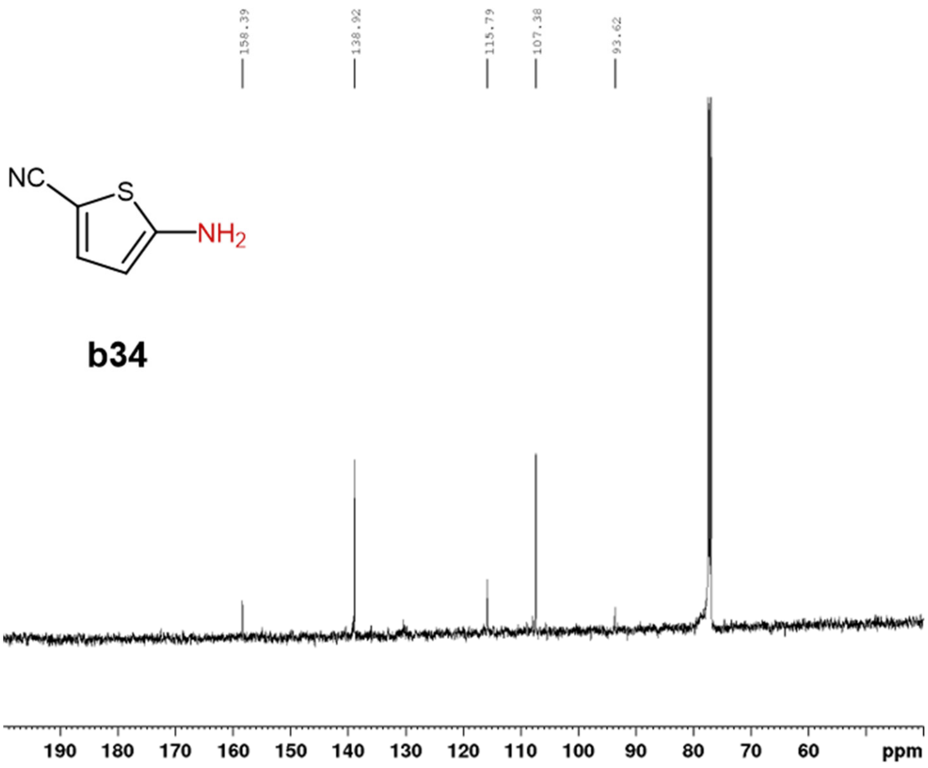

Figure S107. $^{13}C\{^1H\}$NMR of 5-aminothiophene-2-carbonitrile (**b34**) in $CDCl_3$.

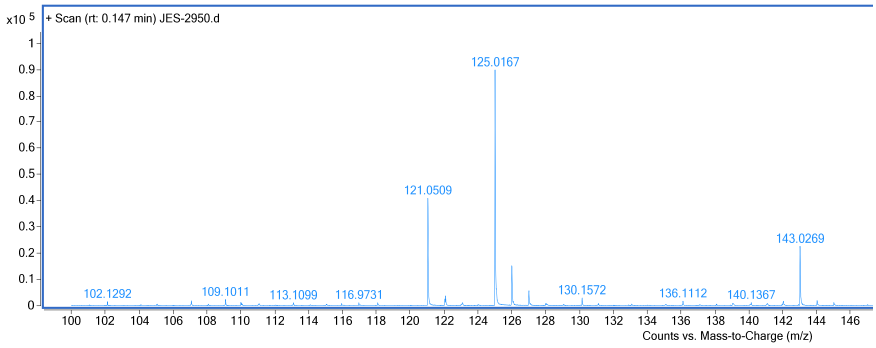

Figure S108. HR-MS of 5-aminothiophene-2-carbonitrile (**b34**).

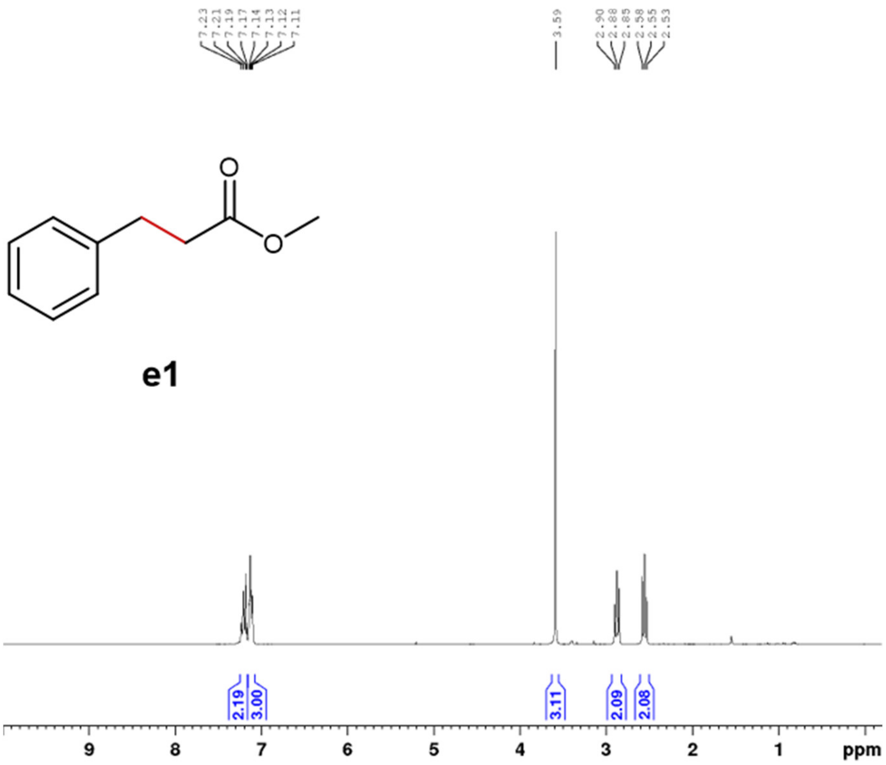

Figure S109. ^{1}H NMR of methyl 3-phenylpropanoate (**e1**) in $CDCl_3$.

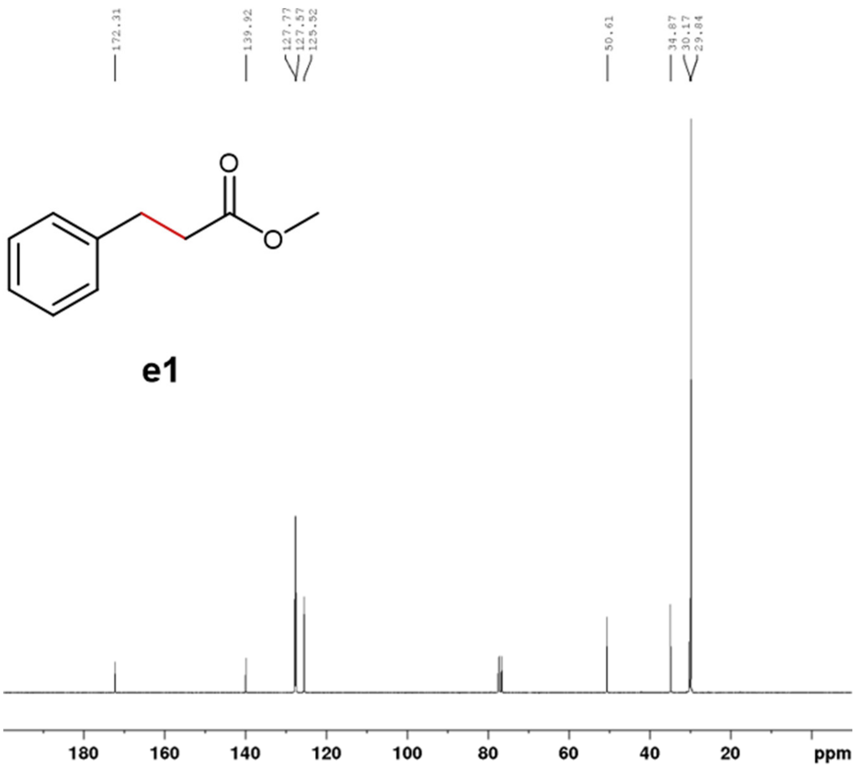

Figure S110. $^{13}C\{^{1}H\}$NMR of methyl 3-phenylpropanoate (**e1**) in $CDCl_3$.

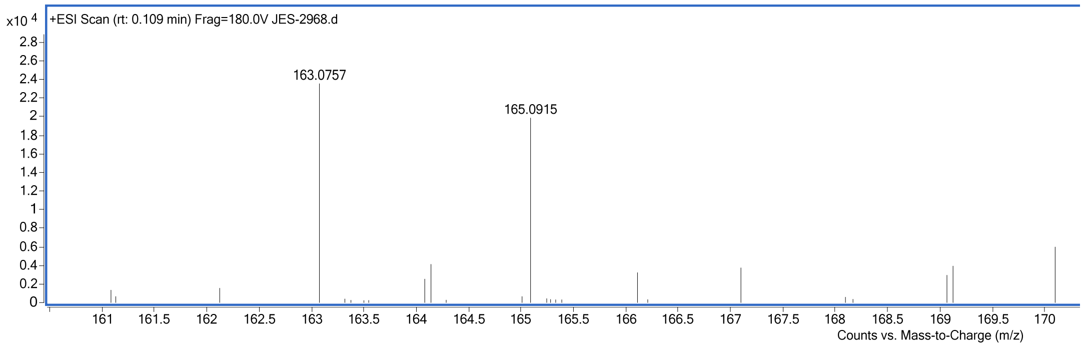

Figure S111. HR-MS of methyl 3-phenylpropanoate (**e1**).

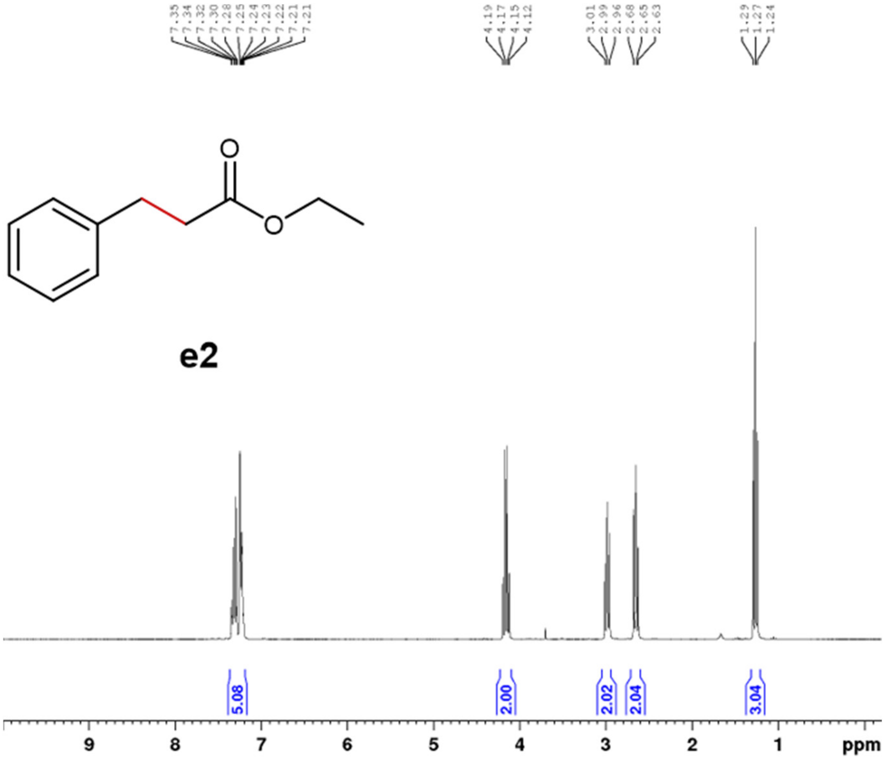

Figure S112. ^{1}H NMR of ethyl 3-phenylpropanoate (**e2**) in $CDCl_3$.

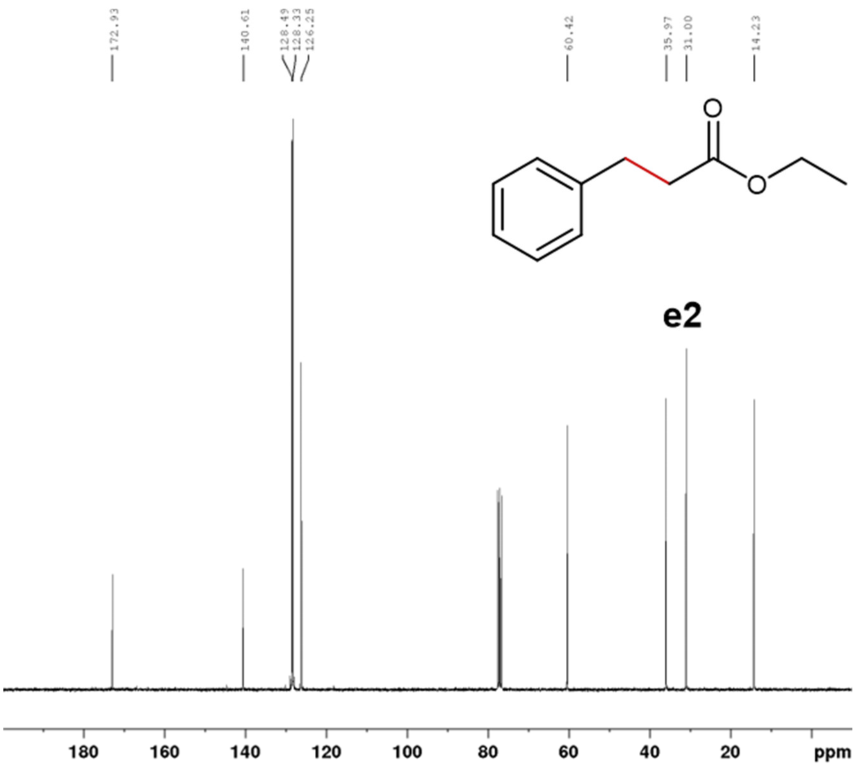

Figure S113. $^{13}C\{^{1}H\}$NMR of ethyl 3-phenylpropanoate (**e2**) in $CDCl_3$.

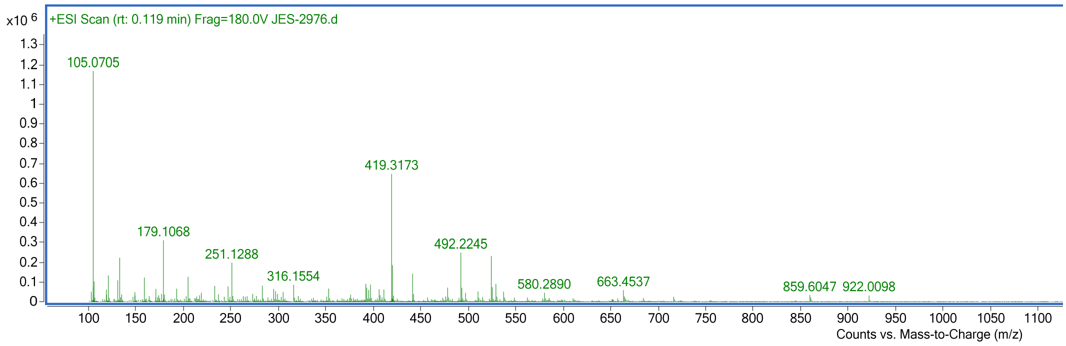

Figure S114. HR-MS of ethyl 3-phenylpropanoate (**e2**).

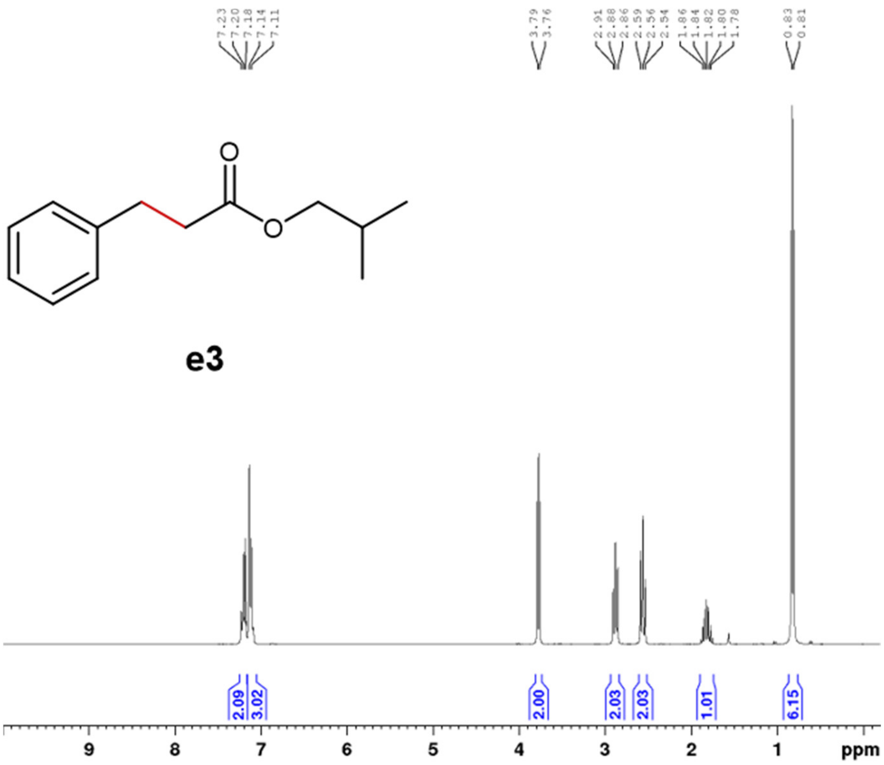

Figure S115. ^{1}H NMR of isobutyl 3-phenylpropanoate (**e3**) in $CDCl_3$.

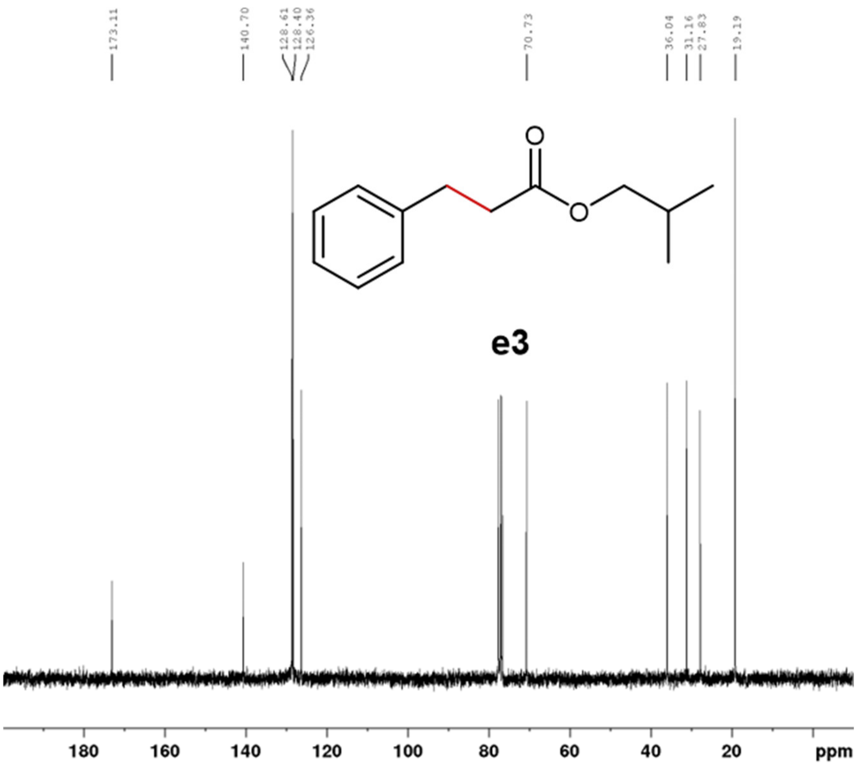

Figure S116. $^{13}C\{^{1}H\}$NMR of isobutyl 3-phenylpropanoate (**e3**) in $CDCl_3$.

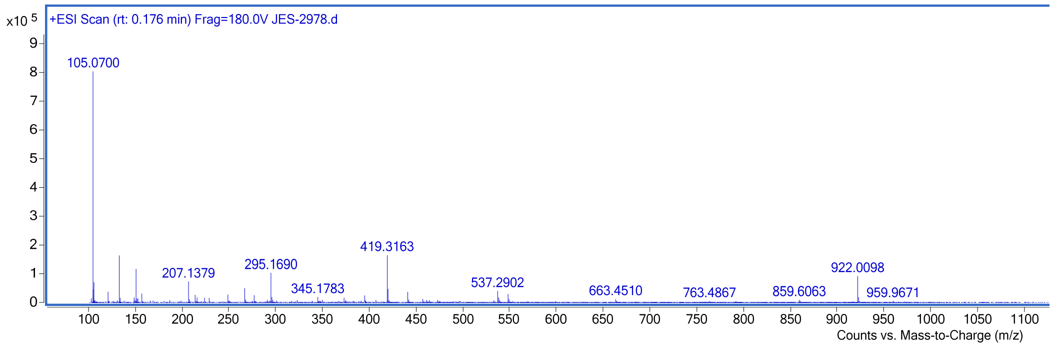

Figure S117. HR-MS of isobutyl 3-phenylpropanoate (**e3**).

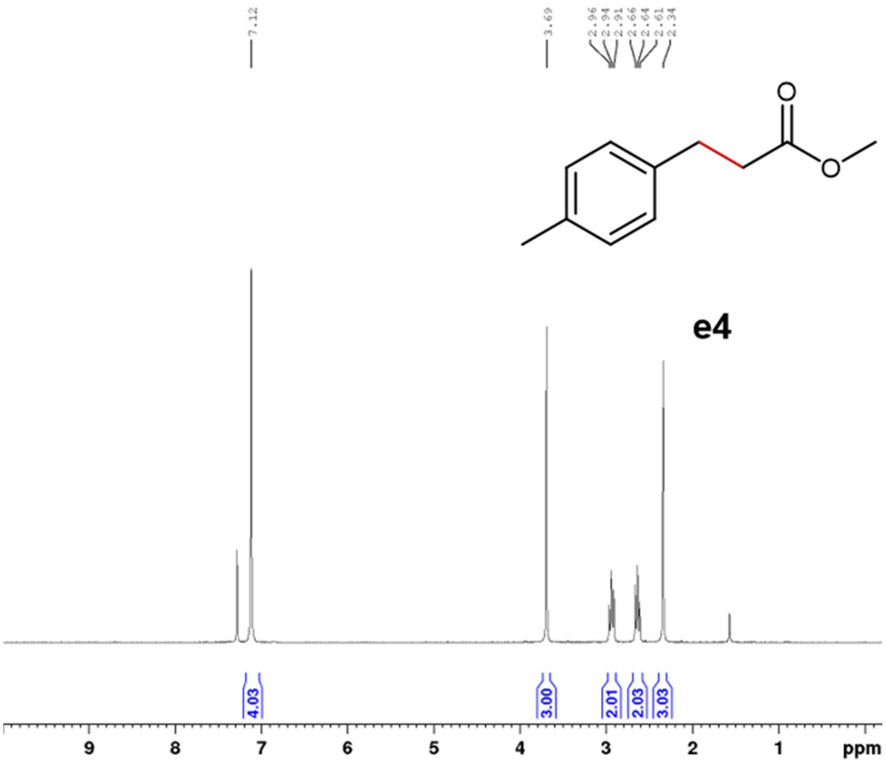

Figure S118. ^{1}H NMR of methyl 3-(p-tolyl)propanoate (**e4**) in $CDCl_3$.

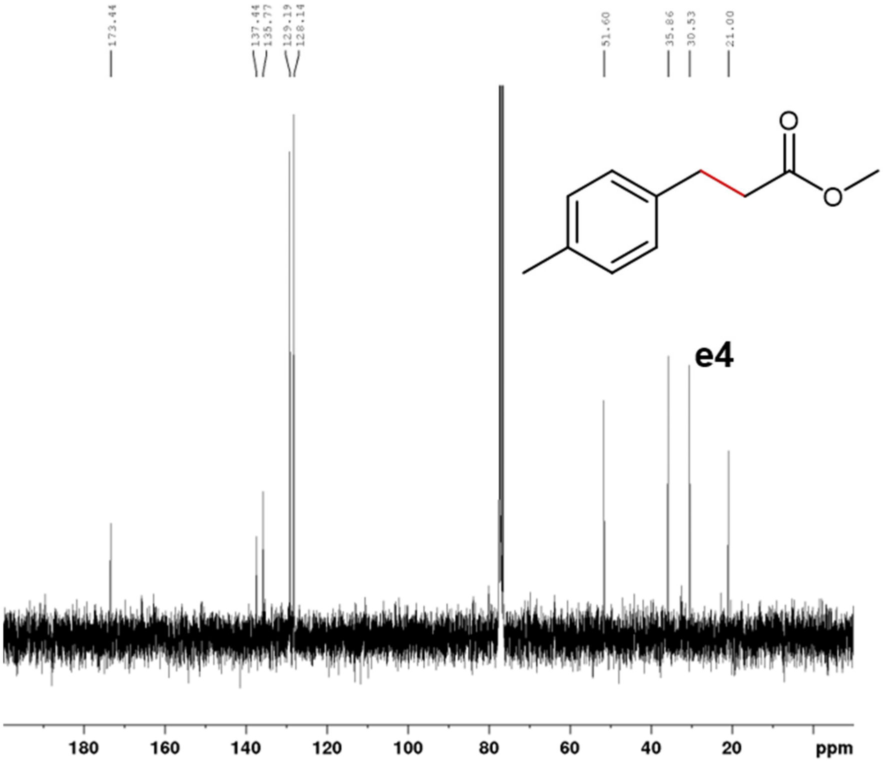

Figure S119. $^{13}C\{^{1}H\}$NMR of methyl 3-(p-tolyl)propanoate (**e4**) in $CDCl_3$.

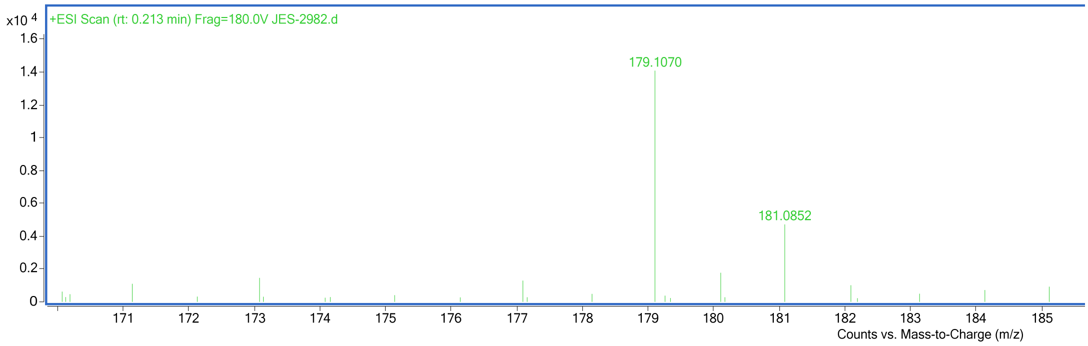

Figure S120. HR-MS of methyl 3-(p-tolyl)propanoate (**e4**).

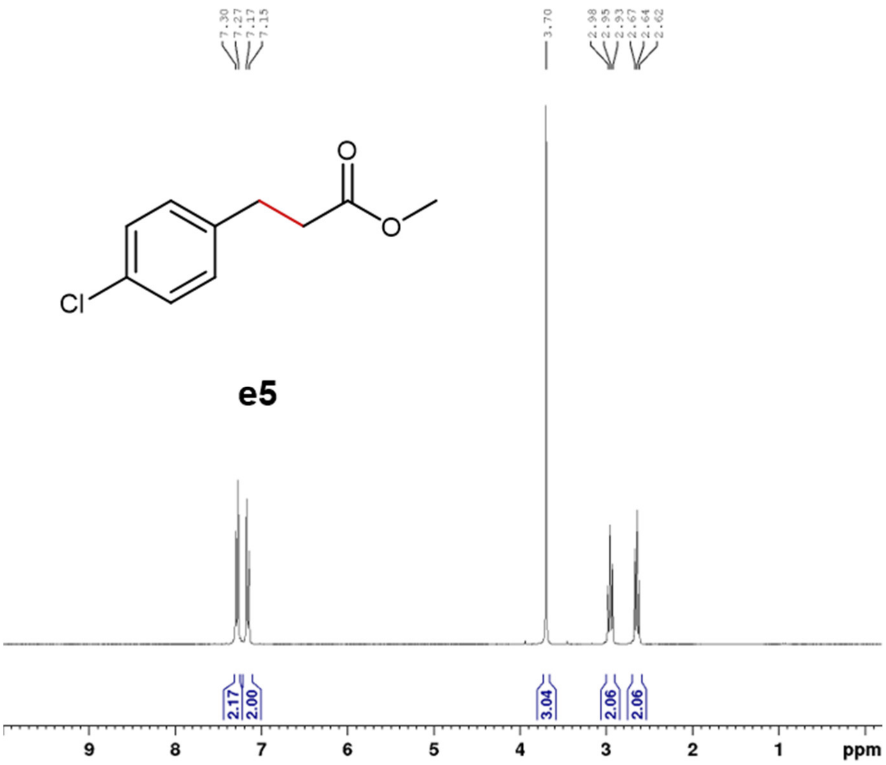

Figure S121. ^{1}H NMR of methyl 3-(4-chlorophenyl)propanoate (**e5**) in $CDCl_3$.

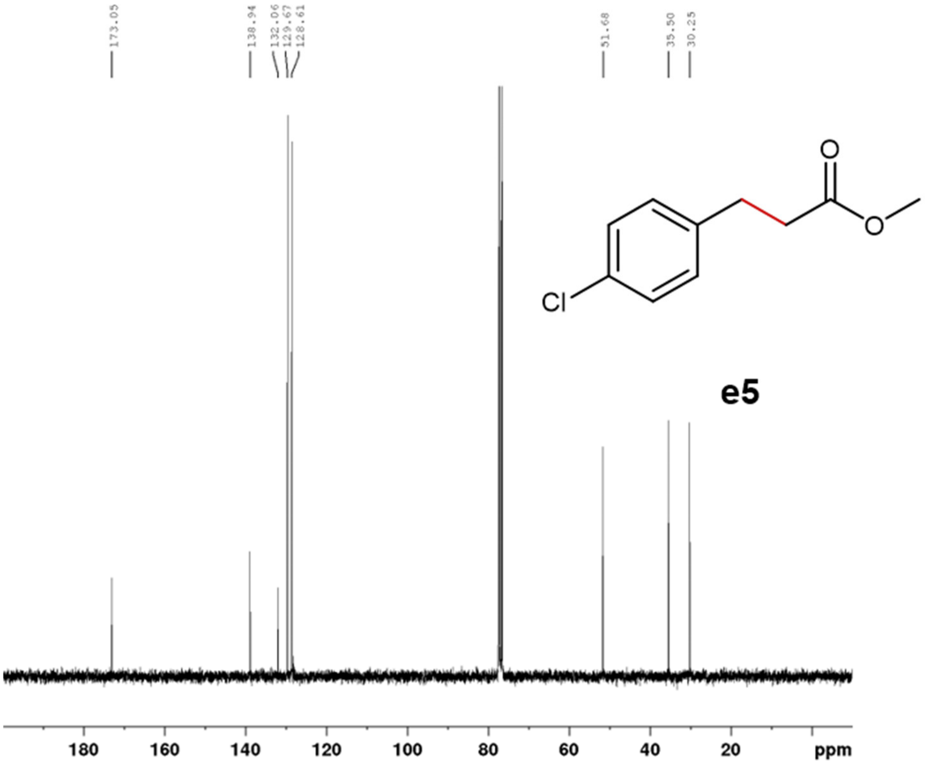

Figure S122. ^{13}C ^{1}H}NMR of methyl 3-(4-chlorophenyl)propanoate (**e5**) in $CDCl_3$.

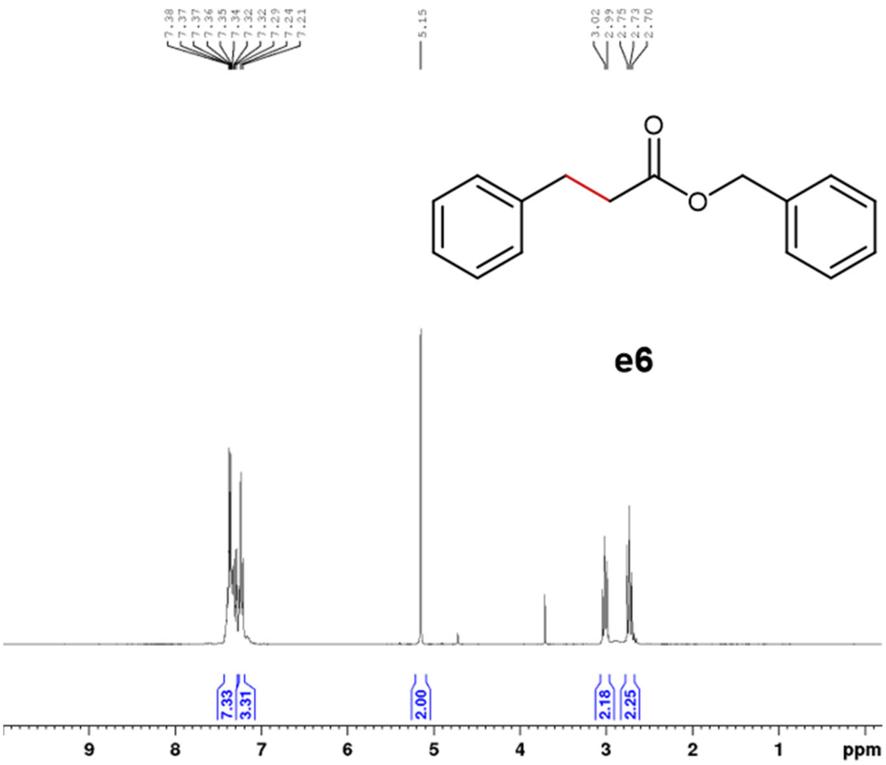

Figure S123. ^{1}H NMR of benzyl 3-phenylpropanoate (**e6**) in $CDCl_3$.

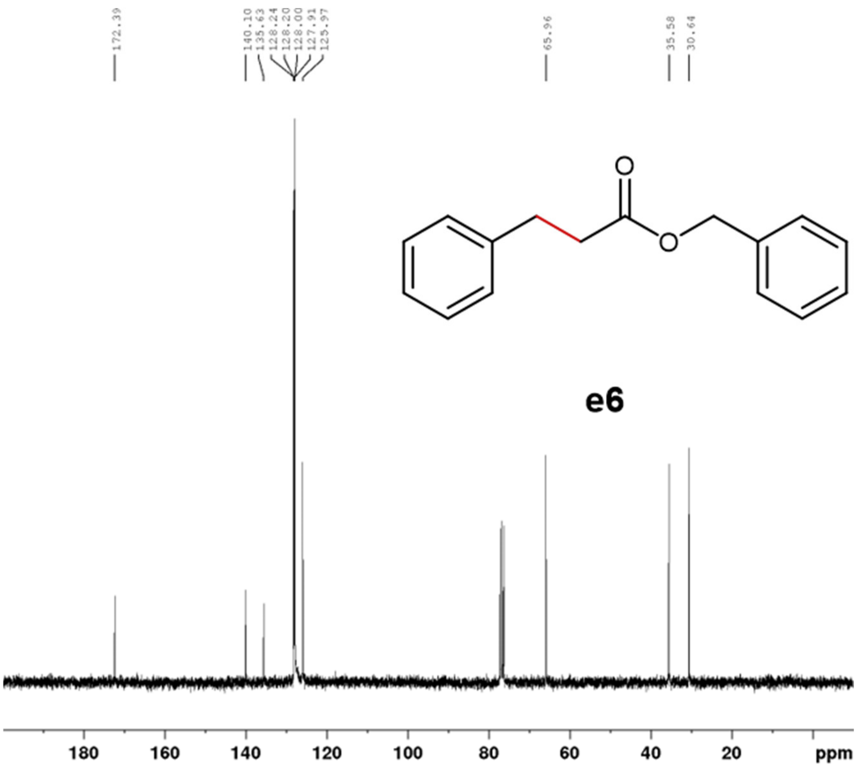

Figure S124. $^{13}C\{^{1}H\}$NMR of benzyl 3-phenylpropanoate (**e6**) in $CDCl_3$.

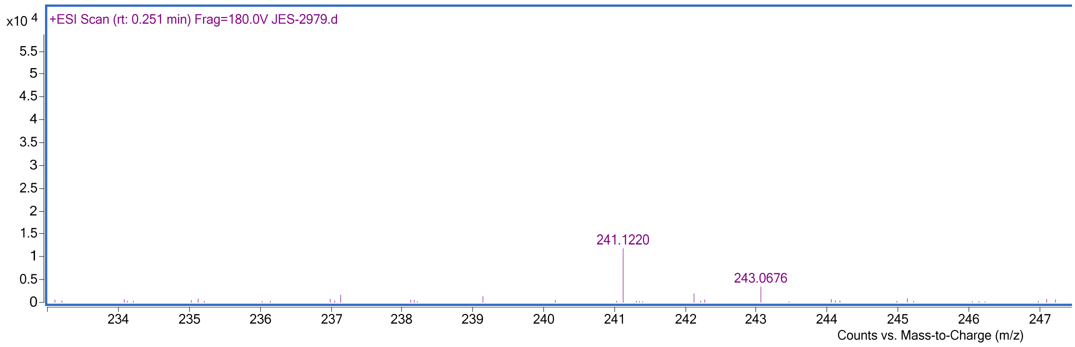

Figure S125. HR-MS of benzyl 3-phenylpropanoate (**e6**).

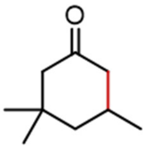

e7

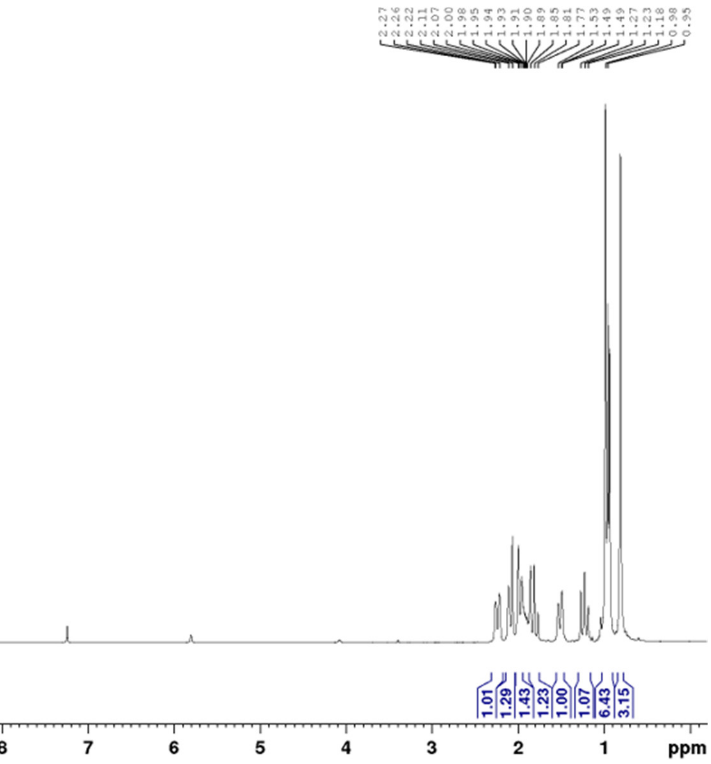

Figure S126. ^{1}H NMR of 3,3,5-trimethylcyclohexan-1-one (**e7**) in $CDCl_3$.

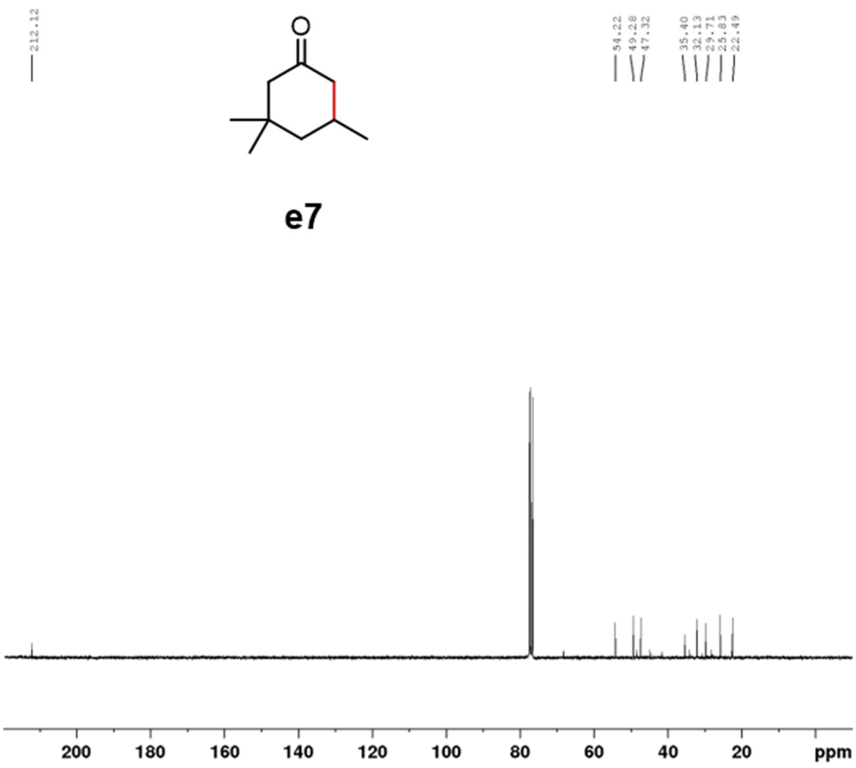

Figure S127. $^{13}C\{^{1}H\}$NMR of 3,3,5-trimethylcyclohexan-1-one (**e7**) in $CDCl_3$.

8. Pictorial Demonstration of Simple Catalyst Separation

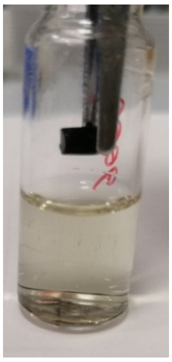

Figure S128. Easy catalyst removement: the loaded pellets are conveniently separated from the reaction solution using simple tweezers and can be reused in the next run(s).

9. References

[1] Woollins, J. D. (Ed.) *Inorganic Experiments, 3rd Edition*. Wiley-VCH: Weinheim, **2010**.

[2] Bruker (2019), *APEX3 v2019.11-0, SAINT V8.40B, SHELXTL-2018*, Bruker Nano, Inc.: Madison (WI), USA, **2019**.

[3] (a) G. M. Sheldrick, *SHELXT-2018: Program for the Solution of Crystal Structures*, University of Göttingen, Germany, **2014**. (b) G. M Sheldrick, *Acta Crystallogr., Sect. A: Found. Crystallogr.* **2008**, *64*, 112−122. (c) G. M. Sheldrick, *Acta Crystallogr., Sect. A: Found. Adv.* **2015**, *71*, 3−8.

[4] (a) G. M. Sheldrick, *SHELXL-2018: Program for the Refinement of Crystal Structures*, University of Göttingen, Germany, **2018**. (b) G. M. Sheldrick, *Acta Crystallogr., Sect. C: Struct. Chem.* **2015**, *71*, 3−8.

[5] C. B. Hübschle, G. M. Sheldrick, B. Dittrich, *Shelxle: A Qt graphical user interface for SHELXL, J. Appl. Crystallogr.* **2011**, *44*, 1281−1284.
